# A new species in the major malaria vector complex sheds light on reticulated species evolution

**DOI:** 10.1038/s41598-019-49065-5

**Published:** 2019-10-14

**Authors:** Maite G. Barrón, Christophe Paupy, Nil Rahola, Ousman Akone-Ella, Marc F. Ngangue, Theodel A. Wilson-Bahun, Marco Pombi, Pierre Kengne, Carlo Costantini, Frédéric Simard, Josefa González, Diego Ayala

**Affiliations:** 1 IBE (CSIC-Universitat Pompeu Fabra), Barcelona, Spain; 2 MIVEGEC, IRD, CNRS, Univ. Montpellier, Montpellier, France; 3 CIRMF, Franceville, Gabon; 4 ANPN, Libreville, Gabon; 5 Università di Roma “Sapienza”, Rome, Italy

**Keywords:** Ecological genetics, Adaptive radiation

## Abstract

Complexes of closely related species provide key insights into the rapid and independent evolution of adaptive traits. Here, we described and studied *Anopheles fontenillei* sp.n., a new species in the *Anopheles gambiae* complex that we recently discovered in the forested areas of Gabon, Central Africa. Our analysis placed the new taxon in the phylogenetic tree of the *An*. *gambiae* complex, revealing important introgression events with other members of the complex. Particularly, we detected recent introgression, with *Anopheles gambiae* and *Anopheles coluzzii*, of genes directly involved in vectorial capacity. Moreover, genome analysis of the new species allowed us to clarify the evolutionary history of the 3La inversion. Overall, *An*. *fontenillei* sp.n. analysis improved our understanding of the relationship between species within the *An*. *gambiae* complex, and provided insight into the evolution of vectorial capacity traits that are relevant for the successful control of malaria in Africa.

## Introduction

Species at earlier speciation stages provide unique insights into the evolutionary forces involved in the origin of new species before signal blurring by demographic and selective processes. However, when closer to the first signals of divergence, it is harder to define the species concept, and to predict whether this process will lead to speciation^1^. Complexes of species and closely related taxa where the species boundaries are uncertain offer a precious opportunity to study the “speciation continuum”^1,2^. Unfortunately, the incomplete reproductive isolation cannot fully prevent introgression between taxa, thus hindering the true phylogenetic relationships^3^. On the other hand, genetic exchanges in backcrossed hybrids can favour adaptation^4^. Indeed, advantageous alleles can be selected in one species and introgressed in another, thus favouring, for instance, range expansion^5^, altitudinal adaptation^6^, and insecticide resistance^7^.

Most of the major malaria vectors worldwide belong to species complexes that include also other non-vector species^8^, providing a compelling opportunity to understand the rapid and independent evolution of their vectorial capacity^9,10^. Indeed, malaria mosquitoes exhibit wide ecological plasticity, preference for feeding on humans, and large population size. Conversely, non-vector species display narrower geographical range, zoophilic host preference, and strong seasonal dependence or reduced population size^11^. In Africa, three of the six major malaria vectors belong to the *Anopheles gambiae* complex*: Anopheles gambiae*, *Anopheles coluzzii*, and *Anopheles arabiensis*^12^. This complex includes eight cryptic species^13–15^ that differ in many ecological aspects, particularly in host feeding preference, breeding sites, feeding behaviour, and role in malaria transmission^13,16^. Most of the *An*. *gambiae* species live in natural habitats, and do not have any or only a secondary role in malaria transmission. Indeed, adaptation to anthropogenic habitats, and therefore a role in human malaria transmission, is an exception rather than the rule within this complex^16^. The *An*. *gambiae* complex is an example of speciation with gene flow where species exhibit extensive genomic introgression (an indication of permeable gene flow barriers among them^3,10^) that is sustained by heterogenic patterns of reproductive isolation^17^. Consequently, pervasive introgression has hindered the elucidation of the correct phylogenetic relationships^18^. Moreover, gene exchanges between species in the complex have modulated their local adaptation capacity. For instance, *An*. *arabiensis* ability to live in desiccating environments has been conferred by introgression of the 2La inversion from *An*. *gambiae*/*An*. *coluzzii*^10,19^. *An*. *coluzzii* has developed resistance to insecticide treatments due introgression of the *kdr* mutation from *An*. *gambiae*^7^. Introgression of genes linked to insecticide resistance has repeatedly occurred in the last decades^20,21^. Thus, introgression has accelerated local adaptation and range expansion within the complex.

Complexes of closely related species also offer a compelling opportunity to study locally adapted alleles. Comparative genomic analysis of recent species radiations allowed unravelling the genetic basis of the traits involved in their ecological, behavioural and genetic divergence^22^. In *Anopheles*, these comparative studies have contributed to elucidate some traits involved in vectorial capacity that in turn could be used to improve vector control strategies^9^. For instance, comparison of the antennal transcriptome profiles of *An*. *gambiae* and *An*. *quadriannulatus* has provided genomic insights into host preference evolution towards humans^23^. Moreover, comparison of genome-wide data of one fresh water (*An*. *gambiae*) and one salt water (*Anopheles melas*) species allowed identifying genomic regions involved in salinity tolerance^24^. These data can be used to develop alternative malaria control strategies by targeting genes involved in reducing vectorial capacity traits, such as human bite rates or local adaptation^10,23,24^. Therefore, understanding the origin and the mechanisms underlying vectorial capacity within the *An*. *gambiae* complex is crucial for the successful control of malaria in Africa^16,25^.

In the present study, we carried out an exploratory survey at La Lopé National Park (Gabon) in 2014. In this survey, we discovered mosquitoes that we initially morphologically identified as *An*. *gambiae*. However, additional bio-ecological, behavioural, taxonomic, cytogenetic, and preliminary molecular analyses suggested the existence of a new taxon in the *An*. *gambiae* complex. Then, genome-wide phylogenetic analysis placed this new taxon in the *An*. *gambiae* complex as a sister species of *Anopheles bwambae*, and in the same clade as *An*. *quadriannulatus*, *An*. *arabiensis*, and *An*. *melas*. Comparative genomic analysis indicated the existence of recent introgression events between the potential new species and *An*. *gambiae/An*. *coluzzii*. These events concerned genes involved in detoxification, desiccation, and olfactory perception functions that are directly linked to local adaptation and host preference. These analyses also elucidated the evolutionary history of the 3La inversion within the complex. Overall, the discovery of this new taxon demonstrates the importance of new species for understanding the evolutionary relationships among the *An*. *gambiae* complex species, with potential implications for elucidating vectorial capacity traits and consequently malaria control.

## Results

In this study, all specimens that were initially morphologically identified as *An*. *gambiae* in the 2014 exploratory survey belonged to a unknown taxon hereafter called *Anopheles* *fontenillei* n.sp. This species is dedicated to our colleague Didier Fontenille, medical entomologist who greatly contributed to the study of mosquitoes and the development of medical entomology in Africa.

### Bio-ecology of *Anopheles fontenillei* sp.n

**Figure 1:**
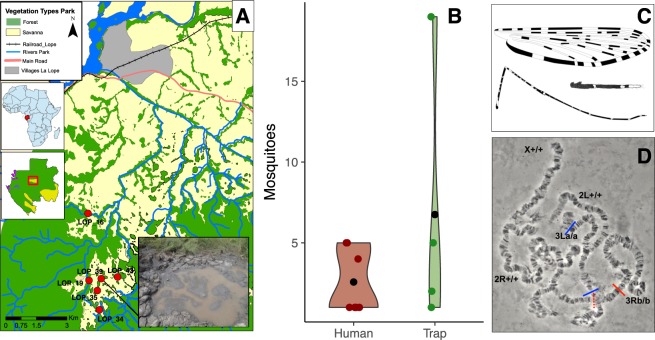
Overview of *An*. *fontenillei* bionomic characteristics. (**A**) Red points indicate the mosquito collection sites in the National Park of La Lopé where *An*. *fontenillei* specimens were captured. The bottom right corner shows a photograph of the breeding site where one larva of the new species was found. (**B**) Mean number (black dots) of *An*. *fontenillei* collected using human landing catch (human, red) vs. BG traps (trap, green) in the six sylvatic sites (**A**) Table S1). (**C**) Morphological features of *An*. *fontenillei*: dorsal view of the wing, maxillary palpus and hindleg with femur, tibia and tarsomeres. (**D**) Images of polytene chromosomes from ovarian nurse cells of *An*. *fontenillei* obtained with a contrast-phase microscope (specimen n. 23). Chromosomal arm karyotypes are indicated following the classical nomenclature^34^. The paracentric inversions are designed by lines (red and blue) above the 3 R(b) and 3 L(a) arms, respectively.

We prospected 22 sites in the National Park of La Lopé in Gabon: 17 sites in the park and 5 sites in the village of La Lopé, 10–15 km away from the park sites. In total, we collected more than 1,500 mosquitoes, belonging to 13 different species. Among these samples, 45 adults and 2 larvae that we morphologically identified as *An*. *gambiae* presented an unexpected DNA band in the PCR assay used for species identification^26^. In Gabon, only three species of the *An*. *gambiae* complex have been recorded, and all of them can be identified based on specific PCR bands^27,28^. The individuals of the unknown species came from six natural sites across the park, away from any human activity or presence (Fig. 1, Table S1). Specifically, these sites were situated at the edge of forest patches and close to natural marshes frequented by wild animals (*e*.*g*., African forest buffalos and other ungulates). In the same breeding sites, we collected also A*n*. *maculipalpis* that breeds in sympatry with *An*. *gambiae*^29^ in sun-exposed, low oxygen, and generally stagnant water. This larval habitat typology is very similar to that of *An*. *gambiae*, *An*. *coluzzii*, and *An*. *arabiensis*^16^, but different from that of the other complex members, such as *An*. *merus* and *An*. *melas* (mangrove swamps) or *An*. *bwambae* (hot thermal springs). This places the new taxon in the fresh-water group of species within the *An*. *gambiae* complex^16^. Although we did not capture any blood-fed mosquito, we assumed a preference for feeding on animals (zoophily) due to the lack of human hosts in these sylvatic sites. However, as mosquitoes were sampled using BG® traps baited with BG-lure (a source of CO_2_)^30^ and Human Landing Catches –HLC– (Fig. 1B), the new species may also feed on humans. Our collections in La Lopé village revealed the presence of two other members of the complex (*An*. *gambiae* and *An*. *coluzzii*), while. We did not find any *An*. *fontenillei* specimen in the village (HLC and larva prospections).

### *Anopheles* (*Cellia*) *fontenillei* sp.n

We preserved five *An*. *fontenillei* specimens for taxonomic purposes (Table S1). The holotype female number LOP3 was collected at La Lopé National Park, site LOP 43. The four females paratypes LOP473, LOP781, LOP1 and LOP2, were also collected at La Lopé National Park, sites LOP 40, LOP 40, LOP 40 and LOP 16, respectively. All the specimens are deposited in the IRD museum collections in Montpellier, France. Overall, *An*. *fontenillei* presented the classical morphotype of species within the *An*. *gambiae* complex^15,31,32^: three white-scaled bands on the maxillary palpus, irregularly shaped speckling on femora and tibiae, and a pale interruption in the third main dark area of wing vein R_1_ (Fig. 1C) (for further taxonomic details see Text S1). However, small differences can be observed among species of the complex. Indeed, we found that the maxillary palpus exhibited a large white-scaled band covering completely palpomere 5 and part of palpomere 4 (Fig. 1C). This morphological trait has been also described in *An*. *bwambae*^33^, although, it is not a discriminant trait with regard to the other members of the complex.

### Cytogenetic analysis

Fixed chromosomal inversions have been used to differentiate species within the *An*. *gambiae* complex since the 1970’s^34^. To confirm the species status and its phylogenetic relationships within the *An*. *gambiae* complex, we then collected 270 sylvatic *Anopheles* specimens for cytogenetic analyses at the LOP 39 site (Fig. 1A). Among the 40 mosquitoes that belonged to the *An*. *gambiae* complex (morphological analysis), only four specimens displayed the new PCR band associated with the new species (see below). Three of these specimens survived to attain the correct stage (half-gravid) to observe polytene chromosomes. According to the classical nomenclature for chromosomal rearrangements in the *An*. *gambiae* complex^34^, all three specimens exhibited the X chromosome and the 2 L arm standard arrangements, and the inversions 3Rb and 3La were fixed. Conversely, the 2Rl inversion was polymorphic: present in one and absent in the other two specimens (Figs 1D, S1). We then used the available molecular karyotyping test^35^ to confirm the presence of the 2La inversion in five additional specimens^35^. All specimens revealed a PCR-band consistent with the 2La standard arrangement, confirming the cytogenetic karyotype. Our data indicate that *An*. *fontenillei* karyotype is similar to that of *An*. *bwambae*^33^. However, the 3Rb inversion might be fixed in the new taxon, while it is polymorphic in *An*. *bwambae*. Further cytogenetic studies in a larger number of individuals will be necessary to confirm the inversion polymorphisms of this species.

### Phylogenetic analysis of the *Anopheles gambiae* complex including *An. fontenillei* sp.n

We first carried out preliminary phylogenetic analysis using 16 specimens to obtain sequences for the nuclear ITS2 and IGS non-coding spacers and the mitochondrial ND5 and COI regions, which are routinely used for *Anopheles* phylogenetic studies^36^. We could amplify and sequence the ITS2 and ND5 regions in nine and five specimens, respectively (Table S1). All regions exhibited low diversity with a unique haplotype, except for the COI gene that displayed five haplotypes. The phylogenetic trees showed that *An*. *fontenillei* sequences always clustered with *An*. *bwambae* within a monophyletic clade (Fig. S2), corroborating the cytogenetic results, but in contrast with the ecological observations. Among the four sequenced regions, ITS2 and ND5 revealed differences between *An*. *fontenillei* and *An*. *bwambae* (Fig. S2). These results are in agreement with previous studies showing that most of the classical molecular markers do not discriminate among species in the complex due to their extensive introgression^10^.

Overall, the new taxon revealed important similarities with *An*. *bwambae*, a thermal spring breeding species from a forested area of Uganda (Semliki valley), at the taxonomical (large band in the palpomeres 4 and 5), cytogenetic (chromosomal inversions), and molecular (sequence divergence) levels. On the other hand, ecological (fresh-water marshes vs thermal springs) and geographical (allopatric distribution: Gabon vs Uganda) results clearly discriminated between *An*. *fontenillei* and *An*. *bwambae*. Thus, additional studies are needed to determine the true phylogenetic place of *An*. *fontenillei* within the *An*. *gambiae* complex.

**Figure 2:**
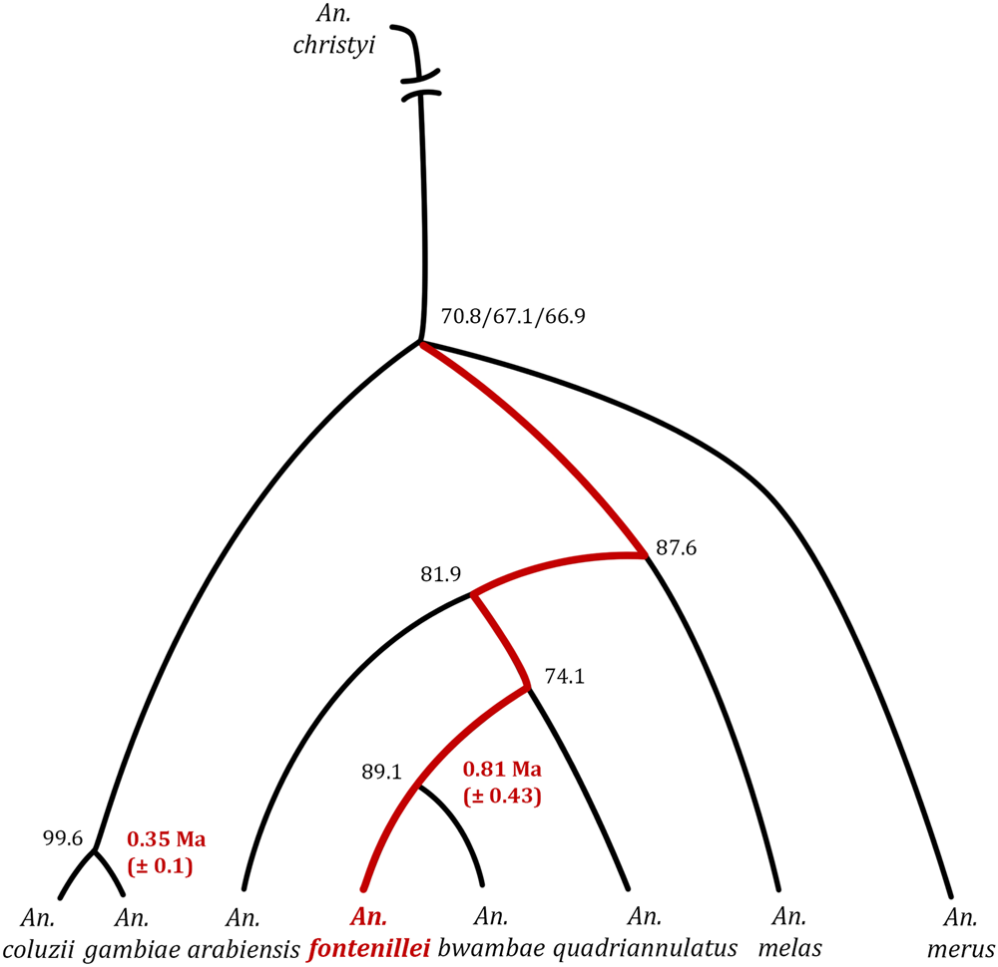
Most common phylogenetic tree topology for the analysed X chromosome sequences of different *An*. *gambiae* complex species. 78 windows in the X chromosome show this tree topology with a weak disagreement in the most basal branch. Black numbers represent bootstrapping values, and red numbers the divergence (Ma, million years ago) estimated based on the pairwise distances of the ML phylogeny and assuming a substitution rate of 11 × 10^−9^ per site, per generation, and 10 generation per year^38^.

We then performed a genome-wide analysis to accurately locate the new species in the phylogenetic tree of the *An*. *gambiae* complex. According to previous studies^10^, we considered that the true species tree is mainly observed in the X chromosome. Hence, we initially focused on the X chromosome, and made a genome assembly of one *An*. *fontenillei* individual sequenced at high coverage (~112X) (Table S2). This assembly was nearly complete (96% of BUSCO complete genes), but made of highly fragmented contigs (N50 = 21 kb) (see Methods, Table S3B and Table S3C). We then added our *An*. *fontenillei* assembly and the highest coverage publicly available *An*. *bwambae* individual (see Methods) to the available multiple alignment file (MAF) based on six described *An*. *gambiae* complex species^10^. We built maximum likelihood (ML) phylogenetic trees for each non-overlapping 50 kb windows (see Methods, Table S4). Following this approach, the relationship among species observed in the X chromosome (Fig. 2) showed that similarly to previous studies, the relative position of the *An*. *merus* and *An*. *coluzzii*-*An*. *gambiae* node was not clearly determined due to incomplete linage sorting^10^. We found that *An*. *fontenillei* appeared as the sister species of *An*. *bwambae* in 83% of the trees (264 out of 319). However, there was some ambiguity in determining the closest taxon of the clade. Indeed, the clade branched with *An*. *quadriannulatus* in 78 of 319 windows (Fig. 2), and with *An*. *arabiensis* in 59 of 319 windows (Fig. S3). If the X chromosome shows the true species tree, we could hypothesize that *An*. *quadriannulatus* or *An*. *arabiensis* shares a common ancestor with *An*. *fontenillei* and *An*. *bwambae*.

To determine whether we could find a stable distinction between *An*. *fontenillei* and *An*. *bwambae*, we repeated the analysis creating a new MAF including the sequences of three more *An*. *fontenillei* and two more *An*. *bwambae* specimens (see Methods). Among the 343 analysed windows, 278 (81%) showed trees where *An*. *fontenillei* and *An*. *bwambae* clustered together but were always separated, indicating that they are different populations and/or species (Fig. S4).

We also estimated the pairwise genetic distance between *An*. *fontenillei* and *An*. *bwambae* and compared it with that between *An*. *coluzzii* and *An*. *gambiae*, the most recently diverged species within the complex (Fig. S5A)^10,37^. The pairwise genetic distance was significantly larger in the *An*. *fontenillei* - *An*. *bwambae* clade than in the *An*. *gambiae* – *An*. *coluzzii* clade (bootstrapping analysis, median 0.0117 and 0.0067 respectively, Fig. S5B, Table S5). Assuming a substitution rate of 1.1 × 10^−9^ per site, per generation, and 10 generations per year^38^, we calculated that the *An*. *fontenillei* - *An*. *bwambae* clade split ~ 0.53 million years ago (Ma), and the *An*. *gambiae* – *An*. *coluzzii* clade diverged ~ 0.31 Ma. This result and the clear ecological distinction between *An*. *fontenillei* and *An*. *bwambae*, suggested that *An*. *fontenillei* is a new species in the *An*. *gambiae* complex rather than an *An*. *bwambae* sub-population.

**Figure 3:**
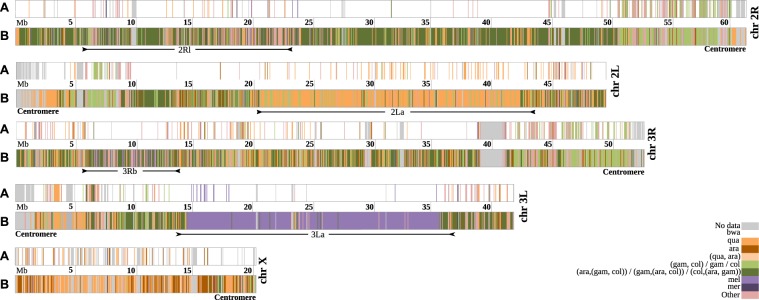
Relationships of *An*. *fontenillei* with other species in the *An*. gambiae complex according to the phylogenetic trees in 50 kb non-overlapping windows along each chromosome arm. For each chromosomal arm, (**A**) refers to after speciation and (**B**) refers to before speciation of *An*. *fontenillei* with *An*. *bwambae*.

We then extended our analysis to the whole genome, and estimated the closest species to *An*. *fontenillei*, before and after speciation with *An*. *bwambae* (Fig. 3). In 84% of the analysed genome, *An*. *bwambae* was the closest species to *An*. *fontenillei*, forming the *An*. *fontenillei *- *An*. *bwambae* (FB) clade, (Fig. 3, after speciation –A–, Fig. S6, Table S5). This proportion was similar in each chromosome arm (from 78.4% in the 3 R to 86.6% in the 3 L chromosome arm), and was similar to the 82.8% observed in the X chromosome, suggesting that *An*. *fontenillei* has not extensively introgressed with other members of the complex in a recent period.

However, the relationship of the FB clade with its closest species and with other clades showed a very different pattern between the X chromosome and the autosomes (Fig. 3, after speciation –A– with *An*. *bwambae*). In the X chromosome, most windows showed the species tree, as previously described^10^. Accordingly, the FB clade was closely related to *An*. *quadriannulatus* (27.5%) and *An*. *arabiensis* (24.5%). In autosomes, most windows showed the recent introgression between *An*. *arabiensis* and the *An*. *gambiae* – *An*. *coluzzii* clade (A(GC) clade)^10^. Moreover, the FB clade branched with the A(GC) clade in 27.6% of windows (Figs 3, S6, Table S6). The next more frequent topology (16.4%) showed the FB clade with *An*. *quadriannulatus* as the closest species. However, when we did not take into account the 2La inversion (see below), this proportion decreased to 9.6%. On the basis of the X chromosome analysis, we could not determine whether *An*. *quadriannulatus* or *An*. *arabiensis* is the closest species to the FB clade, due to the similar number of windows showing one or the other topology (Figs 2, S3). Conversely, in autosomes, the FB clade branched more frequently with the A(GC) clade.

Most of the windows that did not show the FB clade were close to the centromere ends. (~10–11 Mb, Fig. 3, after speciation –A–). In these regions, the proportion of windows showing the FB clades was smaller than in the rest of the chromosome. Moreover, the proportion of trees showing *An*. *fontenillei* close to the GC clade or *An*. *gambiae* or *An*. *coluzzii* was substantially higher close to centromeres than in the rest of the chromosome. Specifically, in regions close to centromeres, the FB clade proportion was ~40% for 2 R and 3 R, and, 51% for the 2 L chromosome arm, while in the rest of the three chromosome arms, the FB clade proportion was >80%. The proportion of trees showing *An*. *fontenillei* close to the GC clade or *An*. *gambiae* or *An*. *coluzzi* in regions close to the centromere was ~20% for the 2R and 3R, and 7% for the 2 L chromosome arm, while on the rest of the three chromosome arms this proportion was <1%. Although the aligned regions were shorter (Fig. S7, Methods), the number of informative positions (16,482 on average) and the alignment quality (proportion of gaps and alignment fragmentation) were higher than in other regions.

While we cannot rule out the hypothesis that these regions are a consequence of incomplete lineage sorting, it is difficult to explain why the FB clade appeared repeatedly close to the GC clade. When we removed the FB clade from the analysis, we did not observe any difference in these regions compared with the rest of the genome. Therefore, these windows might indicate a very recent introgression between *An*. *fontenillei* and *An*. *gambiae* or *An*. *coluzzii*, or both.

### Recent introgressed genes are mainly involved in metabolic detoxification, desiccation, and olfactory perception

We then analysed the gene content of windows where *An*. *fontenillei* instead of branching with *An*. *bwambae* (its closer species), clustered with the major malaria vectors *An*. *gambiae* and *An*. *coluzzii*, or the GC clade. As these species occur in sympatry in the studied area, they could share DNA through secondary contacts. We analysed the three ML tree topologies related with this possible recent introgression separately because the presence of the 2 La polymorphic inversion may affect the results. Indeed, the inversion breaks the more frequently observed GC clade, because the *An*. *coluzzii* individuals used for this study carried predominantly the inversion, while the *An*. *gambiae* individuals harboured mainly the standard arrangement.

We found that 64 windows harboured 198 genes where *An*. *fontenillei* branched with *An*. *gambiae*. Functional enrichment analysis of these genes with DAVID using *An*. *gambiae* genome as background^39,40^ showed four significant clusters (Table 1). The first three clusters were related to cuticle proteins, membrane transporter activity, peptidases and proteases. All these protein families had been linked to metabolic detoxification of insecticides in high-throughput genome-wide studies in several mosquito species (reviewed in^41^). Moreover, cuticle proteins are critical for desiccation tolerance in embryos^42^. Interestingly, the GO terms of the peptidases and proteases cluster have been previously related to high evolutionary rates^9^. The last cluster, was related to heat shock protein 70, a conserved protein related to heat stress, oxidative stress, and detoxification^43,44^. The InterPro domain in this cluster also indicates a rapid evolving gene family (Table 1)^9^.

**Table 1:** Functional enrichment analysis result.

Tree topology	# of windows	# of genes	Term summary	Enrichment Score	GO terms	InterPro domains
(F,G)	64	198	Insect cuticle protein	5.6	GO:0042302	IPR000618
			Transmembrane transporter activity	1.6	GO:0042391, GO:0015701, GO:0015301, GO:0019531, GO:0015106, GO:0015116, GO:0008271, GO:0051453, GO:0005254, GO:1902476, GO:0005887	IPR001902, IPR011547, IPR002645
			Peptidase activity	1.6	GO:0004252	IPR001254, IPR018114, IPR009003
			Heat shock p70	1.4	—	IPR018181, IPR013126
(F,C)	25	62	None	<1.3	—	—
(F,(GC))	35	89	Flavin monooxygenase	3.6	GO:0004499, GO:0050661, GO:0055114, GO:0050660, GO:0004497	IPR000960, IPR020946, IPR023753
			Olfactory receptor	1.3	GO:0050911, GO:0004984, GO:0005549, GO:0005886	IPR004117
(F,L)	15	25	Larval midgut histolysis	4.9	GO:0035069, GO:0097200, GO:0097194, GO:0005737	IPR002138, IPR001309, IPR015917

Significantly enriched clusters (>1.3 enrichment score) obtained by analysis of functional terms with DAVID.

There were 25 windows that contained 62 genes where *An*. *fontenillei* clustered with *An*. *coluzzii*. However, none of these genes was significantly enriched for a particular functional term. Finally, 35 autosomal windows contained 89 genes in which *An*. *fontenillei* branched with the GC clade. If these windows were regions of recent introgression, this would mean that these genes were introgressed between *An*. *fontenillei* and the common ancestors of *An*. *gambiae* and *An*. *coluzzii*. We found two enriched clusters (Table 1): the most significant cluster was enriched in flavin monooxygenase. This protein shows function similarity with the cytochrome P450-monooxygenases^45^ that belong to one of the main protein families related to metabolic detoxification of insecticides in mosquito species (reviewed in^41^). The other significant cluster was related to olfaction. Three of the four GO terms in the cluster (GO:0050911, GO:0004984 and GO:0005549) and the InterPro domain (Table 1) suggest high evolutionary rates^9^.

Following a reverse complementary approach, we also checked whether known mutations that confer resistance to insecticides or to some infections (two traits relevant for malaria transmission) were present in *An*. *fontenillei*. Specifically, we checked 42 mutations in 14 genes related to insecticide resistance^20^ and five mutations in one gene related to immunity and infection resistance^46^. By mapping the four *An*. *fontenillei* individuals to the reference genome (AgamP3) with *bwa*-*mem* (Text S2.4), we found only two mutations in two glutathione S-transferase genes related to insecticide resistance (Table S7): GSTE6 (E89D mutation) and GSTE3 (N73I mutation). Finally, we also checked whether these mutations were present in the other members of the complex. All the available genome references (*An*. *gambia*e Pimperena, *An*. *coluzzii*, *An*. *quadriannulatus*, and *An*. *arabiensis*) showed the wild type alleles (thus, susceptible to insecticides). However, in the MAF made with wild specimens, all the species that could be mapped in those regions (*An*. *gambiae*, *An*. *coluzzii* and the three *An*. *bwambae* individuals) showed the mutant alleles, as did the four *An*. *fontenillei* individuals. This result suggests the presence of ancestral polymorphisms within the complex.

### Chromosome inversions reveal putative introgression events in the *Anopheles gambiae* complex

There are two main inversions in the *An*. *gambiae* complex that shaped its chromosomal evolution and that emerged in our cytogenetic and phylogenetic analysis: the 2La inversion and the 3La inversion^10,47^. The 2La inversion is present in *An*. *arabiensis*, *An*. *gambiae*, and *An*. *coluzzii*^34^, and absent in *An*. *bwambae* and *An*. *fontenillei*., with regard to the 9 specimens cytogenetically and molecularly karyotyped. Consequently, in this region of the 2L chromosome arm, the FB clade was closer to *An*. *quadriannulatus*, defining a well-determined different block that could be easily distinguished in Fig. 3 (line A). According to the four specimens karyotyped, the 3La inversion is fixed in *An*. *fontenillei* as well as in *An*. *bwambae* and *An*. *melas*, as shown by the cytogenetic results. In the 3L chromosome arm, the inverted region could be easily identified because in these windows, the FB clade was closely related to *An*. *melas* (Fig. 3, line A). The inferred breakpoints based on the ML tree topology of the 2La and 3La inversions were inside the known cytological breakpoint ranges, except for the 2L telomeric breakpoint, which was 400 kb shorter (Table S8^34^, VectorBase.org).

**Figure 4:**
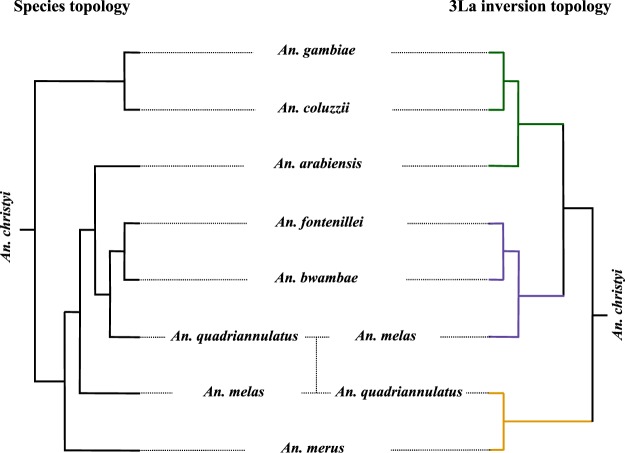
Species topology estimated from the X chromosome sequences compared with the topology of the 3La inversion. *An*. *christyi* was used as outgroup species. Green colour: possible introgression between *An*. *arabiensis* and the *An*. *gambiae* – *An*. *coluzzii* clade common ancestor. Purple colour: species that share the 3La inversion. Yellow colour: possible introgression event between *An*. *quadriannulatus* and *An*. *merus*.

In 45% of windows in the 3L chromosome arm, the three known *An*. *gambiae* complex species with the 3La inversion (*An*. *fontenillei*, *An*. *bwambae*, and *An*. *melas*) were together and separated from the species without the inversion (*An*. *arabiensis*, *An*. *quadriannulatus*, *An*. *merus*, *An*. *gambiae*, and *An*. *coluzzii*) (Figs 4, S6). This topology suggested two introgression events: i) *An*. *arabiensis* with the common ancestor of *An*. *gambiae* and *An*. *coluzzii*, and ii) *An*. *merus* with *An*. *quadriannulatus* (Fig. 4). To date the 3La inversion, we estimated the pairwise distances between *An*. *fontenillei* and *An*. *quadriannulatus* in the 3L chromosome arm outside and inside the inversion (3L: 14.5–35.9 Mb ± the 500 Kb flanking region). Outside the inversion, *An*. *fontenillei* and *An*. *quadriannulatus* diverged 1.4 Ma (±0.91), similar to the estimate based on the more common phylogenetic tree of the X chromosome (1.25 Ma ± 0.54) (Fig. S8, Table S9). The divergence between *An*. *bwambae* and *An*. *quadriannulatus* outside the inversion was also similar to the one estimated for the more common phylogenetic tree of the X chromosome: 1.24 Ma (±0.6). However, the divergence dates estimated inside the inversion between *An*. *fontenillei* and *An*. *quadriannulatus*, and *An*. *bwambae* and *An*. *quadriannulatus* were 2.53 Ma (±0.97) and 2.23 Ma (±0.76), respectively. These estimates were in the range of the estimated date of *An*. *gambiae* complex origin (around 2 ± 0.64 Ma ago). We obtained similar results by repeating the analysis using *An*. *arabiensis* instead of *An*. *quadriannulatus* (Table S9). We could not accurately date the 3La inversion with this method due to the high uncertainty, but we could show that the inversion was at least older than the *An*. *melas*, *An*. *arabiensis*, *An*. *quadriannulatus*, *An*. *bwambae*, and *An*. *fontenillei* group. However, *An*. *arabiensis* and *An*. *quadriannulatus* showed the standard karyotype of the 3La inversion. Therefore, on the basis of the phylogenetic trees, we hypothesized that the ancestral karyotype of the group includes the 3La inversion and that *An*. *quadriannulatus* lost the inversion in the introgression from *An*. *merus*, as already suggested by Fontaine *et al*.^10^, and *An*. *arabiensis* during the introgression with *An*. *gambiae*/*An*. *coluzzii*.

We found some interesting windows in the 3La inversion were *An*. *fontenillei* was more closely related to *An*. *melas* than to *An*. *bwambae* (Fig. 3, before speciation). Functional enrichment analysis of these windows showed that there was only one enriched cluster containing genes related to the stage-specific breakdown of the larval midgut during metamorphosis (Table 1).

Finally, the 3Rb inversion and the 2Rl polymorphic inversions revealed by the karyotyping of *An*. *fontenillei* individuals did not leave a clear pattern in our genomic analysis (see Fig. 3). Both inversions were only shared by *An*. *bwambae*, the closest species to *An*. *fontenillei*, and therefore, big differences are not expected in these regions.

## Discussion

In 1975, the English entomologist G. B. White wrote: “As time passes, it becomes increasingly less likely that other sibling species of this complex (*An*. *gambiae*) will be found”^48^. Indeed, during the last 40 years, only one new species, *An*. *quadriannulatus B* (recently named *An*. *amharicus*), was discovered^15,49^, and *An*. *coluzzii* was separated from *An*. *gambiae*, its sister species^15^. Our field work of 2014 led to the discovery of a new species of the *An*. *gambiae* species complex that we named *An*. *fontenillei* sp.n. Although, we found *An*. *fontenillei* at La Lopé park, a mosaic savanna-forest area of Gabon, this species could also inhabit other mosaic-savanna parts of Gabon and/or Central Africa. Moreover, we cannot exclude its presence in deeper forested areas. This study should warn scientists about the possible presence of this mosquito species when sampling in this part of Africa. On the basis of our collection sites, we hypothesize that this new mosquito species breeds in rain-dependent, sunlit, and open pools, showing similar larval ecology as other fresh-water species within the complex^16^. Although there are not hot spring water or salt-dependent breeding sites at La Lopé, our hypothesis about fresh-water species is limited by the low number of larvae collected (only two). A more extensive larval collection should be carried out to better characterize other potential breeding sites. According to its geographical distribution, we hypothesized a zoophilic host preference (Fig. 1B). This behaviour has already been found in other members of the complex, such as *An*. *quadriannulatus*^50^, and it seems an ancestral character. Although, *An*. *fontenillei* might feed on humans, we did not capture any blood-fed mosquito; therefore, we cannot make strong inferences about its trophic preferences. Nevertheless, the fact that it could be captured with different types of traps suggests a generalist feeding habit with potential consequences on parasite transfer between humans and animals^51^. Indeed, the ancient and recent history of La Lopé provides multiple opportunities for *An*. *fontenillei* to adapt to humans^52^. In the Neolithic age, La Lopé was commonly colonized by nomad tribes for hunting, and in the last century there was a forestry industry in the park. However, more investigations are needed to determine whether this trait is ancestral, or recently acquired (i.e., by introgression, see below) (Table 1).

To determine its phylogenetic position within the complex, we sequenced and *de novo* assembled *An*. *fontenillei* genome. Its analysis allowed us to determine that *An*. *fontenillei* and *An*. *bwambae* are sister species. Pairwise comparisons revealed a higher divergence time between *An*. *fontenillei* and *An*. *bwambae* than between *An*. *gambiae* and *An*. *coluzzii* (Fig. 2)^37^, corroborating the geographical and ecological assumption of two different species (Fig. 1). The *An*. *fontenillei* - *An*. *bwambae* clade was placed together with *An*. *quadriannulatus*, *An*. *arabiensis* and *An*. *melas*, and *An*. *quadriannulatus* or *An*. *arabiensis* were the closest species to the clade (Fig. 2). This is, to date, the most exhaustive phylogenetic tree of the *An*. *gambiae* complex and includes eight of the nine described species (no genome sequence is available for *An*. *amharicus*).

Consistent with Fontaine *et al*.^10^, we found pervasive evidence of introgression in *An*. *fontenillei*, confirming the permeable species boundaries in the *An*. *gambiae* complex^37,53^. Introgression events within species complexes are common in nature, challenging the possibility to trace the evolutionary history of species^3^. We observed patterns of recent introgression between *An*. *fontenillei* and the *An*. *gambiae*-*An*. *coluzzi* clade, particularly in centromeric regions (20% of the phylogenetic trees). These last two species were found in La Lopé village that is close to the sylvatic sites where *An*. *fontenillei* was sampled (Fig. 1A), indicating a potential contact among them. The introgressed genomic windows were mostly enriched in genes associated with detoxification, desiccation tolerance, and olfactory perception (Table 1), traits that have been linked to enhanced vectorial capacity^9^. Indeed, they allow species to live/breed in a broader range of habitats, and blood-feed on different hosts. The evidence of recent gene exchanges between *An*. *gambiae*-*An*. *coluzzii* and other species of the complex may influence the evolution of these two major malaria vectors, with potential consequences for malaria transmission (*i*.*e*., adaptation to sylvatic habitats and/or preference for feeding on animals). However, this patterns of recent introgression events in centromeric regions could be affected by the low recombination rate in these areas that could help to protect introgressed haplotypes for longer time compared with other genomic regions^54^.

Finally, we analyzed the evolution of the 3La inversion in the *An*. *gambiae* complex. While, this inversion was thought to be present in the ancestor of *An*. *melas* and *An*. *bwambae*, we estimated that its origin predated the *An*. *gambiae* complex radiation^33^. Moreover, we hypothesized that the inversion was independently lost by *An*. *arabiensis* and *An*. *quadriannulatus* (Figs 3 and 4). Although the 3La inversion has not been associated with any trait yet, we observed functional enrichment in larval midgut histolysis genes in recently introgressed regions between *An*. *melas* and *An*. *fontenillei* (Table 1). Again, these two species are present in Gabon, and potential gene exchanges could have occurred between them. Chromosomal rearrangements have modulated the evolution of multiple species by affecting local adaptation or speciation^5,55–60^. In our genomic analysis (Fig. 3), we also observed the genomic signature of the 2La inversion that affects the phylogenetic relationship between *An*. *fontenillei*, *An*. *arabiensis*, and *An*. *quadriannulatus*, highlighting the impact of fixed inversions in chromosome evolution within the complex.

Despite the titanic collection efforts led in Africa during the last century, the rainforest of Central Africa has carefully hidden a new piece of the jigsaw of the *An*. *gambiae* complex. The discovery of this new species has provided new insights into genome evolution (*i*.*e*., the 3La inversion) and local adaptation (i.e., salinity tolerance) in this group of closely related species. Moreover, the new species has been an active actor in *An*. *gambiae*-*An*. *coluzzii* evolution, through the exchange of genes involved in vectorial capacity. These introgression events bring new questions about how local populations of *An*. *gambiae* and *An*. *coluzzii*, the major malaria vectors, have been affected. Indeed, adaptation to rainforest habitats, host preference or resting behaviour could have been modified at La Lopé. New studies may provide important information on how vectorial traits have evolved from wild to domestic populations within the complex, with a direct impact for future malaria control strategies.

## Methods

### Research and ethics statements

This study and the methods employed for mosquito sampling were approved by CENAREST (national research authorization AR0013/16/MESRS/CENAREST/CG/CST/CSAR). Specimen collection in the National Parks was approved by a national park entry authorization AE16008/PR/ANPN/SE/CS/AEPN. Finally, the National Research Ethics Committee of Gabon (0031/2014/SG/CNE) authorized the use of the human-landing catch (HLC) method. All volunteers signed the appropriate informed consent documents.

### Mosquito sampling and species identification

Mosquitoes were sampled in the National Park of La Lopé in Gabon, Central Africa, in an exploratory survey in November 2014. Since then, several collections have been carried out in June 2015, February 2016, and November 2016. (Fig. 1, Table S1). Adults were collected using BG traps with BG-lure and a source of CO_2_, and HLC, while larvae were sampled with the dipping method^61^. Collected *Anopheles* mosquitoes were taxonomically identified according to standard morphological features^31,32^. Then, they were individually stored in 1.5 mL tubes at −20 °C and sent to CIRMF for molecular analysis. Total genomic DNA from specimens that had been morphologically identified as belonging to the *An*. *gambiae* complex was extracted using the DNeasy Blood and Tissue Kit (Qiagen), according to the manufacturer’s instructions. Genomic DNA was eluted in 100 μL of TE buffer. A first molecular analysis (PCR-based) performed to identify species within the complex^26^ highlighted the presence of an unspecific fragment of 700 bp. This band did not correspond to any of the species identified using the PCR-RFLP diagnostic test^26^.

### Mosquito karyotyping

Half-gravid females were sampled in November 2016 (Table S1) in forest sites where we previously found specimens belonging to the unspecified taxon. Females were collected by HLC and fed to complete their blood-meal. Mosquitoes were allowed to develop follicles for 25 h at field temperature. Then, ovaries were dissected and stored in Carnoy’s fixative solution (100% ethanol: glacial acetic acid, 3:1 by volume). At the CIRMF, ovaries were squashed in a drop of 50% propionic acid to obtain the polytene chromosomes^62^. The banding patterns of polytene chromosomes were examined using a Leica DM2000 microscope equipped with a Leica DFC 450 camera system (Leica Microsystems GmbH, Wetzlar, Germany). Chromosomal arms and inversions were recorded and scored according to the *An*. *gambiae* chromosome map^63^.

### Preliminary sequencing analysis

To obtain further information about the new 700 bp PCR band, three genomic regions previously employed for phylogenetic studies in the complex were sequenced following the authors’ instructions: internal transcribed spacer subunit 2 (ITS2 ~490 bp^64^), NADH dehydrogenase subunit 5 (ND5 ~300 bp^65,66^), and cytochrome c oxidase subunit I (COI ~495 bp^67^). Moreover, a new set of primers was designed to amplify a fragment of the intergenic spacer gene (IGS ~267 bp): IGSKPF 5′-CTCTTGTGAGAGCAAGAGTGT-3′ and IGSKPR 5′-ATCAAGACAATCAAGTCGAGA-3′. These primers were used also for species identification in the complex. For the IGS gene, PCR reactions were carried out in 25 µl reaction volume than included 1X Qiagen PCR buffer (Qiagen, France), 1.5 mM MgCl2, 200 µM each dNTP (Eurogentec, Belgium), 10 pmol of each primer, 2.5 U Taq DNA polymerase (Qiagen, France) and 1–20 ng of template DNA. Amplifications were performed using a Mastercycler Gradient thermocycler (Eppendorf) with the following conditions: an initial step at 94 °C for 5 minutes followed by 35 cycles of 30 seconds at 94 °C, 30 seconds at 54 °C, 1 minute at 72 °C, and a final elongation step of 10 minutes at 72 °C. Five microliters of the PCR product were visualized by electrophoresis on 1.5% agarose gels containing 0.5 µl/ml ethidium bromide and photographed under UV light.

The sequences obtained for the four regions were analysed using *Geneious* R10^68^. The consensus sequences of each gene were aligned with randomly chosen sequences of each species within the complex. Unique haplotypes were selected to be included in the phylogenetic analysis. The best substitution model for each gene was identified using SMS^69^. Phylogenetic trees were then built using the maximum likelihood (ML) method and PhyML^70^, with nearest neighbour interchange (NNI) for tree searching and approximate likelihood-ratio test (aLRT SH-like,^71^) for branch support. Trees were visualized with iTOL v.3.4.3^72^.

### Genome sequencing and assembly

The genome of four individuals of the unknown species was sequenced using the Illumina platform at the CNAG (Barcelona). To make a *de novo* genome assembly of this species, the genome of one specimen was deeply sequenced to ~112X. The other three specimens were sequenced with an average coverage of ~29X. All reads were paired-end 126 bp long (Table S2).

The genome of the more deeply sequenced *An*. *fontenillei* specimen was assembled at the Bioinformatics Unit, CRG (Barcelona) (Tables S2, S3A). Reads were trimmed and filtered using Skewer, version 0.2.2^73^, to remove the adapter sequence and the low quality part. A FastQC analysis was performed to check the quality of the trimmed reads. Analysis of contaminants using a Kraken database that includes complete bacterial, archaeal, and viral genomes in RefSeq^74^ highlighted the presence of the enterobacteria phage phiX as the only contaminant (Table S3B). Then, the trimmed reads were assembled with the Platanus software version 1.2.4^75^ to produce contigs and scaffolds using the paired-end information. To join the contigs within the same scaffolds, stretches of N needed to be added. These gaps were filled with the Platanus *gap_close* function using the original reads (Table S3A). Then, the scaffolding of the assembled genome was improved using the proteins described for the AgamP4 reference in VectorBase (www.vectorbase.org). Blat^76^ was used to map the proteins to the assembled scaffolds and to reorder and join scaffolds accordingly with PEP_scaffolder^77^. For the second round of gap filling, and due to format incompatibilities, the *GapCloser* tool from the SOAPdenovo package^78^ was used (Table S3A). To evaluate the assembly quality, the presence of conserved genes among the Diptera order was assessed using the BUSCO software^79^. The 2,799 gene models conserved among Diptera species were classified as: i) completely found in a single sequence, ii) fragmented in different sequences, or iii) completely missing. Most of the BUSCO genes (96%) were completely found in a single sequence (Table S3C). Finally, a polishing step was performed by removing the scaffolds that mapped to previously found contaminants (Table S3A).

### Phylogenetic analysis

The available multiple alignment file (MAF) for six species of the *An*. *gambiae* complex, including two outgroup species (*An*. *christyi* and *An*. *epiroticus*^10^) was used to make the genome-wide phylogenetic tree by window analysis. This MAF represented the alignment formed by whole genome sequences from population samples of multiple individuals of *An*. *gambiae*, *An*. *coluzzii*, *An*. *merus*, *An*. *melas*, *An*. *quadriannulatus*, and *An*. *arabiensis*. The *An*. *gambiae* PEST v3 (AgamP3) reference genome obtained from VectorBase (www.vectorbase.org) was also included. Fontaine *et al*.^10^ made a whole multiple genome alignment using ROAST^80^ that represents approximately 40% of the euchromatic genome. The resulting MAF based on field-collected samples was downloaded from http://datadryad.org/resource/doi:10.5061/dryad.f4114^10^. Then, this MAF was added to our *An*. *fontenillei* assembly, and the highest coverage *An*. *bwambae* genome sequences available (see below).

#### *Anopheles fontenillei* sp.n

First, a database was generated with the scaffolds of the *An*. *fontenillei* assembly. Then, blastn was run for each region in the MAF using AgamP3 as query sequence against the *An*. *fontenillei* scaffold database. This blastn analysis was repeated using other species of the MAF regions as query: *An*. *arabiensis*, *An*. *quadriannulatus*, *An*. *melas*, and *An*. *merus* (Text S2.1.1–Text S2.1.4), but not *An*. *coluzzii* and *An*. *gambiae*, due to their similarity to the reference genome AgamP3, or the two outgroup species because they are too divergent. Then, the MAF regions that gave a single hit in any of the species were selected, which represented 63.2% of all the MAF regions for the eight species (Table S10). For the additional MAF region that gave more than one hit, the multiple hits with *e –value* >10^−4^ or with ≤40% of the query covered for each region in each species were excluded (Text S2.1.5), and the sequences that became a unique hit after this filtering were recovered (Table S10). In total, *An*. *fontenillei* could be included in 75.2% of the previous MAF regions, which represents ~30% of the euchromatic genome. For each of these MAF regions, the scaffolds were cut according to the blast result information (Text S2.1.6). Then, these sequences were added to the corresponding MAF region using MAFFT as aligner (v7.221,^81^). The function*–add* was used to modify as less as possible the initial MAF^82^. Finally, each region of the MAF was joined to generate the new MAF that included the *An*. *fontenillei* genome (Text S2.2).

#### *Anopheles bwambae*

The three individual sequences of *An*. *bwambae* available at NCBI were downloaded with fastq-dump: i) *An*. *bwambae 1*, SRR1255391, SRR1255392, and SRR1255303, ii) *An*. *bwambae 3*, SRR1255390, and iii) *An*. *bwambae 4*, SRR1255325. Then, the SRR files for *An*. *bwambae* 1 were joined (Text S2.3). For each individual, the read quality was assessed with fastQC and reads were trimmed using cutadapt (v. 1.8.3^83^)(Text S2.4.1–Text S2.4.3). After trimming, the quality per base was always higher than 24. Then, the trimmed reads were mapped to the AgamP3 reference genome using bwa-mem^84^. Several post-mapping steps were performed, including marking duplicates and realigning around indels using Picard (v.1.109; http://picard.sourceforge.net), samtools (v. 1.3^85^), and GATK (v3.4–46^86^)(Text S2.4.4–Text S2.4.8). Among the three available *An*. *bwambae* individuals, the one with the highest coverage (*An*. *bwambae* 1 with 33.2X) was added to the MAF (coverage for the other two: *An*. *bwambae* 3, 11.7X and *An*. *bwambae* 4, 11.2X). A consensus sequence of the *An*. *bwambae 1* reads mapping to the AgamP3 sequence of every MAF region with the nine species (*An*. *gambiae*, *An*. *coluzzii*, *An*. *merus*, *An*. *melas*, *An*. *quadriannulatus*, *An*. *arabiensis*, *An*. *fontenillei*, *An*. *christyi*, and *An*. *epiroticus*) was created using SAMtools mpileup (Text S2.5). If *An*. *bwambae* reads did not exist for a MAF region, gaps were added to keep the same number of MAF regions as before. This had a marginal effect because it only concerned 0.06% of all MAF regions. Finally, MAFFT aligner, with the function *–add*, was used to add each *An*. *bwambae 1* consensus sequence to the MAF regions, and then all these regions were joined in a new MAF (Text S2.2).

#### MAF with four *An*. *fontenillei* sp.n. individuals and three *An*. *bwambae* individuals

To check the phylogenetic relationship between *An*. *fontenillei* and *An*. *bwambae*, an additional MAF was created that included the eight previously available species, four *An*. *fontenillei* individuals, and three *An*. *bwambae* individuals. Each of the seven individual *An*. *fontenillei* and *An*. *bwambae* genomes was mapped to the AgamP3 reference genome, as described previously for *An*. *bwambae* (Text S2.4). Then, a consensus sequence was generated for each of the new individuals for each of the MAF regions using SAMtools mpileup (Text S2.5). Then, each new sequence was sequentially added to the available multiple alignment regions using the MAFFT *–add* function as aligner (v7.221^81,82^, Text S2.2). Finally, all these sequences were joined in a new MAF file.

#### Window-based phylogenies

From the MAF, 50 kb genome-wide non-overlapping windows were generated (Text S2.6.1). For each window, a ML phylogenetic tree was created using RAxML (v8.2.4,^87^) with the GTRGAMMA model and bootstrapping for 1,000 replicates (Text S2.6.2)^10^. The closer related species, *An*. *christyi*, was used as outgroup because Fontaine *et al*.^10^ already showed that the outgroup choice does not substantially influence the results. Windows with less than 10% of informative base pairs (e.g., <5,000 bp) were excluded (following^10^). The different topologies obtained were sorted, counted, and analysed using ad-hoc perl scripts (Text S2.6.3).

### Pairwise distance and bootstrapping

The R package ‘APE’ (v4.1^88^) was used to estimate pairwise genetic distances based in the ML phylogenetic trees. Then, the bootstrap analysis was performed using the ‘boot’ package in R^89^ (Text S2.7).

### Centromeric region alignment quality

For each chromosomal arm, 30 windows were randomly chosen from centromeric regions, and 30 from other genomic regions. Centromeric regions were defined based on the observed ancestry pattern in Fig. 3: 2L: 0 to 10 Mb, 2R: 50 to 61.3 Mb, 3L: 0 to 10 Mb, 3R: 40 to 53.1 Mb, and X: 15 to 20.2 Mb. For these 60 windows, the alignment length, the alignment length without completely undetermined characters and gaps, the proportion of gaps, and the alignment patterns were extracted from the RAxML information file.

### Data analysis

R v3.2.5 (R Development Core Team, http://cran.r-project.org/) was used to perform all the statistical analyses. Figures were prepared with the Inkscape software (https://inkscape.org).

## Supplementary information

Supplemental Figures, Text and Tables

Supplemental Tables

## Data Availability

DNA sequences have been deposited to GenBank under accessions numbers MN172532 - MN172595. The four *An*. *fontenillei* sp. n. genomes and the genome assembly have been deposited under the bioproject number PRJNA508319.
