# Supplementary material for: A new species in the major malaria vector complex sheds light on reticulated species evolution
Source: Sci Rep. 2019 Oct 14;9:14753. doi: 10.1038/s41598-019-49065-5 (PMC6791875; doi:10.1038/s41598-019-49065-5)
Supplement: Supplementary file 1 — Supplemental Figures, Text and Tables [file 41598_2019_49065_MOESM1_ESM.pdf]

Supplemental Information

Title: A new species in the major malaria vector complex sheds light on reticulated species evolution

Running title: *Anopheles fontenillei*, a new taxon in the *gambiae* complex.

Authors

Maite G Barron[1], Christophe Paupy[2], Nil Rahola[2,3], Ousman Akone-Ella[3], Marc F. Ngangue[3,4], Theodel A. Wilson-Bahun[3], Marco Pombi[5], Pierre Kengne[2], Carlo Costantini[2], Frédéric Simard[2], Josefa Gonzalez[1,*] & Diego Ayala[2,3,*].

Affiliations

[1] IBE (CSIC-Universitat Pompeu Fabra), Barcelona, Spain

[2] MIVEGEC, IRD, CNRS, Univ. Montpellier, Montpellier, France

[3] CIRMF, Franceville, Gabon.

[4] ANPN, Libreville, Gabon

[5] Università di Roma "Sapienza", Rome, Italy

[*] **Co-last and co-corresponding authors:**

Diego Ayala, MIVEGEC, IRD, CNRS, Univ. Montpellier, 911 av Agropolis, BP 64501, 34394 Montpellier, France;

email: diego.ayala@ird.fr.

Josefa González, Institute of Evolutionary Biology (CSIC-Universitat Pompeu Fabra), Passeig Marítim de la Barceloneta 37-49. 08003 Barcelona, Spain;

email: josefa.gonzalez@ibe.upf-csic.es.

Supplementary Information

Text S1. Morphological description of *An. fontenillei* n. sp.
Text S2. Pipeline used to update the available multiple alignment file (MAF) and posterior phylogenetic analysis.

Figure S1. Polytene chromosomes of the three specimens analysed and their karyotypes.

Figure S2. Rooted maximum likelihood phylogenies of *An. fontenillei.* The trees were inferred with phyML (*13*) using the IGS, ITS1, ND5 sequences of *An. fontenillei* and of other members of the *An. gambiae* complex when available. Accession numbers are indicated after the name of the species. The tree was rooted using *An. merus* as an outgroup, when available. Red dots in the middle of the branches indicate bootstrap values >70%.

Figure S3. Second more common phylogenetic tree. 59 windows in the X chromosome showed this tree topology with a week disagreement in in the most basal branch. Black numbers represent bootstrapping values and red numbers the divergence (Mya, million years ago) estimated based on the pairwise distances of the ML phylogeny and assuming a substitution rate of 1.1×10^{-9} per site, per generation, and 10 generation per year [40].

Figure S4. Example of the most common tree when four *An. fontenillei* and three *An. bwambae* individuals were included in the MAF. ML phylogenetic tree with *An. christyi* as an outgroup, six species of the *An. gambiae* complex, three *An. bwambae* individuals (green), and four *An. fontenillei* individuals (yellow).

Figure S5. Differences in the pairwise distance and bootstrapping values between the FB and GC clades. Pairwise distance values between *An. fontenillei* and *An. bwambae* (FB clade), and *An. gambiae* and *A. coluzzii* (GC clade) in all the trees where *An. fontenillei* and *An. bwambae* were the closest in the X chromosome.

Figure S6. Proportion of the different topologies at each chromosome arm and genome-wide. Sections smaller than 1.5% are not marked. 2R: 1.1% (purple); 3R: 1.4% (dark brown); 3R: 0.8% (light brown); 3L: 0.6% (dark red); and genome-wide: 0.8% (light brown).

Figure S7. Alignment quality of centromeric and non-centromeric regions in each chromosome arm. C: Centromeric region (2L:0..10Mb, 2R:50..61.3Mb, 3L:0..10Mb, 3R:40..53.1Mb and X: 15..20.2Mb). O: Other, non-centromeric regions.

Figure S8. Dating the 3La inversion. Divergence time (million years ago, Mya) estimated from pairwise distances assuming a substitution rate of $1.1x10^{-9}$ per site, per generation, and 10 generation per year [40]**.** Pairwise distances were estimated between *An. fontenillei* and *An. quadriannulatus* with the windows inside the inversion (3L-inv), with the windows outside de inversion (3L-out), excluding the 500kb flanking the inversion, and, on the X chromosome more common tree (X). This analysis was repeated for *An. bwambae* and *An. quadriannulatus* as well as for *An. melas* and *An. quadriannulatus*. Additionally, the Mya value based on pairwise distance between *An. melas* and *An. merus* is provided.

Table S1. **Mosquitoes used in this study and their accession numbers.** ID: Specimen identification name; IGS: accession number for the intergenic spacer gene; COI: accession number for the cytochrome oxidase subunit I gene; ND5: accession number for the NADH dehydrogenase subunit 5 gene; ITS2: accession number for the internal transcribed spacer subunit 2 gene.

Table S2. Summary sequence statistics per sample. The table shows the number of reads, mean coverage, mean mapping quality, and read length for the four *An. fontenillei* individuals sequenced in this work.

Table S3. Assembly statistics. A) General assembly, B) Assembly contaminants, and C) BUSCO results.

Table S4. Tree topologies. Number of 50kb windows that showed each tree topology in the genome, by chromosome, and in the 2La and 3La inversions.

Table S5. Pairwise distance and million years (Mya) between the *An. gambiae* - *An. coluzzii* clade (GC) and the *An. fontenillei* - *An. bwambae* clade (FB). Results are for each window of the X chromosome used in the analysis; trees where *An. fontenillei* and *An.*

bwambae were the closest. We assumed a substitution rate of $1.1x10^{-9}$ per site, per generation, and 10 generation per year[40].

Table S6. Number (#) and proportion (%) of windows that represent each phylogenetic tree topology. Results are shown for the whole genome, for the autosomes, and for each chromosome arm. The analysed column is estimated without taking into account the not analysed (NA) windows. F: *An. fontenillei*, B: *An. bwambae*, Q: *An. quadriannulatus*, A: *An. arabiensis*, G: *An. gambiae*, C: *An. coluzzii*, L: *An. melas*.

Table S7. Candidate gene analysis. 42 mutations in 14 genes related to insecticide resistance and 5 mutations in one gene related to immunity and infection resistance were analysed in each *An. fontenillei* individual (nf2, nf3, nf4 and nf5).

Table S8: Comparison of the cytological breakpoints based on the polytene chromosomes with the inferred breakpoints based on tee topology of the inversions. Cytological breakpoints inferred from [36](*), cytological ranges transformed to Mb in VectorBase *An. gambiae* browser (¨), and inferred breakpoints based on tree topology (‘).

Table S9: Dating the 3La inversion. Divergence time (million years ago, Mya) estimated from pairwise distances assuming a substitution rate of $1.1x10^{-9}$ per site, per generation and 10 generation per year [40]. Pairwise distances were estimated between *An. fontenillei* and *An. quadriannulatus* with the windows inside the inversion (3La-inversion), with the windows outside the inversion (3L outside), excluding the 500kb flanking the inversion, and on the X chromosome more common tree. This analysis was repeated for *An. bwambae* and *An. quadriannulatus*, for *An. melas* and *An. quadriannulatus*, for *An. fontenillei* and *An. arabiensis*, for *An. bwambae* and *An. arabiensis*, and for *An. melas* and *An. arabiensis*.

Table S10. MAF regions with one or multiple hits. Number of regions per each chromosome arm, for the whole genome, and genome proportion used to form the MAF or discarded.

Table S1. Mosquitoes used in this study and their accession numbers. ID: Specimen identification name; IGS: accession numbers for intergenic spacer gene ; COI: accession numbers for cytochrome oxidase subunit I gene; ND5: accession numbers for NADH dehydrogenase subunit 5 gene; ITS2: accession numbers for internal transcribed spacer subunit 2 gene.

ID	Date	Site	Lat	Long	Species	Sex	Collection	Purpose	Genome	IGS	COI	ND5	ITS1
LOP646	January_2016	Lop_16	-0.16953	11.58733	An. fontenillei	F	Trap						
LOP137	January_2016	Lop_19	-0.19925	11.58780	An. fontenillei	F	Human						
LOP1112	January_2016	Lop_19	-0.19925	11.58780	An. fontenillei	F	Trap						
LOP17	January_2016	Lop_34	-0.21229	11.59241	An. fontenillei	F	Trap						
LOP37	January_2016	Lop_34	-0.21229	11.59241	An. fontenillei	F	Trap						
LOP173	January_2016	Lop_35	-0.19726	11.59965	An. fontenillei	F	Trap						
LOP179	January_2016	Lop_35	-0.19726	11.59965	An. fontenillei	F	Trap						
LOP493	January_2016	Lop_35	-0.19124	11.59327	An. fontenillei	F	Trap						
LOP516	January_2016	Lop_35	-0.19726	11.59965	An. fontenillei	F	Trap						
LOP837	January_2016	Lop_35	-0.19726	11.59965	An. fontenillei	F	Trap						
LOP83	January_2016	Lop_36	-0.20375	11.59144	An. fontenillei	F	Human						
LOP399	January_2016	Lop_40	-0.19847	11.59318	An. fontenillei	F	Human						
LOP105	January_2016	Lop_43	-0.19774	11.60039	An. fontenillei	F	Human						
LOP107	January_2016	Lop_43	-0.19774	11.60039	An. fontenillei	F	Human						
LOP109	January_2016	Lop_43	-0.19774	11.60039	An. fontenillei	F	Human						
LOP118	January_2016	Lop_43	-0.19774	11.60039	An. fontenillei	F	Human						
LOP5	June_2015	Lop_16	-0.16953	11.58733	An. fontenillei	F	Trap						
LOP473	January_2016	Lop_40	-0.19847	11.59318	An. fontenillei	F	larva	Paratype					
LOP781	January_2016	Lop_40	-0.19847	11.59318	An. fontenillei	F	larva	Paratype					
LOP1	June_2015	Lop_40	-0.19847	11.59318	An. fontenillei	F	Human	Paratype					
LOP2	June_2015	Lop_16	-0.16953	11.58733	An. fontenillei	F	Human	Paratype					
LOP3	June_2015	Lop_40	-0.19847	11.59318	An. fontenillei	F	Human	Holotype					
LOP83	November_2016	Lop_39	-0.19847	11.59318	An. fontenillei	F	Human	Cytogenetic					
LOP230	November_2016	Lop_39	-0.19847	11.59318	An. fontenillei	F	Human						
LOP240	November_2016	Lop_39	-0.19847	11.59318	An. fontenillei	F	Human						
LOP269	November_2016	Lop_39	-0.19847	11.59318	An. fontenillei	F	Human						
LOP23	November_2016	Lop_39	-0.19847	11.59318	An. fontenillei	F	Human	Cytogenetic					
LOP29	November_2016	Lop_39	-0.19847	11.59318	An. fontenillei	F	Human						
LOP35	November_2016	Lop_39	-0.19847	11.59318	An. fontenillei	F	Human	Cytogenetic					
LOP38	November_2016	Lop_42	-0.19847	11.59318	An. fontenillei	F	Human						
LOP18.5	November_2014	Lop_16	-0.16953	11.58733	An. fontenillei	F	Trap						
LOP18.1	November_2014	Lop_16	-0.16953	11.58733	An. fontenillei	F	Trap	Sequencing		MN172580	MN172532	MN172548	MN172564
LOP18.2	November_2014	Lop_16	-0.16953	11.58733	An. fontenillei	F	Trap	Sequencing		MN172581	MN172533	MN172549	MN172565
LOP18.3	November_2014	Lop_16	-0.16953	11.58733	An. fontenillei	F	Trap	Sequencing		MN172582	MN172534	MN172550	MN172566
LOP21.1	November_2014	Lop_16	-0.16953	11.58733	An. fontenillei	F	Trap	Sequencing		MN172583	MN172535	MN172551	MN172567
LOP21.2	November_2014	Lop_16	-0.16953	11.58733	An. fontenillei	F	Trap	Genome	PRJNA508319	MN172584	MN172536	MN172552	MN172568
LOP36.1	November_2014	Lop_16	-0.16953	11.58733	An. fontenillei	F	Trap	Sequencing		MN172585	MN172537	MN172553	MN172569
LOP36.2	November_2014	Lop_16	-0.16953	11.58733	An. fontenillei	F	Trap	Sequencing		MN172586	MN172538	MN172554	MN172570
LOP36.3	November_2014	Lop_16	-0.16953	11.58733	An. fontenillei	F	Trap	Sequencing		MN172587	MN172539	MN172555	MN172571
LOP37.1	November_2014	Lop_16	-0.16953	11.58733	An. fontenillei	F	Trap	Genome	PRJNA508319	MN172588	MN172540	MN172556	MN172572
LOP48.1	November_2014	Lop_16	-0.16953	11.58733	An. fontenillei	F	Trap	Sequencing		MN172589	MN172541	MN172557	MN172573
LOP48.2	November_2014	Lop_16	-0.16953	11.58733	An. fontenillei	F	Trap	Sequencing		MN172590	MN172542	MN172558	MN172574
LOP48.3	November_2014	Lop_16	-0.16953	11.58733	An. fontenillei	F	Trap	Genome	PRJNA508319	MN172591	MN172543	MN172559	MN172575
LOP48.4	November_2014	Lop_16	-0.16953	11.58733	An. fontenillei	F	Trap	Genome	PRJNA508319	MN172592	MN172544	MN172560	MN172576
LOP48.5	November_2014	Lop_16	-0.16953	11.58733	An. fontenillei	F	Trap	Sequencing		MN172593	MN172545	MN172561	MN172577
LOP55.2	November_2014	Lop_16	-0.16953	11.58733	An. fontenillei	F	Trap	Sequencing		MN172594	MN172546	MN172562	MN172578
LOP55.3	November_2014	Lop_16	-0.16953	11.58733	An. fontenillei	F	Trap	Sequencing		MN172595	MN172547	MN172563	MN172579

Table S2. Sequencing summary statistics per sample. The number of reads, mean coverage, mean mapping quality and the read length for the four sequenced An. fontenillei individuals.

ID	Sample Name	Million read-pairs	Mean coverage	Mean Mapping quality*	Read length
nf2	LOP21.2	49.691	28.7519	34.1382	126
nf4	LOP48.3	50.741	30.474	34.5734	126
nf5	LOP48.4	44.888	27.0767	34.545	126
nf3	LOP37.1	262.733	112.01	-	126

*mapped to AgamP3 with trimmed reads

Table S3. Assembly statistics. A) General statistics of the assembly.

Name	n	n:500	L50	min	N80	N50	N20	E-size	max	sum	%N
CONTIGS	#######	136,082	24,237	500	983	2,279	5,954	4,316	98,272	227,100,000	-
SCAFFOLDS	660,102	34,148	3,152	500	5,634	17,851	42,862	27,467	256,104	218,800,000	0.57
AFTER GAP FILLING	660,102	34,161	3,174	500	5,661	17,909	42,803	27,530	256,680	220,900,000	0.21
SCAFFOLDS 2 (with protein	656,433	32,067	2,549	500	6,206	21,566	55,797	34,052	260,210	221,100,000	0.41
AFTER GAP FILLING 2	656,433	32,249	2,548	500	6,236	21,734	56,130	34,296	261,188	222,800,000	0.02
removing PhiX	655,480	32,249	2,548	500	6,236	21,734	56,130	34,296	261,188	222,800,000	0.02

Table S3. Assembly statistics. B) assembly contaminant.

Sample	Pair	Percentage of reads covered by the clade rooted at this taxon	NCBI taxonomy ID	Indented scientific name
report_01_C6DV6ANXX_3_3	1	0.14	374840	Enterobacteria phage phiX174 sensu lato
report_01_C6DV6ANXX_3_3	2	0.13	374840	Enterobacteria phage phiX174 sensu lato
report_02_C6DV6ANXX_3_3	1	0.14	374840	Enterobacteria phage phiX174 sensu lato
report_02_C6DV6ANXX_3_3	2	0.13	374840	Enterobacteria phage phiX174 sensu lato
report_02_C9RHWACXX_1_3	1	0.32	374840	Enterobacteria phage phiX174 sensu lato
report_02_C9RHWACXX_1_3	2	0.30	374840	Enterobacteria phage phiX174 sensu lato

Table S3. Assembly statistics. C) BUSCO results.

Description	Number of genes (%)
Total BUSCO groups searched	2,799 (100)
Complete BUSCOs (C)	2,698 (96.39)
- Complete and single-copy BUSCOs (S)	2,694 (96.25)
- Complete and dulicated BUSCOs (D)	4 (0.14)
Fragmented BUSCOs (F)	68 (2.43)
Missing BUSCOs (M)	33 (1.18)

Table S4. Tree topology. Number of 50kb windows that showed each tree topology in the genome, by chromosome and in 2La and 3La inversions.

**Tree topology**	**2L**	**2La**	**2R**	**3L**	**3La**	**3R**	**X**	**All**
Total	921	429	1157	775	419	966	319	4138
NA	66	5	69	65	9	96	85	381
(O,((((A,(C,G)),(B,K)),Q),(L,R)))	91	0	270	62	0	153	2	578
(O,(((A,(C,G)),(Q,R)),((B,K),L)))	1	0	0	174	174	7	0	182
(O,(((A,(C,G)),((B,K),Q)),(L,R)))	62	1	54	25	0	77	0	218
(O,(((A,((B,K),(C,G))),Q),(L,R)))	40	0	49	17	1	97	4	207
(O,(((((A,(C,G)),(B,K)),Q),L),R))	21	0	88	24	0	36	3	172
(O,((A,(C,G)),(((B,K),L),(Q,R))))	0	0	1	82	82	4	0	87
(O,((((A,(C,G)),(B,K)),L),(Q,R)))	15	0	73	22	1	47	0	157
(O,((((A,C),G),((B,K),Q)),(L,R)))	64	63	0	1	0	1	0	66
(O,(((A,(C,G)),((B,K),L)),(Q,R)))	0	0	6	59	59	3	0	68
(O,(((A,C),R),((((B,K),Q),G),L)))	62	62	0	0	0	0	0	62
(O,(((A,(C,G)),(B,K)),(L,(Q,R))))	8	0	35	18	4	31	0	92
(O,((((A,(C,G)),((B,K),Q)),L),R))	32	1	21	18	0	16	3	90
(O,(((A,C),R),((((B,K),G),Q),L)))	32	32	0	0	0	0	0	32
(O,(((A,(C,G)),(B,K)),((L,R),Q)))	11	0	24	9	1	14	1	59
(O,(((((A,G),C),(B,K)),Q),(L,R)))	4	0	44	9	0	2	0	59
(O,((((A,G),C),(Q,R)),((B,K),L)))	0	0	1	24	24	1	0	26
(O,((((A,((B,K),(C,G))),Q),L),R))	9	0	12	1	0	17	3	42
(O,((((A,(C,G)),Q),(B,K)),(L,R)))	4	0	15	10	2	11	0	40
(O,(((((A,C),G),((B,K),Q)),L),R))	20	20	0	0	0	0	0	20
(O,((A,C),(((((B,K),Q),G),L),R)))	19	19	0	0	0	0	0	19
(O,((((A,((B,K),Q)),L),R),(C,G)))	0	0	0	0	0	0	36	36
(O,(((A,C),G),(((B,K),Q),(L,R))))	15	15	0	0	0	0	0	15
(O,(((((A,(B,K)),Q),L),R),(C,G)))	0	0	0	0	0	1	28	29
(O,((A,(C,G)),((B,K),(L,(Q,R)))))	2	0	10	4	2	11	0	27
(O,((A,C),(((((B,K),G),Q),L),R)))	14	14	0	0	0	0	0	14
(O,(((A,C),G),((((B,K),Q),L),R)))	14	14	0	0	0	0	0	14
(O,((((A,C),G),R),(((B,K),Q),L)))	14	14	0	0	0	0	0	14
(O,((A,(C,G)),((B,K),((L,R),Q))))	4	0	15	2	0	6	0	27
(O,(((A,Q),((B,K),(C,G))),(L,R)))	10	0	6	0	0	11	0	27
(O,(((A,G),C),(((B,K),L),(Q,R))))	0	0	1	12	12	1	0	14
(O,(((((A,(C,G)),(B,K)),Q),R),L))	6	0	14	0	0	6	0	26
(O,(((A,((B,(C,G)),K)),Q),(L,R)))	4	0	3	3	0	16	0	26
(O,((((A,(B,K)),(C,G)),Q),(L,R)))	10	0	5	3	0	4	3	25
(O,((((A,G),C),((B,K),L)),(Q,R)))	0	0	1	12	12	0	0	13
(O,(((A,((B,K),Q)),L),((C,G),R)))	0	0	0	0	0	0	24	24
(O,(((((A,(C,G)),Q),(B,K)),L),R))	1	0	16	5	1	1	0	23
(O,(((((A,G),C),(B,K)),L),(Q,R)))	2	0	16	3	1	2	0	23
(O,(((A,(B,(C,G))),(K,Q)),(L,R)))	4	0	1	8	0	8	0	21
(O,(((A,((B,K),(C,G))),L),(Q,R)))	2	0	5	1	0	13	0	21

(O,((A,(((B,K),Q),(C,G))),(L,R)))	4	0	2	2	1	9	2	19
(O,((((A,(B,K)),Q),L),((C,G),R)))	0	0	0	0	0	0	20	20
(O,(((A,((B,C),(G,K))),Q),(L,R)))	1	0	14	0	0	5	0	20
(O,(((A,C),R),(((B,G),(K,Q)),L)))	10	10	0	0	0	0	0	10
(O,(((((A,(C,G)),(B,K)),L),Q),R))	0	0	15	1	0	2	1	19
(O,((A,(C,G)),(((B,K),Q),(L,R))))	2	1	9	1	0	4	1	17
(O,((((A,((B,K),Q)),L),(C,G)),R))	0	0	0	0	0	0	18	18
(O,((((A,(B,(C,G))),K),Q),(L,R)))	4	0	5	5	0	4	0	18
(O,(((A,(((B,K),G),C)),Q),(L,R)))	2	0	4	0	0	12	0	18
(O,(((((A,C),G),(B,K)),Q),(L,R)))	7	5	5	1	0	0	0	13
(O,(((A,C),R),((((B,K),Q),L),G)))	9	9	0	0	0	0	0	9
(O,(((A,C),(((B,K),Q),G)),(L,R)))	9	9	0	0	0	0	0	9
(O,((((((A,G),C),(B,K)),Q),L),R))	1	0	15	1	0	0	0	17
(O,((((A,C),G),(B,(K,Q))),(L,R)))	9	8	0	0	0	0	0	9
(O,((((A,(C,G)),(B,K)),(Q,R)),L))	1	0	7	4	1	3	0	15
(O,(((A,(((B,C),G),K)),Q),(L,R)))	1	0	11	0	0	4	0	16
(O,(((A,(((B,K),C),G)),Q),(L,R)))	5	0	6	0	0	5	0	16
(O,(((A,C),((((B,K),Q),G),L)),R))	8	8	0	0	0	0	0	8
(O,(((A,(C,G)),(Q,R)),(B,(K,L))))	0	0	0	7	7	1	0	8
(O,(((((A,(B,K)),(C,G)),Q),L),R))	1	0	1	0	0	5	7	14
(O,(((((A,Q),(B,K)),L),R),(C,G)))	0	0	0	0	0	0	14	14
(O,((A,((B,K),(C,G))),((L,R),Q)))	5	0	4	1	0	3	0	13
(O,((A,((B,K),(C,G))),(L,(Q,R))))	2	0	4	2	0	5	0	13
(O,((A,(((B,K),(C,G)),Q)),(L,R)))	5	0	3	0	0	5	0	13
(O,(((A,(B,((C,G),K))),Q),(L,R)))	1	0	1	2	0	9	0	13
(O,((((A,(C,G)),B),(K,Q)),(L,R)))	1	0	4	0	0	7	0	12
(O,((((A,C),G),(((B,K),Q),L)),R))	6	6	0	0	0	0	0	6
(O,(((((A,(B,K)),Q),L),(C,G)),R))	0	0	0	0	0	0	11	11
(O,(((((A,(C,G)),B),K),Q),(L,R)))	0	0	1	7	0	3	0	11
(O,((((A,((B,(C,G)),K)),Q),L),R))	0	0	3	3	0	5	0	11
(O,((((A,C),G),(B,K)),(L,(Q,R))))	2	2	2	0	0	5	0	9
(O,((A,((B,K),((L,R),Q))),(C,G)))	1	0	4	0	0	6	0	11
(O,(((((A,G),C),Q),(B,K)),(L,R)))	0	0	8	1	1	0	0	9
(O,(((((A,(B,(C,G))),K),Q),L),R))	0	0	5	1	0	4	0	10
(O,((((A,(C,G)),((B,K),Q)),R),L))	2	0	1	2	0	5	0	10
(O,((((A,(((B,K),G),C)),Q),L),R))	2	0	3	0	0	5	0	10
(O,(((A,C),R),(((B,(K,Q)),G),L)))	5	5	0	0	0	0	0	5
(O,(((A,C),G),((B,(K,Q)),(L,R))))	5	5	0	0	0	0	0	5
(O,(((((A,C),G),L),((B,K),Q)),R))	5	5	0	0	0	0	0	5
(O,((((A,((B,K),(C,G))),Q),R),L))	1	0	3	1	0	3	1	9
(O,(((A,(B,K)),(C,G)),((L,R),Q)))	5	0	0	0	0	0	4	9
(O,((((A,C),G),(Q,R)),((B,K),L)))	0	0	0	4	4	1	0	5
(O,(((A,(C,G)),Q),((B,K),(L,R))))	1	0	3	2	1	2	0	8
(O,(((A,(C,G)),(B,K)),((L,Q),R)))	1	0	3	3	0	2	0	9
(O,(((A,(C,G)),L),((B,K),(Q,R))))	1	1	1	0	0	6	0	8
(O,(((A,(C,G)),(B,(K,L))),(Q,R)))	0	0	0	4	4	0	0	4

(O,((((A,G),C),(B,K)),(L,(Q,R))))	1	0	5	1	0	1	0	8
(O,((((A,((B,G),C)),K),Q),(L,R)))	2	0	2	2	0	2	0	8
(O,(((A,(C,G)),(B,(K,Q))),(L,R)))	2	0	0	2	0	4	0	8
(O,((((((A,C),G),(B,K)),Q),L),R))	3	3	2	0	0	0	0	5
(O,((A,C),((((B,K),Q),(L,R)),G)))	4	4	0	0	0	0	0	4
(O,((A,C),(((((B,K),Q),L),G),R)))	4	4	0	0	0	0	0	4
(O,(((A,C),((((B,K),G),Q),L)),R))	4	4	0	0	0	0	0	4
(O,((((A,K),(B,(C,G))),Q),(L,R)))	0	0	3	0	0	3	1	7
(O,((((A,Q),(B,K)),L),((C,G),R)))	0	0	0	0	0	0	7	7
(O,((((A,(C,G)),(B,K)),(L,Q)),R))	2	0	4	0	0	1	0	7
(O,((A,(C,G)),(((B,K),(Q,R)),L)))	0	0	2	1	1	3	0	6
(O,(((A,G),C),((B,K),(L,(Q,R)))))	1	0	3	0	0	3	0	7
(O,((((A,((B,C),G)),K),Q),(L,R)))	0	0	4	0	0	3	0	7
(O,((((A,G),C),(B,K)),((L,R),Q)))	1	0	6	0	0	0	0	7
(O,(((A,(B,K)),((C,G),Q)),(L,R)))	4	0	0	0	0	1	1	6
(O,((((A,Q),(C,G)),(B,K)),(L,R)))	1	0	0	1	0	3	1	6
(O,(((((A,(K,Q)),L),B),R),(C,G)))	0	0	0	0	0	0	6	6
(O,((A,(C,G)),((B,(K,L)),(Q,R))))	0	0	0	3	3	0	0	3
(O,((((A,(B,(C,G))),(K,Q)),L),R))	1	0	1	3	0	1	0	6
(O,(((A,C),G),((B,K),(L,(Q,R)))))	2	2	0	0	0	2	0	4
(O,(((A,(B,((C,K),G))),Q),(L,R)))	1	0	0	0	0	5	0	6
(O,(((((A,((B,C),G)),K),Q),L),R))	2	0	3	1	0	0	0	6
(O,((((A,(C,G)),L),(B,K)),(Q,R)))	1	1	4	0	0	0	0	5
(O,((A,C),((((B,K),Q),G),(L,R))))	3	3	0	0	0	0	0	3
(O,((((A,C),(((B,K),G),Q)),L),R))	3	3	0	0	0	0	0	3
(O,(((((A,C),G),((B,K),Q)),R),L))	3	3	0	0	0	0	0	3
(O,(((A,(K,Q)),(B,(C,G))),(L,R)))	2	0	1	0	0	1	1	5
(O,((A,(((B,K),Q),(L,R))),(C,G)))	1	0	2	0	0	1	1	5
(O,((((A,Q),((B,K),(C,G))),L),R))	0	0	1	0	0	3	1	5
(O,(((A,((B,K),Q)),(C,G)),(L,R)))	0	0	0	0	0	3	2	5
(O,(((A,(B,K)),((L,R),Q)),(C,G)))	0	0	0	0	0	1	4	5
(O,(((A,C),G),(((B,K),L),(Q,R))))	0	0	0	2	2	1	0	3
(O,(((A,(B,(C,(G,K)))),Q),(L,R)))	1	0	2	1	0	1	0	5
(O,((A,((B,K),((C,G),Q))),(L,R)))	1	0	0	3	0	1	0	5
(O,((A,(C,G)),(((B,K),(L,R)),Q)))	0	0	4	0	0	1	0	5
(O,(((A,G),C),((B,K),((L,R),Q))))	0	0	4	0	0	1	0	5
(O,(((((A,(C,G)),B),K),L),(Q,R)))	0	0	1	2	0	2	0	5
(O,(((((A,(C,G)),K),B),Q),(L,R)))	1	0	2	2	0	0	0	5
(O,(((((A,(C,G)),Q),(B,K)),R),L))	0	0	5	0	0	0	0	5
(O,(((A,(((B,K),Q),(C,G))),L),R))	0	0	2	0	0	1	1	4
(O,(((A,(B,K)),(C,G)),(L,(Q,R))))	1	0	0	0	0	2	1	4
(O,(((((A,Q),(B,K)),L),(C,G)),R))	0	0	0	1	0	0	3	4
(O,(((((A,(B,K)),Q),(C,G)),L),R))	1	0	0	0	0	0	3	4
(O,(((A,L),(((B,K),Q),(C,G))),R))	0	0	0	0	0	0	4	4
(O,(((A,(K,Q)),L),((B,(C,G)),R)))	0	0	0	0	0	0	4	4
(O,((((A,L),((B,K),Q)),R),(C,G)))	0	0	0	0	0	0	4	4

(O,((((A,(B,K)),Q),(C,G)),(L,R)))	0	0	0	0	0	0	4	4
(O,((((A,G),C),Q),(((B,K),L),R)))	0	0	0	2	2	0	0	2
(O,((((A,C),G),R),(((B,K),L),Q)))	0	0	0	2	2	0	0	2
(O,((((A,C),G),((B,K),L)),(Q,R)))	0	0	0	2	2	0	0	2
(O,((((A,(C,G)),Q),R),((B,K),L)))	0	0	0	2	2	0	0	2
(O,((A,((L,R),Q)),((B,K),(C,G))))	2	0	1	0	0	1	0	4
(O,(((A,((B,G),(C,K))),Q),(L,R)))	1	0	2	0	0	1	0	4
(O,((((A,((B,K),(C,G))),L),Q),R))	1	0	2	0	0	1	0	4
(O,(((A,(C,G)),B),((K,L),(Q,R))))	0	0	3	0	0	1	0	4
(O,((((A,(C,G)),(Q,R)),L),(B,K)))	0	0	0	1	1	2	0	3
(O,(((A,(C,G)),((L,R),Q)),(B,K)))	0	0	1	1	0	2	0	4
(O,((A,(L,(Q,R))),((B,K),(C,G))))	1	0	1	0	0	2	0	4
(O,((((A,(C,G)),(B,K)),R),(L,Q)))	1	0	1	0	0	2	0	4
(O,((((A,(C,G)),(B,K)),(L,R)),Q))	1	0	1	0	0	2	0	4
(O,((((((A,(C,G)),B),K),Q),L),R))	1	0	1	0	0	2	0	4
(O,(((A,(((B,G),K),C)),Q),(L,R)))	0	0	2	0	0	2	0	4
(O,(((A,(((B,G),C),K)),Q),(L,R)))	0	0	1	0	0	3	0	4
(O,((((A,Q),(B,K)),(C,G)),(L,R)))	0	0	1	0	0	3	0	4
(O,(((((A,C),G),(B,K)),L),(Q,R)))	2	1	0	1	0	0	0	3
(O,((((A,(C,G)),B),K),(L,(Q,R))))	0	0	2	2	0	0	0	4
(O,((((A,G),C),((B,K),Q)),(L,R)))	2	0	2	0	0	0	0	4
(O,((A,C),((((B,K),(Q,R)),L),G)))	2	2	0	0	0	0	0	2
(O,((A,C),((((B,G),(K,Q)),L),R)))	2	2	0	0	0	0	0	2
(O,(((A,C),L),((((B,K),Q),G),R)))	2	2	0	0	0	0	0	2
(O,(((A,C),G),((((B,K),Q),R),L)))	2	2	0	0	0	0	0	2
(O,(((A,C),(L,R)),(((B,K),Q),G)))	2	2	0	0	0	0	0	2
(O,(((A,C),((B,(K,Q)),G)),(L,R)))	2	2	0	0	0	0	0	2
(O,((((A,C),R),L),(((B,K),Q),G)))	2	2	0	0	0	0	0	2
(O,((((A,C),R),G),(((B,K),Q),L)))	2	2	0	0	0	0	0	2
(O,((((A,C),G),L),(((B,K),Q),R)))	2	2	0	0	0	0	0	2
(O,(((((A,C),G),R),(B,(K,Q))),L))	2	2	0	0	0	0	0	2
(O,(((((A,C),G),(B,(K,Q))),L),R))	2	2	0	0	0	0	0	2
(O,(((((A,C),G),((B,Q),K)),L),R))	2	2	0	0	0	0	0	2
(O,(((((((A,G),C),B),K),Q),L),R))	1	0	2	0	0	0	0	3
(O,(((A,(L,R)),((B,K),Q)),(C,G)))	0	0	1	0	0	1	1	3
(O,((((((A,G),C),Q),(B,K)),L),R))	0	0	1	1	1	0	0	2
(O,(((A,(C,G)),Q),(((B,K),L),R)))	1	0	0	1	1	0	0	2
(O,(((((A,G),C),(B,K)),(Q,R)),L))	1	0	0	1	1	0	0	2
(O,(((((A,C),G),(Q,R)),(B,K)),L))	0	0	0	1	1	1	0	2
(O,((A,Q),(((B,K),(L,R)),(C,G))))	0	0	1	1	0	1	0	3
(O,((A,(C,G)),((((B,K),Q),R),L)))	0	0	1	1	0	1	0	3
(O,(((A,((B,K),(C,G))),(Q,R)),L))	0	0	1	1	0	1	0	3
(O,((((A,(B,(C,G))),K),L),(Q,R)))	0	0	1	1	0	1	0	3
(O,((((A,((B,C),G)),(K,Q)),L),R))	0	0	0	2	0	1	0	3
(O,(((A,(C,G)),(L,R)),((B,K),Q)))	1	0	1	0	0	1	0	3
(O,((((A,(C,G)),Q),(L,R)),(B,K)))	0	0	2	0	0	1	0	3

(O,((((A,(((B,K),C),G)),Q),R),L))	0	0	2	0	0	1	0	3
(O,(((((A,(B,C)),(G,K)),Q),L),R))	0	0	2	0	0	1	0	3
(O,(((((A,C),G),(B,K)),(Q,R)),L))	1	1	0	0	0	1	0	2
(O,(((((A,K),(B,(C,G))),Q),L),R))	2	0	0	0	0	1	0	3
(O,(((A,((B,(G,K)),C)),Q),(L,R)))	0	0	1	0	0	2	0	3
(O,((((A,((C,G),K)),B),Q),(L,R)))	0	0	1	0	0	2	0	3
(O,((A,((B,(C,G)),K)),((L,R),Q)))	1	0	0	0	0	2	0	3
(O,(((A,(B,(C,G))),(K,L)),(Q,R)))	1	0	0	0	0	2	0	3
(O,(((A,((L,R),Q)),(B,K)),(C,G)))	1	0	0	0	0	2	0	3
(O,((((A,((B,C),(G,K))),Q),L),R))	1	0	0	0	0	2	0	3
(O,(((A,((B,(C,K)),G)),Q),(L,R)))	0	0	0	0	0	3	0	3
(O,(((A,(C,G)),L),(((B,K),Q),R)))	1	0	1	1	0	0	0	3
(O,(((A,(((B,K),(C,G)),Q)),L),R))	0	0	2	1	0	0	0	3
(O,((((A,(B,((C,G),K))),Q),L),R))	0	0	2	1	0	0	0	3
(O,(((((A,(C,G)),B),Q),K),(L,R)))	0	0	2	1	0	0	0	3
(O,((((A,C),G),(B,K)),((L,R),Q)))	1	1	0	1	0	0	0	2
(O,((((((A,C),G),B),K),Q),(L,R)))	1	1	0	1	0	0	0	2
(O,(((((A,(B,K)),(C,G)),Q),R),L))	2	0	0	1	0	0	0	3
(O,((((((A,G),C),(B,K)),Q),R),L))	0	0	1	2	0	0	0	3
(O,((A,(B,(C,(G,K)))),((L,R),Q)))	1	0	2	0	0	0	0	3
(O,(((A,(((B,C),K),G)),Q),(L,R)))	0	0	3	0	0	0	0	3
(O,((((A,((B,C),G)),K),L),(Q,R)))	0	0	3	0	0	0	0	3
(O,((A,Q),((B,K),((C,G),(L,R)))))	0	0	0	0	0	1	1	2
(O,(((A,Q),((B,(C,G)),K)),(L,R)))	0	0	0	0	0	1	1	2
(O,((((A,(B,K)),(L,Q)),(C,G)),R))	0	0	0	0	0	1	1	2
(O,((A,((B,K),(L,(Q,R)))),(C,G)))	0	0	0	1	0	0	1	2
(O,((((A,(B,K)),(C,G)),(L,R)),Q))	0	0	0	1	0	0	1	2
(O,((A,((B,K),Q)),((C,G),(L,R))))	0	0	0	0	0	0	2	2
(O,(((A,L),((B,K),Q)),((C,G),R)))	0	0	0	0	0	0	2	2
(O,(((A,(((B,K),Q),L)),(C,G)),R))	0	0	0	0	0	0	2	2
(O,((((A,(K,Q)),L),(B,(C,G))),R))	0	0	0	0	0	0	2	2
(O,((((A,(B,K)),(L,Q)),R),(C,G)))	0	0	0	0	0	0	2	2
(O,(((((A,Q),K),L),B),((C,G),R)))	0	0	0	0	0	0	2	2
(O,(((((A,Q),(B,K)),R),L),(C,G)))	0	0	0	0	0	0	2	2
(O,(((((A,L),(K,Q)),B),R),(C,G)))	0	0	0	0	0	0	2	2
(O,(((((A,K),Q),L),B),((C,G),R)))	0	0	0	0	0	0	2	2
(O,(((((A,K),Q),B),L),((C,G),R)))	0	0	0	0	0	0	2	2
(O,(((((A,(B,K)),(C,G)),L),Q),R))	0	0	0	0	0	0	2	2
(O,((((A,C),G),(Q,R)),(B,(K,L))))	0	0	0	1	1	0	0	1
(O,((((A,(C,G)),Q),((B,K),L)),R))	0	0	0	1	1	0	0	1
(O,((((A,((B,Q),(C,G))),K),R),L))	0	0	0	1	1	0	0	1
(O,(((((A,G),C),Q),R),((B,K),L)))	0	0	0	1	1	0	0	1
(O,((((((A,C),Q),G),(B,K)),R),L))	0	0	0	1	1	0	0	1
(O,((A,(((B,(C,G)),K),Q)),(L,R)))	0	0	0	1	0	1	0	2
(O,((((A,(C,G)),B),R),((K,Q),L)))	0	0	0	1	0	1	0	2
(O,((A,(((B,K),(Q,R)),L)),(C,G)))	0	0	1	0	0	1	0	2

(O,(((A,(C,G)),B),(K,(L,(Q,R)))))	0	0	1	0	0	1	0	2
(O,(((A,(C,(G,K))),(B,Q)),(L,R)))	0	0	1	0	0	1	0	2
(O,(((A,((K,Q),(L,R))),B),(C,G)))	0	0	1	0	0	1	0	2
(O,((((A,G),((B,K),C)),Q),(L,R)))	0	0	1	0	0	1	0	2
(O,((((A,((B,G),(C,K))),Q),L),R))	0	0	1	0	0	1	0	2
(O,(((((A,K),(C,G)),B),Q),(L,R)))	0	0	1	0	0	1	0	2
(O,(((((A,G),C),((B,K),Q)),L),R))	0	0	1	0	0	1	0	2
(O,((A,(((B,K),G),C)),((L,R),Q)))	1	0	0	0	0	1	0	2
(O,((((A,(B,K)),(C,G)),L),(Q,R)))	1	0	0	0	0	1	0	2
(O,((((A,(B,K)),((C,G),Q)),L),R))	1	0	0	0	0	1	0	2
(O,((((A,(((B,C),G),K)),Q),R),L))	1	0	0	0	0	1	0	2
(O,((A,L),(((B,K),(C,G)),(Q,R))))	0	0	0	0	0	2	0	2
(O,((A,(L,R)),(((B,K),Q),(C,G))))	0	0	0	0	0	2	0	2
(O,((A,(B,(C,G))),((K,Q),(L,R))))	0	0	0	0	0	2	0	2
(O,(((A,(B,(C,G))),K),(L,(Q,R))))	0	0	0	0	0	2	0	2
(O,((((A,K),(B,(C,G))),L),(Q,R)))	0	0	0	0	0	2	0	2
(O,((((A,(C,(G,K))),B),Q),(L,R)))	0	0	0	0	0	2	0	2
(O,((((A,(((B,G),C),K)),Q),L),R))	0	0	0	0	0	2	0	2
(O,(((((A,B),(C,G)),K),Q),(L,R)))	0	0	0	0	0	2	0	2
(O,(((A,((C,G),Q)),(B,K)),(L,R)))	0	0	1	1	0	0	0	2
(O,(((A,((C,G),K)),B),(L,(Q,R))))	0	0	1	1	0	0	0	2
(O,(((A,((C,G),K)),(B,Q)),(L,R)))	0	0	1	1	0	0	0	2
(O,((((A,G),C),(B,K)),((L,Q),R)))	0	0	1	1	0	0	0	2
(O,((((A,C),((B,K),G)),Q),(L,R)))	0	0	1	1	0	0	0	2
(O,((((A,((C,K),G)),B),Q),(L,R)))	0	0	1	1	0	0	0	2
(O,(((((A,(C,G)),B),(K,Q)),L),R))	0	0	1	1	0	0	0	2
(O,(((((A,(C,G)),(B,K)),L),R),Q))	0	0	1	1	0	0	0	2
(O,((((((A,G),C),B),K),L),(Q,R)))	0	0	1	1	0	0	0	2
(O,(((A,(B,((C,G),K))),L),(Q,R)))	0	0	0	2	0	0	0	2
(O,(((A,((B,K),((C,G),Q))),L),R))	0	0	0	2	0	0	0	2
(O,((((A,(C,G)),K),(B,Q)),(L,R)))	0	0	0	2	0	0	0	2
(O,((((((A,(C,G)),K),B),Q),L),R))	0	0	0	2	0	0	0	2
(O,((A,((B,C),(G,K))),((L,R),Q)))	1	0	1	0	0	0	0	2
(O,(((A,((B,C),(G,K))),L),(Q,R)))	1	0	1	0	0	0	0	2
(O,(((A,((B,(G,K)),C)),L),(Q,R)))	1	0	1	0	0	0	0	2
(O,((((A,(((B,G),K),C)),Q),L),R))	1	0	1	0	0	0	0	2
(O,(((((A,G),C),B),(K,Q)),(L,R)))	1	0	1	0	0	0	0	2
(O,(((((A,G),(B,C)),K),Q),(L,R)))	1	0	1	0	0	0	0	2
(O,(((A,((L,R),Q)),(C,G)),(B,K)))	0	0	2	0	0	0	0	2
(O,((((A,G),C),Q),((B,K),(L,R))))	0	0	2	0	0	0	0	2
(O,((((A,(((B,K),C),G)),Q),L),R))	0	0	2	0	0	0	0	2
(O,((((A,(((B,C),G),K)),Q),L),R))	0	0	2	0	0	0	0	2
(O,(((((A,G),C),B),K),(L,(Q,R))))	0	0	2	0	0	0	0	2
(O,(((((A,G),C),((B,K),L)),Q),R))	0	0	2	0	0	0	0	2
(O,(((((A,((B,G),C)),K),Q),L),R))	0	0	2	0	0	0	0	2
(O,((A,C),(((B,Q),(G,K)),(L,R))))	1	1	0	0	0	0	0	1

(O,((A,C),(((B,(K,Q)),G),(L,R))))	1	1	0	0	0	0	0	1
(O,((A,C),(((B,(K,Q)),(L,R)),G)))	1	1	0	0	0	0	0	1
(O,((A,C),((((B,K),L),(G,Q)),R)))	1	1	0	0	0	0	0	1
(O,((A,C),((((B,(K,Q)),G),L),R)))	1	1	0	0	0	0	0	1
(O,((A,C),(((((B,K),Q),L),R),G)))	1	1	0	0	0	0	0	1
(O,((A,C),(((((B,K),G),Q),R),L)))	1	1	0	0	0	0	0	1
(O,((A,C),(((((B,G),Q),K),L),R)))	1	1	0	0	0	0	0	1
(O,((A,C),(((((B,G),K),Q),L),R)))	1	1	0	0	0	0	0	1
(O,(((A,C),R),(((B,K),G),(L,Q))))	1	1	0	0	0	0	0	1
(O,(((A,C),R),(((B,K),(G,Q)),L)))	1	1	0	0	0	0	0	1
(O,(((A,C),R),((((B,Q),K),G),L)))	1	1	0	0	0	0	0	1
(O,(((A,C),R),((((B,K),G),L),Q)))	1	1	0	0	0	0	0	1
(O,(((A,C),R),((((B,G),K),L),Q)))	1	1	0	0	0	0	0	1
(O,(((A,C),L),((((B,K),G),Q),R)))	1	1	0	0	0	0	0	1
(O,(((A,C),G),((B,K),((L,R),Q))))	1	1	0	0	0	0	0	1
(O,(((A,C),G),(((B,(K,Q)),R),L)))	1	1	0	0	0	0	0	1
(O,(((A,C),G),((((B,K),L),Q),R)))	1	1	0	0	0	0	0	1
(O,(((A,C),((B,Q),(G,K))),(L,R)))	1	1	0	0	0	0	0	1
(O,(((A,C),((B,K),G)),((L,Q),R)))	1	1	0	0	0	0	0	1
(O,(((A,C),(((B,K),G),Q)),(L,R)))	1	1	0	0	0	0	0	1
(O,(((A,C),(((B,K),G),L)),(Q,R)))	1	1	0	0	0	0	0	1
(O,(((A,C),(((B,(K,Q)),G),L)),R))	1	1	0	0	0	0	0	1
(O,((((A,C),R),L),((B,(K,Q)),G)))	1	1	0	0	0	0	0	1
(O,((((A,C),R),(((B,K),Q),G)),L))	1	1	0	0	0	0	0	1
(O,((((A,C),G),R),((B,(K,Q)),L)))	1	1	0	0	0	0	0	1
(O,((((A,C),G),L),((B,K),(Q,R))))	1	1	0	0	0	0	0	1
(O,((((A,C),G),B),(((K,Q),R),L)))	1	1	0	0	0	0	0	1
(O,((((A,C),G),(L,R)),((B,K),Q)))	1	1	0	0	0	0	0	1
(O,((((A,C),G),((K,Q),R)),(B,L)))	1	1	0	0	0	0	0	1
(O,((((A,C),G),((B,Q),K)),(L,R)))	1	1	0	0	0	0	0	1
(O,((((A,C),G),(((B,L),K),Q)),R))	1	1	0	0	0	0	0	1
(O,((((A,C),G),(((B,K),Q),R)),L))	1	1	0	0	0	0	0	1
(O,((((A,C),((B,K),G)),R),(L,Q)))	1	1	0	0	0	0	0	1
(O,((((A,C),(((B,K),Q),G)),L),R))	1	1	0	0	0	0	0	1
(O,((((A,C),(((B,G),K),Q)),R),L))	1	1	0	0	0	0	0	1
(O,(((((A,C),R),L),((B,K),Q)),G))	1	1	0	0	0	0	0	1
(O,(((((A,C),R),G),L),((B,K),Q)))	1	1	0	0	0	0	0	1
(O,(((((A,C),G),R),(B,K)),(L,Q)))	1	1	0	0	0	0	0	1
(O,(((((A,C),G),L),(B,K)),(Q,R)))	1	1	0	0	0	0	0	1
(O,(((((A,C),G),B),R),((K,Q),L)))	1	1	0	0	0	0	0	1
(O,(((((A,C),G),B),(K,Q)),(L,R)))	1	1	0	0	0	0	0	1
(O,((((((A,C),G),B),(L,R)),Q),K))	1	1	0	0	0	0	0	1
(O,((((((A,C),G),B),(K,Q)),L),R))	1	1	0	0	0	0	0	1
(O,((((((A,C),G),(B,K)),Q),R),L))	1	1	0	0	0	0	0	1
(O,(((((((A,C),G),B),K),Q),R),L))	0	0	0	0	0	1	0	1
(O,(A,((B,K),((((C,G),R),L),Q))))	0	0	0	0	0	0	1	1

(O,(A,((B,(K,Q)),(((C,G),R),L))))	0	0	0	0	0	0	1	1
(O,(A,((((B,K),(L,R)),Q),(C,G))))	0	0	0	0	0	0	1	1
(O,((A,Q),((((B,(C,G)),R),L),K)))	0	0	0	0	0	0	1	1
(O,((A,(C,G)),((((B,K),R),Q),L)))	0	0	0	0	0	0	1	1
(O,((A,(B,K)),((C,G),((L,R),Q))))	0	0	0	0	0	0	1	1
(O,((A,(((C,G),R),L)),((B,K),Q)))	0	0	0	0	0	0	1	1
(O,(((A,R),(((B,K),L),Q)),(C,G)))	0	0	0	0	0	0	1	1
(O,(((A,Q),(((C,G),L),R)),(B,K)))	0	0	0	0	0	0	1	1
(O,(((A,L),R),(((B,K),Q),(C,G))))	0	0	0	0	0	0	1	1
(O,(((A,L),(((B,K),(C,G)),Q)),R))	0	0	0	0	0	0	1	1
(O,(((A,(L,R)),(B,(C,G))),(K,Q)))	0	0	0	0	0	0	1	1
(O,(((A,(B,K)),Q),((C,G),(L,R))))	0	0	0	0	0	0	1	1
(O,(((A,(B,K)),((L,Q),R)),(C,G)))	0	0	0	0	0	0	1	1
(O,(((A,(B,K)),((C,G),(L,R))),Q))	0	0	0	0	0	0	1	1
(O,(((A,(B,((K,Q),L))),R),(C,G)))	0	0	0	0	0	0	1	1
(O,(((A,((K,Q),L)),R),(B,(C,G))))	0	0	0	0	0	0	1	1
(O,(((A,((K,Q),L)),B),((C,G),R)))	0	0	0	0	0	0	1	1
(O,(((A,((K,Q),L)),(B,(C,G))),R))	0	0	0	0	0	0	1	1
(O,(((A,((B,K),Q)),R),((C,G),L)))	0	0	0	0	0	0	1	1
(O,(((A,((B,K),Q)),((C,G),R)),L))	0	0	0	0	0	0	1	1
(O,(((A,(((B,K),Q),L)),R),(C,G)))	0	0	0	0	0	0	1	1
(O,((((A,Q),L),K),((B,(C,G)),R)))	0	0	0	0	0	0	1	1
(O,((((A,Q),(K,L)),B),((C,G),R)))	0	0	0	0	0	0	1	1
(O,((((A,L),(C,G)),((B,K),Q)),R))	0	0	0	0	0	0	1	1
(O,((((A,K),B),(C,G)),((L,R),Q)))	0	0	0	0	0	0	1	1
(O,((((A,K),(B,Q)),L),((C,G),R)))	0	0	0	0	0	0	1	1
(O,((((A,(L,R)),(B,K)),Q),(C,G)))	0	0	0	0	0	0	1	1
(O,((((A,(K,R)),(B,Q)),L),(C,G)))	0	0	0	0	0	0	1	1
(O,((((A,(K,Q)),L),B),((C,G),R)))	0	0	0	0	0	0	1	1
(O,((((A,(K,Q)),B),L),((C,G),R)))	0	0	0	0	0	0	1	1
(O,((((A,(K,Q)),(B,(C,G))),R),L))	0	0	0	0	0	0	1	1
(O,((((A,(K,Q)),(B,(C,G))),L),R))	0	0	0	0	0	0	1	1
(O,((((A,(B,K)),Q),R),((C,G),L)))	0	0	0	0	0	0	1	1
(O,((((A,(B,K)),Q),(L,R)),(C,G)))	0	0	0	0	0	0	1	1
(O,((((A,(B,K)),Q),((C,G),R)),L))	0	0	0	0	0	0	1	1
(O,((((A,((K,Q),L)),B),(C,G)),R))	0	0	0	0	0	0	1	1
(O,((((A,((B,K),Q)),R),L),(C,G)))	0	0	0	0	0	0	1	1
(O,((((A,((B,K),Q)),(C,G)),R),L))	0	0	0	0	0	0	1	1
(O,(((((A,Q),K),L),(B,(C,G))),R))	0	0	0	0	0	0	1	1
(O,(((((A,Q),K),B),L),((C,G),R)))	0	0	0	0	0	0	1	1
(O,(((((A,Q),(K,L)),B),R),(C,G)))	0	0	0	0	0	0	1	1
(O,(((((A,L),Q),(B,K)),R),(C,G)))	0	0	0	0	0	0	1	1
(O,(((((A,L),K),Q),B),((C,G),R)))	0	0	0	0	0	0	1	1
(O,(((((A,K),Q),L),(B,(C,G))),R))	0	0	0	0	0	0	1	1
(O,(((((A,K),Q),B),((C,G),R)),L))	0	0	0	0	0	0	1	1
(O,(((((A,B),(K,Q)),L),R),(C,G)))	0	0	0	0	0	0	1	1

(O,(((((A,(K,Q)),B),L),R),(C,G)))	0	0	0	0	0	0	1	1
(O,(((((A,(C,G)),Q),L),(B,K)),R))	0	0	0	0	0	0	1	1
(O,((((((A,Q),K),L),B),R),(C,G)))	0	0	0	0	0	0	1	1
(O,((((((A,Q),K),B),L),R),(C,G)))	0	0	0	0	0	0	1	1
(O,((((((A,L),Q),K),B),(C,G)),R))	0	0	0	0	0	0	1	1
(O,((((((A,K),Q),L),B),R),(C,G)))	0	0	0	0	0	0	1	1
(O,((((((A,K),Q),B),L),R),(C,G)))	0	0	0	0	0	0	1	1
(O,((((((A,K),Q),B),L),(C,G)),R))	0	0	0	0	0	0	1	1
(O,((((((A,K),B),Q),L),R),(C,G)))	0	0	0	0	0	0	1	1
(O,(A,(((B,K),(L,(Q,R))),(C,G))))	0	0	0	0	0	1	0	1
(O,(A,(((B,K),(C,G)),((L,R),Q))))	0	0	0	0	0	1	0	1
(O,((A,R),((((B,K),Q),(C,G)),L)))	0	0	0	0	0	1	0	1
(O,((A,R),((((B,K),(C,G)),Q),L)))	0	0	0	0	0	1	0	1
(O,((A,Q),(((B,K),(C,G)),(L,R))))	0	0	0	0	0	1	0	1
(O,((A,L),(((B,K),(Q,R)),(C,G))))	0	0	0	0	0	1	0	1
(O,((A,(Q,R)),(((B,K),(C,G)),L)))	0	0	0	0	0	1	0	1
(O,((A,(L,R)),(((B,K),(C,G)),Q)))	0	0	0	0	0	1	0	1
(O,((A,(K,((L,R),Q))),(B,(C,G))))	0	0	0	0	0	1	0	1
(O,((A,(C,G)),((B,(K,(Q,R))),L)))	0	0	0	0	0	1	0	1
(O,((A,(C,G)),((((B,K),L),R),Q)))	0	0	0	0	0	1	0	1
(O,((A,(B,K)),(((C,G),(Q,R)),L)))	0	0	0	0	0	1	0	1
(O,((A,(B,(K,(L,(Q,R))))),(C,G)))	0	0	0	0	0	1	0	1
(O,((A,(B,(C,G))),((K,(Q,R)),L)))	0	0	0	0	0	1	0	1
(O,((A,(B,((C,G),K))),(L,(Q,R))))	0	0	0	0	0	1	0	1
(O,((A,(B,((C,G),K))),((L,R),Q)))	0	0	0	0	0	1	0	1
(O,((A,((B,Q),((C,G),K))),(L,R)))	0	0	0	0	0	1	0	1
(O,((A,((B,K),(C,G))),((L,Q),R)))	0	0	0	0	0	1	0	1
(O,((A,((B,(G,K)),C)),((L,R),Q)))	0	0	0	0	0	1	0	1
(O,((A,((B,(C,G)),K)),(L,(Q,R))))	0	0	0	0	0	1	0	1
(O,((A,((B,(C,G)),(K,Q))),(L,R)))	0	0	0	0	0	1	0	1
(O,((A,(((B,Q),K),(C,G))),(L,R)))	0	0	0	0	0	1	0	1
(O,((A,(((B,K),G),C)),(L,(Q,R))))	0	0	0	0	0	1	0	1
(O,((A,(((B,K),C),G)),(L,(Q,R))))	0	0	0	0	0	1	0	1
(O,((A,(((B,K),(L,R)),Q)),(C,G)))	0	0	0	0	0	1	0	1
(O,((A,(((B,K),(C,G)),(Q,R))),L))	0	0	0	0	0	1	0	1
(O,((A,(((B,K),(C,G)),(L,R))),Q))	0	0	0	0	0	1	0	1
(O,((A,(((B,(C,G)),K),(Q,R))),L))	0	0	0	0	0	1	0	1
(O,(((A,Q),R),(((B,K),(C,G)),L)))	0	0	0	0	0	1	0	1
(O,(((A,L),R),(((B,Q),(G,K)),C)))	0	0	0	0	0	1	0	1
(O,(((A,L),Q),(((B,K),(C,G)),R)))	0	0	0	0	0	1	0	1
(O,(((A,K),((B,Q),(C,G))),(L,R)))	0	0	0	0	0	1	0	1
(O,(((A,K),((B,C),G)),((L,R),Q)))	0	0	0	0	0	1	0	1
(O,(((A,G),C),(B,((K,Q),(L,R)))))	0	0	0	0	0	1	0	1
(O,(((A,G),(C,L)),(B,(K,(Q,R)))))	0	0	0	0	0	1	0	1
(O,(((A,G),((L,R),Q)),((B,K),C)))	0	0	0	0	0	1	0	1
(O,(((A,B),(K,(L,(Q,R)))),(C,G)))	0	0	0	0	0	1	0	1

(O,(((A,B),((C,G),K)),(L,(Q,R))))	0	0	0	0	0	1	0	1
(O,(((A,(L,R)),((B,K),(C,G))),Q))	0	0	0	0	0	1	0	1
(O,(((A,(K,Q)),R),(((B,G),C),L)))	0	0	0	0	0	1	0	1
(O,(((A,(K,((L,R),Q))),B),(C,G)))	0	0	0	0	0	1	0	1
(O,(((A,(C,G)),R),(((B,K),Q),L)))	0	0	0	0	0	1	0	1
(O,(((A,(C,G)),K),(B,(L,(Q,R)))))	0	0	0	0	0	1	0	1
(O,(((A,(C,G)),B),(K,((L,Q),R))))	0	0	0	0	0	1	0	1
(O,(((A,(C,G)),B),((K,(Q,R)),L)))	0	0	0	0	0	1	0	1
(O,(((A,(C,G)),(K,L)),((B,Q),R)))	0	0	0	0	0	1	0	1
(O,(((A,(C,G)),((B,K),(Q,R))),L))	0	0	0	0	0	1	0	1
(O,(((A,(C,G)),((B,K),(L,Q))),R))	0	0	0	0	0	1	0	1
(O,(((A,(B,K)),(L,R)),((C,G),Q)))	0	0	0	0	0	1	0	1
(O,(((A,(B,(C,G))),R),((K,Q),L)))	0	0	0	0	0	1	0	1
(O,(((A,(B,(C,(G,K)))),L),(Q,R)))	0	0	0	0	0	1	0	1
(O,(((A,((L,Q),R)),(B,K)),(C,G)))	0	0	0	0	0	1	0	1
(O,(((A,((B,K),L)),(Q,R)),(C,G)))	0	0	0	0	0	1	0	1
(O,(((A,((B,K),(L,(Q,R)))),G),C))	0	0	0	0	0	1	0	1
(O,(((A,((B,C),G)),((L,R),Q)),K))	0	0	0	0	0	1	0	1
(O,(((A,((B,(C,G)),K)),L),(Q,R)))	0	0	0	0	0	1	0	1
(O,(((A,((B,(C,G)),K)),(L,R)),Q))	0	0	0	0	0	1	0	1
(O,(((A,((B,(C,G)),K)),(L,Q)),R))	0	0	0	0	0	1	0	1
(O,(((A,(((B,K),G),C)),L),(Q,R)))	0	0	0	0	0	1	0	1
(O,(((A,(((B,C),G),K)),L),(Q,R)))	0	0	0	0	0	1	0	1
(O,(((A,(((B,(C,G)),K),Q)),L),R))	0	0	0	0	0	1	0	1
(O,((((A,Q),R),(((B,G),C),K)),L))	0	0	0	0	0	1	0	1
(O,((((A,Q),(C,G)),R),((B,K),L)))	0	0	0	0	0	1	0	1
(O,((((A,Q),((B,(C,G)),K)),L),R))	0	0	0	0	0	1	0	1
(O,((((A,L),(C,G)),(B,K)),(Q,R)))	0	0	0	0	0	1	0	1
(O,((((A,L),((B,Q),K)),(C,G)),R))	0	0	0	0	0	1	0	1
(O,((((A,K),(C,G)),(B,Q)),(L,R)))	0	0	0	0	0	1	0	1
(O,((((A,K),((B,C),G)),Q),(L,R)))	0	0	0	0	0	1	0	1
(O,((((A,G),((B,C),K)),Q),(L,R)))	0	0	0	0	0	1	0	1
(O,((((A,B),((C,G),K)),Q),(L,R)))	0	0	0	0	0	1	0	1
(O,((((A,B),((C,G),K)),L),(Q,R)))	0	0	0	0	0	1	0	1
(O,((((A,(Q,R)),(B,K)),L),(C,G)))	0	0	0	0	0	1	0	1
(O,((((A,(C,G)),L),(Q,R)),(B,K)))	0	0	0	0	0	1	0	1
(O,((((A,(C,G)),K),L),(B,(Q,R))))	0	0	0	0	0	1	0	1
(O,((((A,(C,G)),(Q,R)),(B,K)),L))	0	0	0	0	0	1	0	1
(O,((((A,(C,G)),(B,Q)),K),(L,R)))	0	0	0	0	0	1	0	1
(O,((((A,(C,G)),(B,(K,Q))),L),R))	0	0	0	0	0	1	0	1
(O,((((A,(B,G)),(C,K)),Q),(L,R)))	0	0	0	0	0	1	0	1
(O,((((A,(B,C)),(G,Q)),K),(L,R)))	0	0	0	0	0	1	0	1
(O,((((A,(B,(C,G))),L),(Q,R)),K))	0	0	0	0	0	1	0	1
(O,((((A,(B,(C,G))),K),(Q,R)),L))	0	0	0	0	0	1	0	1
(O,((((A,((B,C),G)),R),Q),(K,L)))	0	0	0	0	0	1	0	1
(O,((((A,((B,C),G)),K),(Q,R)),L))	0	0	0	0	0	1	0	1

(O,(((((A,L),(C,G)),R),Q),(B,K)))	0	0	0	0	0	1	0	1
(O,(((((A,L),(C,G)),B),K),(Q,R)))	0	0	0	0	0	1	0	1
(O,(((((A,K),B),(C,G)),Q),(L,R)))	0	0	0	0	0	1	0	1
(O,(((((A,G),C),L),(B,K)),(Q,R)))	0	0	0	0	0	1	0	1
(O,(((((A,G),C),K),B),(L,(Q,R))))	0	0	0	0	0	1	0	1
(O,(((((A,G),C),(L,(Q,R))),K),B))	0	0	0	0	0	1	0	1
(O,(((((A,G),(L,(Q,R))),C),B),K))	0	0	0	0	0	1	0	1
(O,(((((A,C),B),(G,K)),Q),(L,R)))	0	0	0	0	0	1	0	1
(O,(((((A,B),K),(C,G)),Q),(L,R)))	0	0	0	0	0	1	0	1
(O,(((((A,(C,G)),K),B),L),(Q,R)))	0	0	0	0	0	1	0	1
(O,(((((A,(C,G)),K),(B,Q)),R),L))	0	0	0	0	0	1	0	1
(O,(((((A,(C,G)),B),L),(K,Q)),R))	0	0	0	0	0	1	0	1
(O,(((((A,(B,Q)),(C,K)),G),L),R))	0	0	0	0	0	1	0	1
(O,(((((A,(B,G)),(C,K)),Q),L),R))	0	0	0	0	0	1	0	1
(O,(((((A,(B,(C,G))),R),Q),K),L))	0	0	0	0	0	1	0	1
(O,(((((A,((C,G),K)),B),Q),L),R))	0	0	0	0	0	1	0	1
(O,((((((A,K),B),(C,G)),Q),R),L))	0	0	0	0	0	1	0	1
(O,((((((A,(C,G)),B),Q),K),L),R))	0	0	0	0	0	1	0	1
(O,((((((A,(C,G)),B),K),Q),R),L))	0	0	0	0	0	1	0	1
(O,(A,(((B,K),((L,R),Q)),(C,G))))	0	0	0	1	0	0	0	1
(O,((A,(C,G)),((((B,K),Q),L),R)))	0	0	0	1	0	0	0	1
(O,((A,(B,(C,G))),(K,(L,(Q,R)))))	0	0	0	1	0	0	0	1
(O,((A,((((B,K),Q),L),(C,G))),R))	0	0	0	1	0	0	0	1
(O,(((A,G),(B,(C,K))),(L,(Q,R))))	0	0	0	1	0	0	0	1
(O,(((A,(C,G)),B),(K,((L,R),Q))))	0	0	0	1	0	0	0	1
(O,(((A,(C,G)),B),((K,Q),(L,R))))	0	0	0	1	0	0	0	1
(O,(((A,(C,G)),B),(((K,R),Q),L)))	0	0	0	1	0	0	0	1
(O,(((A,(C,G)),(B,Q)),((K,L),R)))	0	0	0	1	0	0	0	1
(O,(((A,((B,C),G)),(K,Q)),(L,R)))	0	0	0	1	0	0	0	1
(O,((((A,K),((B,Q),(C,G))),L),R))	0	0	0	1	0	0	0	1
(O,((((A,C),G),B),(K,((L,R),Q))))	0	0	0	1	0	0	0	1
(O,((((A,(C,G)),(K,Q)),B),(L,R)))	0	0	0	1	0	0	0	1
(O,((((A,(C,G)),((L,R),Q)),B),K))	0	0	0	1	0	0	0	1
(O,((((A,(B,(C,G))),Q),K),(L,R)))	0	0	0	1	0	0	0	1
(O,((((A,(B,((C,K),G))),L),Q),R))	0	0	0	1	0	0	0	1
(O,((((A,((C,G),Q)),(B,K)),L),R))	0	0	0	1	0	0	0	1
(O,((((A,((C,G),K)),(B,Q)),L),R))	0	0	0	1	0	0	0	1
(O,((((A,((B,K),Q)),(C,G)),L),R))	0	0	0	1	0	0	0	1
(O,((((A,((B,G),C)),L),(K,Q)),R))	0	0	0	1	0	0	0	1
(O,(((((A,C),G),B),K),((L,R),Q)))	0	0	0	1	0	0	0	1
(O,(((((A,(C,G)),(B,K)),R),Q),L))	0	0	0	1	0	0	0	1
(O,(((((A,(B,(C,G))),K),L),Q),R))	0	0	0	1	0	0	0	1
(O,((((((A,G),C),B),Q),K),(L,R)))	0	0	0	1	0	0	0	1
(O,((((((A,G),C),B),K),Q),(L,R)))	0	0	0	1	0	0	0	1
(O,((((((A,G),C),B),K),(Q,R)),L))	0	0	0	1	0	0	0	1
(O,((((((A,B),(C,G)),K),Q),L),R))	0	0	0	1	0	0	0	1

(O,((((((A,(C,G)),K),B),Q),R),L))	0	0	0	1	0	0	0	1
(O,(((((((A,G),C),B),Q),K),L),R))	0	0	0	1	0	0	0	1
(O,((A,G),((((B,K),Q),(L,R)),C)))	0	0	1	0	0	0	0	1
(O,((A,(L,R)),((B,K),((C,G),Q))))	0	0	1	0	0	0	0	1
(O,((A,(C,(G,K))),((B,Q),(L,R))))	0	0	1	0	0	0	0	1
(O,((A,((B,G),(C,K))),((L,Q),R)))	0	0	1	0	0	0	0	1
(O,((A,(((B,K),L),(Q,R))),(C,G)))	0	0	1	0	0	0	0	1
(O,((A,(((B,G),C),K)),((L,Q),R)))	0	0	1	0	0	0	0	1
(O,((A,(((B,C),G),K)),((L,R),Q)))	0	0	1	0	0	0	0	1
(O,(((A,Q),((B,C),(G,K))),(L,R)))	0	0	1	0	0	0	0	1
(O,(((A,Q),((B,(G,K)),C)),(L,R)))	0	0	1	0	0	0	0	1
(O,(((A,L),((B,(C,G)),K)),(Q,R)))	0	0	1	0	0	0	0	1
(O,(((A,G),C),(((B,K),Q),(L,R))))	0	0	1	0	0	0	0	1
(O,(((A,G),C),(((B,K),(L,R)),Q)))	0	0	1	0	0	0	0	1
(O,(((A,G),((B,K),C)),((L,R),Q)))	0	0	1	0	0	0	0	1
(O,(((A,G),(((B,K),(L,R)),Q)),C))	0	0	1	0	0	0	0	1
(O,(((A,(C,G)),Q),(B,((K,R),L))))	0	0	1	0	0	0	0	1
(O,(((A,(C,G)),Q),(((B,K),R),L)))	0	0	1	0	0	0	0	1
(O,(((A,(C,G)),L),(((B,K),R),Q)))	0	0	1	0	0	0	0	1
(O,(((A,(C,G)),B),(((K,R),L),Q)))	0	0	1	0	0	0	0	1
(O,(((A,(C,G)),B),(((K,Q),R),L)))	0	0	1	0	0	0	0	1
(O,(((A,(C,G)),(L,(Q,R))),(B,K)))	0	0	1	0	0	0	0	1
(O,(((A,(C,G)),((B,K),(L,R))),Q))	0	0	1	0	0	0	0	1
(O,(((A,(B,(C,G))),(L,R)),(K,Q)))	0	0	1	0	0	0	0	1
(O,(((A,(B,(C,G))),((L,R),Q)),K))	0	0	1	0	0	0	0	1
(O,(((A,((B,K),(C,G))),(L,Q)),R))	0	0	1	0	0	0	0	1
(O,(((A,((B,G),C)),K),(L,(Q,R))))	0	0	1	0	0	0	0	1
(O,(((A,((B,C),G)),K),(L,(Q,R))))	0	0	1	0	0	0	0	1
(O,(((A,((B,(G,K)),C)),(Q,R)),L))	0	0	1	0	0	0	0	1
(O,(((A,(((B,K),C),G)),L),(Q,R)))	0	0	1	0	0	0	0	1
(O,((((A,Q),(L,R)),(B,K)),(C,G)))	0	0	1	0	0	0	0	1
(O,((((A,L),((B,K),(C,G))),Q),R))	0	0	1	0	0	0	0	1
(O,((((A,G),C),L),((B,K),(Q,R))))	0	0	1	0	0	0	0	1
(O,((((A,G),C),B),(K,(L,(Q,R)))))	0	0	1	0	0	0	0	1
(O,((((A,G),C),(B,(K,Q))),(L,R)))	0	0	1	0	0	0	0	1
(O,((((A,G),C),(((B,K),Q),R)),L))	0	0	1	0	0	0	0	1
(O,((((A,C),G),B),(K,(L,(Q,R)))))	0	0	1	0	0	0	0	1
(O,((((A,C),(B,G)),(K,Q)),(L,R)))	0	0	1	0	0	0	0	1
(O,((((A,C),((B,G),K)),Q),(L,R)))	0	0	1	0	0	0	0	1
(O,((((A,B),(C,G)),K),(L,(Q,R))))	0	0	1	0	0	0	0	1
(O,((((A,B),(C,(G,K))),Q),(L,R)))	0	0	1	0	0	0	0	1
(O,((((A,(C,G)),L),((B,K),Q)),R))	0	0	1	0	0	0	0	1
(O,((((A,(C,G)),B),L),(K,(Q,R))))	0	0	1	0	0	0	0	1
(O,((((A,(C,G)),B),K),((L,Q),R)))	0	0	1	0	0	0	0	1
(O,((((A,(C,G)),B),(Q,R)),(K,L)))	0	0	1	0	0	0	0	1
(O,((((A,(C,G)),(L,Q)),(B,K)),R))	0	0	1	0	0	0	0	1

(O,((((A,(C,G)),((B,K),L)),Q),R))	0	0	1	0	0	0	0	1
(O,((((A,(C,(G,K))),(B,Q)),L),R))	0	0	1	0	0	0	0	1
(O,((((A,(B,C)),(G,K)),Q),(L,R)))	0	0	1	0	0	0	0	1
(O,((((A,((B,K),G)),C),L),(Q,R)))	0	0	1	0	0	0	0	1
(O,((((A,((B,(G,K)),C)),Q),L),R))	0	0	1	0	0	0	0	1
(O,((((A,(((B,K),G),C)),Q),R),L))	0	0	1	0	0	0	0	1
(O,((((A,(((B,K),G),C)),L),R),Q))	0	0	1	0	0	0	0	1
(O,(((((A,K),G),(B,C)),Q),(L,R)))	0	0	1	0	0	0	0	1
(O,(((((A,K),((B,G),C)),Q),L),R))	0	0	1	0	0	0	0	1
(O,(((((A,K),((B,C),G)),L),Q),R))	0	0	1	0	0	0	0	1
(O,(((((A,G),C),B),Q),(K,(L,R))))	0	0	1	0	0	0	0	1
(O,(((((A,G),C),(B,K)),(L,R)),Q))	0	0	1	0	0	0	0	1
(O,(((((A,C),G),K),(B,Q)),(L,R)))	0	0	1	0	0	0	0	1
(O,(((((A,C),((B,K),G)),Q),L),R))	0	0	1	0	0	0	0	1
(O,(((((A,(G,K)),C),B),Q),(L,R)))	0	0	1	0	0	0	0	1
(O,(((((A,(C,G)),K),(B,Q)),L),R))	0	0	1	0	0	0	0	1
(O,(((((A,(C,G)),B),Q),(K,R)),L))	0	0	1	0	0	0	0	1
(O,(((((A,(B,(C,G))),Q),K),L),R))	0	0	1	0	0	0	0	1
(O,(((((A,(B,(C,G))),K),Q),R),L))	0	0	1	0	0	0	0	1
(O,(((((A,((C,G),K)),B),L),Q),R))	0	0	1	0	0	0	0	1
(O,((((((A,K),B),G),C),Q),(L,R)))	0	0	1	0	0	0	0	1
(O,((((((A,G),C),(B,K)),L),Q),R))	0	0	1	0	0	0	0	1
(O,((((((A,C),(B,K)),G),Q),L),R))	0	0	1	0	0	0	0	1
(O,((((((A,(G,K)),C),B),Q),L),R))	0	0	1	0	0	0	0	1
(O,((((((A,(C,G)),B),K),L),R),Q))	0	0	1	0	0	0	0	1
(O,((((((A,(B,C)),G),K),Q),R),L))	0	0	1	0	0	0	0	1
(O,(A,((B,(C,G)),((K,Q),(L,R)))))	1	0	0	0	0	0	0	1
(O,(A,(((((B,K),Q),(C,G)),R),L)))	1	0	0	0	0	0	0	1
(O,((A,Q),((B,(C,G)),(K,(L,R)))))	1	0	0	0	0	0	0	1
(O,((A,(L,R)),((B,(C,G)),(K,Q))))	1	0	0	0	0	0	0	1
(O,((A,(C,G)),((B,K),((L,Q),R))))	1	0	0	0	0	0	0	1
(O,((A,((B,Q),(C,G))),((K,R),L)))	1	0	0	0	0	0	0	1
(O,((A,((B,G),(C,K))),(L,(Q,R))))	1	0	0	0	0	0	0	1
(O,((A,((B,((C,G),K)),Q)),(L,R)))	1	0	0	0	0	0	0	1
(O,((A,(((B,G),C),K)),((L,R),Q)))	1	0	0	0	0	0	0	1
(O,(((A,Q),((B,R),(K,L))),(C,G)))	1	0	0	0	0	0	0	1
(O,(((A,K),(B,(C,G))),((L,R),Q)))	1	0	0	0	0	0	0	1
(O,(((A,G),C),((((B,K),Q),R),L)))	1	0	0	0	0	0	0	1
(O,(((A,(L,R)),(K,Q)),(B,(C,G))))	1	0	0	0	0	0	0	1
(O,(((A,((B,K),(C,G))),R),(L,Q)))	1	0	0	0	0	0	0	1
(O,(((A,((B,K),((L,R),Q))),G),C))	1	0	0	0	0	0	0	1
(O,(((A,(((B,K),Q),R)),(C,G)),L))	1	0	0	0	0	0	0	1
(O,((((A,Q),R),((B,K),(C,G))),L))	1	0	0	0	0	0	0	1
(O,((((A,Q),(C,G)),((B,K),L)),R))	1	0	0	0	0	0	0	1
(O,((((A,K),(B,(C,G))),(L,R)),Q))	1	0	0	0	0	0	0	1
(O,((((A,G),C),((B,Q),K)),(L,R)))	1	0	0	0	0	0	0	1

(O,((((A,G),(C,Q)),(B,K)),(L,R)))	1	0	0	0	0	0	0	1
(O,((((A,(L,R)),Q),(B,K)),(C,G)))	1	0	0	0	0	0	0	1
(O,((((A,(C,G)),L),(K,Q)),(B,R)))	1	0	0	0	0	0	0	1
(O,((((A,(C,G)),B),K),((L,R),Q)))	1	0	0	0	0	0	0	1
(O,((((A,(C,G)),B),(K,R)),(L,Q)))	1	0	0	0	0	0	0	1
(O,((((A,(C,G)),(L,(Q,R))),B),K))	1	0	0	0	0	0	0	1
(O,((((A,(C,G)),((B,K),L)),R),Q))	1	0	0	0	0	0	0	1
(O,((((A,(B,(C,G))),(K,Q)),R),L))	1	0	0	0	0	0	0	1
(O,((((A,(B,(C,(G,K)))),Q),L),R))	1	0	0	0	0	0	0	1
(O,((((A,(B,((C,K),G))),Q),L),R))	1	0	0	0	0	0	0	1
(O,(((((A,K),(B,(C,G))),Q),R),L))	1	0	0	0	0	0	0	1
(O,(((((A,G),(B,K)),C),Q),(L,R)))	1	0	0	0	0	0	0	1
(O,(((((A,C),(B,K)),G),(L,R)),Q))	1	0	0	0	0	0	0	1
(O,(((((A,(C,G)),B),(L,R)),Q),K))	1	0	0	0	0	0	0	1
(O,(((((A,(C,G)),B),(K,Q)),R),L))	1	0	0	0	0	0	0	1
(O,(((((A,(C,G)),(K,Q)),B),L),R))	1	0	0	0	0	0	0	1
(O,(((((A,((B,K),C)),G),Q),L),R))	1	0	0	0	0	0	0	1
(O,((((((A,G),B),C),K),Q),(L,R)))	1	0	0	0	0	0	0	1

Table S5. Pairwise distance and million years (Ma) between *An. gambiae* - *An. coluzzii* clade (GC) and *An. fontenillei* - *An. bwambae* clade (FB). Results showed for each window of the X chromosome used in the analysis; trees where *An. fontenillei* and *An. bwambae* were the closest. We assumed a substitution rate of $1.1x10^{-9}$ per site, per generation, and 10 generation per year (Tamura et al. 2004).

chr	window	'airwise distance (FB	airwise distance (G(	Ma (FB)	Ma (GC)
X	150000	0.0261	0.0035	1.1850	0.1591
X	200000	0.0192	0.0049	0.8710	0.2211
X	300000	0.0103	0.0049	0.4694	0.2249
X	350000	0.0104	0.0042	0.4719	0.1925
X	400000	0.0129	0.0049	0.5863	0.2235
X	450000	0.0104	0.0062	0.4727	0.2796
X	500000	0.0188	0.0069	0.8535	0.3130
X	550000	0.0166	0.0076	0.7549	0.3460
X	700000	0.0180	0.0079	0.8191	0.3582
X	750000	0.0173	0.0069	0.7848	0.3146
X	800000	0.0118	0.0078	0.5366	0.3566
X	850000	0.0183	0.0071	0.8319	0.3231
X	1000000	0.0136	0.0066	0.6169	0.3011
X	1100000	0.0149	0.0093	0.6760	0.4211
X	1250000	0.0181	0.0062	0.8215	0.2833
X	1300000	0.0129	0.0028	0.5844	0.1290
X	1350000	0.0139	0.0066	0.6307	0.3021
X	1450000	0.0152	0.0067	0.6914	0.3027
X	1600000	0.0153	0.0065	0.6943	0.2947
X	1800000	0.0137	0.0074	0.6214	0.3374
X	1850000	0.0188	0.0090	0.8554	0.4073
X	1950000	0.0180	0.0082	0.8172	0.3709
X	2000000	0.0201	0.0098	0.9146	0.4461
X	2050000	0.0153	0.0077	0.6976	0.3493
X	2100000	0.0103	0.0066	0.4674	0.3003
X	2150000	0.0130	0.0057	0.5900	0.2572
X	2200000	0.0081	0.0064	0.3693	0.2896
X	2350000	0.0180	0.0100	0.8169	0.4563
X	2450000	0.0177	0.0103	0.8027	0.4672
X	2500000	0.0138	0.0101	0.6295	0.4593
X	2550000	0.0478	0.0100	2.1741	0.4538
X	2600000	0.0227	0.0091	1.0319	0.4141
X	2650000	0.0229	0.0092	1.0406	0.4165
X	2700000	0.0175	0.0096	0.7974	0.4357
X	2750000	0.0179	0.0633	0.8137	2.8777
X	2800000	0.0158	0.0066	0.7183	0.2980
X	2850000	0.0135	0.0073	0.6138	0.3323
X	2900000	0.0188	0.0080	0.8527	0.3651
X	2950000	0.0202	0.0054	0.9191	0.2471
X	3000000	0.0133	0.0064	0.6056	0.2914

X	3050000	0.0094	0.0076	0.4256	0.3467
X	3100000	0.0097	0.0046	0.4412	0.2104
X	3150000	0.0200	0.0098	0.9071	0.4455
X	3400000	0.0132	0.0074	0.5983	0.3368
X	3450000	0.0317	0.0113	1.4389	0.5117
X	3500000	0.0115	0.0069	0.5229	0.3155
X	3550000	0.0248	0.0063	1.1276	0.2878
X	3600000	0.0736	0.0098	3.3432	0.4443
X	3750000	0.0269	0.0125	1.2232	0.5693
X	3800000	0.0236	0.0120	1.0710	0.5461
X	3850000	0.0267	0.0098	1.2141	0.4464
X	3900000	0.0322	0.0091	1.4615	0.4142
X	3950000	0.0164	0.0094	0.7451	0.4279
X	4100000	0.0174	0.0060	0.7902	0.2726
X	4200000	0.0172	0.0082	0.7839	0.3706
X	4250000	0.0227	0.0107	1.0324	0.4874
X	4300000	0.0191	0.0094	0.8670	0.4266
X	4350000	0.0198	0.0090	0.9000	0.4078
X	4400000	0.0142	0.0076	0.6452	0.3461
X	4600000	0.0162	0.0054	0.7382	0.2452
X	4650000	0.0154	0.0085	0.6982	0.3867
X	4950000	0.0125	0.0083	0.5668	0.3790
X	5000000	0.0154	0.0073	0.7016	0.3329
X	5050000	0.0161	0.0052	0.7297	0.2343
X	5150000	0.0442	0.0086	2.0093	0.3915
X	5400000	0.0192	0.0059	0.8745	0.2698
X	5450000	0.0158	0.0045	0.7166	0.2058
X	5700000	0.0070	0.0059	0.3202	0.2676
X	5750000	0.0103	0.0085	0.4690	0.3849
X	5800000	0.0118	0.0054	0.5371	0.2470
X	5900000	0.0171	0.0045	0.7785	0.2061
X	6000000	0.0180	0.0090	0.8162	0.4082
X	6100000	0.0257	0.0096	1.1691	0.4363
X	6450000	0.0408	0.0093	1.8531	0.4231
X	6500000	0.0296	0.0123	1.3461	0.5587
X	6650000	0.0246	0.0120	1.1191	0.5432
X	6750000	0.0197	0.0097	0.8938	0.4414
X	6800000	0.0087	0.0069	0.3947	0.3135
X	6900000	0.0082	0.0082	0.3741	0.3741
X	7050000	0.0295	0.0109	1.3414	0.4936
X	7100000	0.0115	0.0075	0.5206	0.3395
X	7150000	0.0087	0.0055	0.3937	0.2509
X	7200000	0.0139	0.0051	0.6305	0.2331
X	7250000	0.0140	0.0066	0.6362	0.2978
X	7300000	0.0111	0.0059	0.5052	0.2703
X	7350000	0.0142	0.0060	0.6450	0.2743

X	7400000	0.0084	0.0052	0.3821	0.2380
X	7500000	0.0071	0.0067	0.3235	0.3047
X	7700000	0.0085	0.0068	0.3884	0.3077
X	7850000	0.0087	0.0088	0.3968	0.4013
X	8050000	0.0115	0.0049	0.5212	0.2207
X	8100000	0.0154	0.0085	0.6986	0.3886
X	8400000	0.0234	0.0098	1.0631	0.4465
X	8450000	0.0098	0.0073	0.4440	0.3333
X	8550000	0.0169	0.0080	0.7689	0.3655
X	8750000	0.0112	0.0079	0.5111	0.3573
X	8800000	0.0168	0.0037	0.7639	0.1704
X	8850000	0.0134	0.0097	0.6090	0.4427
X	8900000	0.0191	0.0088	0.8686	0.4007
X	9050000	0.0304	0.0105	1.3826	0.4768
X	9150000	0.0290	0.0087	1.3185	0.3966
X	9300000	0.0226	0.0081	1.0285	0.3696
X	9350000	0.0242	0.0119	1.1014	0.5403
X	9500000	0.0129	0.0049	0.5844	0.2228
X	9550000	0.0215	0.0072	0.9765	0.3276
X	9600000	0.0149	0.0058	0.6793	0.2647
X	9650000	0.0189	0.0098	0.8597	0.4464
X	9700000	0.0125	0.0103	0.5701	0.4694
X	9750000	0.0187	0.0107	0.8494	0.4876
X	9800000	0.0214	0.0086	0.9715	0.3898
X	9850000	0.0129	0.0078	0.5849	0.3543
X	9900000	0.0129	0.0078	0.5849	0.3543
X	9950000	0.0127	0.0072	0.5752	0.3291
X	10100000	0.0207	0.0077	0.9392	0.3477
X	10150000	0.0080	0.0050	0.3622	0.2277
X	10200000	0.0185	0.0074	0.8402	0.3379
X	10550000	0.0087	0.0092	0.3938	0.4162
X	10650000	0.0252	0.0106	1.1467	0.4802
X	10800000	0.0142	0.0114	0.6450	0.5192
X	10850000	0.0213	0.0102	0.9678	0.4658
X	10950000	0.0215	0.0099	0.9769	0.4497
X	11100000	0.0214	0.0069	0.9722	0.3116
X	11150000	0.0225	0.0072	1.0238	0.3270
X	11200000	0.0464	0.0083	2.1085	0.3761
X	11250000	0.0237	0.0067	1.0755	0.3059
X	11350000	0.0115	0.0068	0.5214	0.3082
X	11400000	0.0085	0.0044	0.3886	0.2000
X	11450000	0.0183	0.0058	0.8311	0.2632
X	11600000	0.0159	0.0063	0.7206	0.2845
X	11700000	0.0485	0.0052	2.2051	0.2361
X	11750000	0.0063	0.0048	0.2870	0.2159
X	11800000	0.0067	0.0055	0.3051	0.2480

X	11850000	0.0073	0.0026	0.3297	0.1166
X	11900000	0.0127	0.0067	0.5774	0.3064
X	11950000	0.0207	0.0064	0.9413	0.2913
X	12000000	0.0135	0.0072	0.6156	0.3265
X	12100000	0.0140	0.0073	0.6367	0.3321
X	12150000	0.0104	0.0030	0.4727	0.1362
X	12200000	0.0131	0.0050	0.5967	0.2264
X	12250000	0.0223	0.0073	1.0117	0.3331
X	12300000	0.0119	0.0058	0.5423	0.2654
X	12350000	0.0203	0.0076	0.9221	0.3464
X	12500000	0.0175	0.0106	0.7955	0.4818
X	12550000	0.0119	0.0058	0.5425	0.2631
X	12600000	0.0175	0.0055	0.7942	0.2497
X	12650000	0.0117	0.0085	0.5313	0.3863
X	12700000	0.0185	0.0067	0.8406	0.3028
X	12800000	0.0079	0.0049	0.3603	0.2244
X	12900000	0.0210	0.0101	0.9531	0.4594
X	13050000	0.0168	0.0106	0.7622	0.4821
X	13100000	0.0254	0.0101	1.1563	0.4575
X	13150000	0.0112	0.0058	0.5113	0.2640
X	13200000	0.0100	0.0046	0.4531	0.2069
X	13250000	0.0111	0.0061	0.5062	0.2789
X	13300000	0.0126	0.0101	0.5748	0.4582
X	13350000	0.0096	0.0085	0.4385	0.3855
X	13400000	0.0060	0.0079	0.2738	0.3572
X	13450000	0.0042	0.0063	0.1928	0.2862
X	13550000	0.0110	0.0055	0.4987	0.2499
X	13600000	0.0146	0.0103	0.6648	0.4698
X	13650000	0.0165	0.0079	0.7517	0.3595
X	13700000	0.0100	0.0073	0.4542	0.3335
X	13750000	0.0073	0.0043	0.3332	0.1962
X	13800000	0.0102	0.0050	0.4624	0.2258
X	13850000	0.0082	0.0052	0.3747	0.2364
X	13900000	0.0041	0.0056	0.1870	0.2529
X	13950000	0.0030	0.0047	0.1370	0.2140
X	14000000	0.0100	0.0061	0.4562	0.2779
X	14050000	0.0169	0.0064	0.7680	0.2903
X	14200000	0.0144	0.0096	0.6537	0.4365
X	14300000	0.0096	0.0063	0.4348	0.2848
X	14350000	0.0160	0.0057	0.7285	0.2587
X	14400000	0.0135	0.0046	0.6151	0.2080
X	14550000	0.0203	0.0055	0.9237	0.2505
X	14700000	0.0278	0.0100	1.2628	0.4526
X	14750000	0.0307	0.0103	1.3965	0.4697
X	14850000	0.0081	0.0055	0.3688	0.2514
X	15300000	0.0022	0.0060	0.0990	0.2715

X	15350000	0.0053	0.0035	0.2399	0.1601
X	15400000	0.0045	0.0051	0.2059	0.2304
X	15450000	0.0037	0.0067	0.1676	0.3067
X	15500000	0.0052	0.0050	0.2385	0.2258
X	15550000	0.0035	0.0046	0.1576	0.2103
X	15600000	0.0078	0.0078	0.3548	0.3536
X	15650000	0.0024	0.0055	0.1076	0.2515
X	15700000	0.0044	0.0048	0.2005	0.2184
X	15750000	0.0096	0.0048	0.4379	0.2176
X	15800000	0.0088	0.0072	0.3989	0.3295
X	15850000	0.0060	0.0050	0.2726	0.2260
X	15950000	0.0085	0.0078	0.3874	0.3550
X	16000000	0.0080	0.0087	0.3646	0.3947
X	16050000	0.0078	0.0064	0.3539	0.2905
X	16100000	0.0093	0.0100	0.4223	0.4548
X	16250000	0.0061	0.0053	0.2774	0.2426
X	16300000	0.0063	0.0074	0.2846	0.3355
X	16350000	0.0068	0.0055	0.3074	0.2504
X	16400000	0.0047	0.0079	0.2150	0.3610
X	16450000	0.0059	0.0060	0.2662	0.2741
X	16500000	0.0071	0.0064	0.3249	0.2918
X	16550000	0.0055	0.0070	0.2516	0.3201
X	16600000	0.0063	0.0079	0.2849	0.3607
X	16650000	0.0059	0.0064	0.2692	0.2890
X	16700000	0.0050	0.0044	0.2284	0.1989
X	16750000	0.0060	0.0034	0.2717	0.1535
X	16800000	0.0066	0.0061	0.3021	0.2762
X	16850000	0.0055	0.0041	0.2494	0.1848
X	16900000	0.0067	0.0057	0.3053	0.2608
X	16950000	0.0078	0.0057	0.3563	0.2585
X	17000000	0.0070	0.0066	0.3182	0.2983
X	17050000	0.0083	0.0050	0.3754	0.2267
X	17100000	0.0081	0.0065	0.3693	0.2975
X	17150000	0.0039	0.0064	0.1770	0.2928
X	17200000	0.0049	0.0051	0.2224	0.2321
X	17250000	0.0051	0.0094	0.2299	0.4289
X	17400000	0.0044	0.0037	0.1996	0.1668
X	17500000	0.0032	0.0034	0.1438	0.1532
X	17550000	0.0046	0.0063	0.2075	0.2861
X	17600000	0.0073	0.0067	0.3309	0.3033
X	17650000	0.0059	0.0069	0.2667	0.3147
X	17700000	0.0055	0.0060	0.2480	0.2711
X	17750000	0.0147	0.0079	0.6683	0.3587
X	17800000	0.0099	0.0057	0.4504	0.2613
X	17850000	0.0092	0.0074	0.4202	0.3380
X	17900000	0.0037	0.0074	0.1669	0.3348

X	17950000	0.0063	0.0098	0.2882	0.4436
X	18000000	0.0070	0.0065	0.3196	0.2961
X	18050000	0.0084	0.0069	0.3836	0.3156
X	18100000	0.0030	0.0065	0.1384	0.2961
X	18150000	0.0030	0.0065	0.1384	0.2961
X	18200000	0.0068	0.0076	0.3106	0.3476
X	18250000	0.0068	0.0085	0.3092	0.3845
X	18300000	0.0092	0.0070	0.4188	0.3193
X	18350000	0.0063	0.0052	0.2877	0.2370
X	18400000	0.0057	0.0082	0.2607	0.3734
X	18450000	0.0065	0.0061	0.2951	0.2777
X	18500000	0.0060	0.0062	0.2726	0.2839
X	18550000	0.0112	0.0066	0.5080	0.3000
X	18600000	0.0055	0.0044	0.2520	0.1993
X	18650000	0.0056	0.0074	0.2553	0.3349
X	18750000	0.0016	0.0058	0.0746	0.2617
X	18800000	0.0028	0.0054	0.1292	0.2467
X	18850000	0.0029	0.0063	0.1310	0.2886
X	18900000	0.0052	0.0067	0.2374	0.3027
X	18950000	0.0072	0.0045	0.3280	0.2060
X	19000000	0.0034	0.0077	0.1538	0.3492
X	19100000	0.0053	0.0084	0.2412	0.3803
X	19150000	0.0071	0.0075	0.3237	0.3417
X	19200000	0.0049	0.0056	0.2216	0.2540
X	19300000	0.0045	0.0061	0.2062	0.2753
X	19350000	0.0072	0.0056	0.3279	0.2547
X	19400000	0.0041	0.0049	0.1867	0.2213
X	19450000	0.0145	0.0060	0.6584	0.2728
X	19500000	0.0047	0.0059	0.2135	0.2682
X	19550000	0.0121	0.0054	0.5494	0.2436
X	19600000	0.0051	0.0048	0.2333	0.2166
X	19650000	0.0035	0.0072	0.1583	0.3262
X	19700000	0.0044	0.0077	0.2018	0.3488
X	19750000	0.0033	0.0070	0.1503	0.3161
X	19800000	0.0019	0.0051	0.0856	0.2299
X	19850000	0.0014	0.0070	0.0648	0.3175
X	19900000	0.0005	0.0053	0.0213	0.2427
X	19950000	0.0005	0.0053	0.0213	0.2427
X	20150000	0.0008	0.0063	0.0371	0.2882
X	20200000	0.0008	0.0047	0.0357	0.2130

Table S6. Number of windows (#) and proportion (%) that represent each phylogenetic tree topology. Results are shown for all the genome, for the autosomes and for each chromosome arm. Analyzed column is estimated without taking into account the not analyzed (NA) windows. F: *An. fontenillei*, B: *An. bwambae*, Q: *An. quadriannulatus*, A: *An. arabiensis*, G: *An. gambiae*, C: *An. coluzzii*, L: *An. melas*.

	**Genome**			**X**			**3L**			**3R**			**2L**			**2R**			AUTOSOMES		
**Topology**	windows	%	nalyzed %	windows	%	nalyzed %	windows	%	nalyzed %	windows	%	nalyzed %	windows	%	nalyzed %	windows	%	nalyzed %	windows	%	Analyzed %
(F,B),Q	786	17.39	18.99	111	27.48	34.80	54	6.43	6.97	125	11.77	12.94	394	39.92	42.78	102	8.32	8.82	675	16.40	17.67
(F,B),A	151	3.34	3.65	99	24.50	31.03	5	0.60	0.65	15	1.41	1.55	26	2.63	2.82	6	0.49	0.52	52	1.26	1.36
(F,B)(A,Q)	38	0.84	0.92	27	6.68	8.46	1	0.12	0.13	9	0.85	0.93	0	0.00	0.00	1	0.08	0.09	11	0.27	0.29
(F,B)(A,(G,C)	1142	25.27	27.60	7	1.73	2.19	145	17.26	18.71	299	28.15	30.95	158	16.01	17.16	533	43.47	46.07	1135	27.58	29.72
(F,B)(G,C)	402	8.90	9.71	10	2.48	3.13	25	2.98	3.23	187	17.61	19.36	80	8.11	8.69	100	8.16	8.64	392	9.53	10.26
(F,B).L	410	9.07	9.91	0	0.00	0.00	378	45.00	48.77	18	1.69	1.86	0	0.00	0.00	14	1.14	1.21	410	9.96	10.74
(F,B)-other	546	12.08	13.19	10	2.48	3.13	63	7.50	8.13	104	9.79	10.77	135	13.68	14.66	234	19.09	20.22	536	13.03	14.04
(F…B)	663	14.67	16.02	55	13.61	17.24	104	12.38	13.42	209	19.68	21.64	128	12.97	13.90	167	13.62	14.43	608	14.78	15.92
NA	381	8.43	0.00	85	21.04	0.00	65	7.74	0.00	96	9.04	0.00	66	6.69	0.00	69	5.63	0.00	296	7.19	0.00

Table S7. Candidate gene analysis. 42 mutations in 14 genes related to insecticide resistance and 5 mutations in 1 gene related to immunity and infection resistance for each *An. fontenillie* individuals (nf2, nf3, nf4 and nf5).

ID	GENE	vectorBase	MUTATION	ENOME POSITIO	Ref allele	Resistant allele	nf2	nf3	nf4	nf5	Susceptible/Resistant
1	VGSC	AGAP004707	L995F	2L:2422651	T		37 reads T	69 reads T	53 reads T	47 reads T	Susceptible
1	VGSC	AGAP004707	L995S	2L:2422652	A		37 reads A	70 reads A	53 reads A	48 reads A	Susceptible
1	VGSC	AGAP004707	N1597Y	2L:2429745	A		54 reads A	51 reads A	43 reads A	37 reads A	Susceptible
1	VGSC	AGAP004707	P1874S	2L:430880	C		43 reads C	50 reads C	49 reads C	35 reads C	Susceptible
2	GSTE2	AGAP009194	I114T	3R:28598166	Reverse T		40 reads A	47 reads A	38 reads A	33 reads A	Susceptible
2	GSTE2	AGAP009194	L36P	3R:28598474	Reverse T	Reverse C	38 reads A	37 reads A	42 reads A	50 reads A	Susceptible
3	GSTE8	AGAP00919<br>0	D211E	3R:28591702	Reverse C	Reverse A/G	35 reads G	42 reads G	48 reads G	23 reads G	Susceptible
3	GSTE8	AGAP00919<br>0	E197K	3R:28591746	Reverse G	Reverse A	33 reads C	47 reads C	40 reads C	27 reads C	Susceptible
3	GSTE8	AGAP00919<br>0	P187Q	3R:28591775	Reverse C	Reverse A	39 reads G	42 reads G	31 reads G /	23 reads G	Susceptible / 1 read resistance
3	GSTE8	AGAP00919<br>0	T90K	3R:28592132	Reverse C	Reverse A	26 reads G	30 reads G	48 reads G	30 reads G	Susceptible
3	GSTE8	AGAP00919<br>0	A6V	3R:28592453	Reverse C	Reverse T	22 reads G	23 reads G	34 reads G	29 reads G	Susceptible
4	GSTE6	AGAP009191	E89D	3R:28594071	A	T/C	29 reads C	35 reads C	32 reads C	24 reads C	Resistant
4	GSTE6	AGAP009191	A126V	3R:28594181	C	T	32 reads C	27 reads C /	38 reads C	49 reads C	Susceptible
5	GSTE5	AGAP009192	S158T	3R:28595230	Reverse T	Reverse A	36 reads A	26 reads A	44 reads A	30 reads A	Susceptible
5	GSTE5	AGAP009192	E143D	3R:28595273	Reverse G	Reverse T/C	44 reads C	35 reads C	48 reads C	33 reads C	Susceptible
5	GSTE5	AGAP009192	G109S	3R:28595449	Reverse G	Reverse A	40 reads C	30 reads C	31 reads C	29 reads C	Susceptible
6	GSTE4	AGAP009193	K190E	3R:28596184	Reverse A	Reverse G	19 reads T / 18	27 reads T	34 reads T	14 reads T / 16 reads C	2 polymorphic individuals / 2 susceptible individuals
7	GSTE1		*								
8	GSTE7	AGAP009196	S185I	3R:28601180	T	A	35 reads T	37 reads T	39 reads T /	45 reads T	Susceptible
8	GSTE7	AGAP009196	S185I	3R:28601181	C	T	34 reads C / 1	37 reads C	40 reads C	44 reads C	Susceptible
9	GSTE3	AGAP009197	N73I	3R:28601902	Reverse A	Reverse T	28 reads A	25 reads A	37 reads A	30 reads A	Resistant
#	CYP6P3	AGAP002865	M219L	2R:28492487	Reverse A	Reverse C	5 reads T	1 read T	3 reads T	4 reads T	Susceptible
#	CYP6P3	AGAP002865	I88T	2R:28492879	Reverse T	Reverse C	32 reads A	25 reads A	27 reads A	25 reads A	Susceptible
#	CYP6P3	AGAP002865	G49D	2R:28492996	Reverse G	Reverse A	40 reads C / 1	27 reads C	33 reads C	28 reads C	Susceptible
#	CYP6P3	AGAP002865	D31E	2R:28493049	Reverse C	Reverse A/G	42 reads G	38 reads G	42 reads G	29 reads G	Susceptible
#	CYP6P3	AGAP002865	A6V	2R:28493125	Reverse C	Reverse T	37 reads G	57 reads G	50 reads G	34 reads G	Susceptible
#	CYP6P5	AGAP002866	E434K	2R:28494247	Reverse G	Reverse A	42 reads C	36 reads C	29 reads C	26 reads C	Susceptible
#	CYP6P5	AGAP002866	P416T	2R:28494301	Reverse C	Reverse A	32 reads G	41 reads G	33 reads G	27 reads G	Susceptible
#	CYP6P5	AGAP002866	E353D	2R:28494587	Reverse G	Reverse T/C	31 reads C	49 reads C	41 reads C	27 reads C	Susceptible
#	CYP6P4	AGAP002867	* 1105+7A?								
#	CYP6P4	AGAP002867	I236M	2R:28497967	Reverse C	Reverse G	31 reads G	39 reads G	38 reads G	42 reads G	Susceptible
#	CYP6P1	AGAP002868	R476H	2R:28499354	Reverse G	Reverse A	26 reads C	37 reads C	33 reads C /	37 reads C	Susceptible
#	CYP6P1	AGAP002868	L374M	2R:28499661	Reverse C	Reverse A	33 reads G	31 reads G	33 reads G	37 reads G	Susceptible
#	CYP6P1	AGAP002868	*1115-5?								
#	CYP6P1	AGAP002868	L355P	2R:28499796	Reverse T	Reverse C	34 reads A / 1	37 reads A	39 reads A	27 reads A	Susceptible
#	CYP6P1	AGAP002868	V313I	2R:28499923	Reverse G	Reverse A	30 reads C	26 reads C	40 reads C	28 reads C	Susceptible
#	CYP6P1	AGAP002868	F81V	2R:28500619	Reverse T	Reverse G	31 reads A	28 reads A	42 reads A	35 reads A	Susceptible
#	CYP6P1	AGAP002868	R73C	2R:28500643	Reverse C	Reverse T	28 reads G	29 reads G	41 reads G	30 reads G	Susceptible
#	CYP6P1	AGAP002868	R30H	2R:28500771	Reverse G	Reverse A	34 reads C	21 reads C	33 reads C	37 reads C	Susceptible
#	CYP6P2	AGAP002869	P477S	2R:28501399	Reverse C	Reverse T	28 reads G	38 reads G	37 reads G	36 reads G / 1 read C	Susceptible
#	CYP6P2	AGAP002869	P449S	2R:28501483	Reverse C	Reverse T	32 reads G	38 reads G	48 reads G	51 reads G	Susceptible
#	CYP6P2	AGAP002869	E53D	2R:28502736	Reverse G	Reverse C/T	31 reads C	33 reads C	35 reads C	30 reads C	Susceptible
#	TEP1	AGAP010815	G919N	3L:11203511	Reverse A	Reverse A	0 reads	0 reads	18 reads C	0 reads	Susceptible
#	TEP1	AGAP010815	A936V	3L:11203459	Reverse T	Reverse T	1 read g	0 reads	25 reads G	1 read G	Susceptible
#	TEP1	AGAP010815	K937N	3L:11203455	Reverse A		1 read T	0 reads	26 reads T	2 read T	Susceptible
#	TEP1	AGAP010815	M946V	3L:11203430	Reverse A		1 read T	0 reads	26 reads T	2 read T	Susceptible
#	TEP1	AGAP010815	S1142C	3L:11202842	Reverse A	Reverse T	0 reads	0 reads	25 reads T	1 read C	Susceptible

Table S8: Comparison of the cytological breakpoints based on polytene chromosome and the inferred breakpoints based on tree topology of the inversions. Cytological breakpoint infered from Coluzzi et al. 2002(*), cytological ranges transformed to Mb in vectorBase *An. gambiae* browser (¨) and infered breakpoints based on tree topology (‘).

	Cytological Breakpoint*		Cytological range (Mb)¨		Inferred Breakpoints (Mb)´	
Inversion	Telomeric	Centromeric	Telomeric	Centromeric	Telomeric	Centromeric
2La	27A.0	23A.0	42.61 - 43.52	20.51 - 22.36	42.2	20.55
3La	45A.0	41A.0	35.09 - 36.54	13.99 - 16.45	35.85	14.5
3Rb	30A.5	30E.5	6.10 - 7.75	11.83 – 13.70	-	-
2Rl	8C.0	12C.7	5.75 – 6.20	22.22 – 23.88	-	-

Table S9: Million years of the 3La inversion. Million years (Ma) estimated from pairwise distances assuming a substitution rate of 1.1x10-9 per site, per generation and 10 generation per year (Tamura et al. 2004). Pairwise distances were estimated between *An. fontenillei* and *An. quadriannulatus* with the windows inside the inversion and +/- 500 kb of the flanking region (3La-inversion), with the windows outside the inversion (3L outside), and, on the X chromosome more common tree. This analysis was repeated with, *An. bwambae* and *An. quadriannulatus* and *An. melas* and *An. quadriannulatus*. Also with *An. fontenillei*, *An. bwambae* and *An. melas* with *An. arabiensis*.

		An. fontenillei - An. quadriannulatus			***An. bwambae - An. quadriannulatus***			***An. melas - An. quadriannulatus***		
**Genomic region**	**Position**	**Media**	**Median**	**Sd**	**Media**	**Median**	**Sd**	**Media**	**Median**	**Sd**
3La-Inversion +/- 500kb fla	14 – 36.4 Mb	2.53	2.33	0.97	2.23	2.09	0.76	2.61	2.54	0.59
3L outside	0 - 14 and 36.4 - 42	1.40	1.30	0.91	1.20	1.21	0.60	1.81	1.81	0.35
X chr more common tree	78 windows	1.25	1.14	0.54	1.25	1.14	0.54	1.82	1.7	0.49

		An. fontenillei - An. arabiensis			***An. bwambae - An. arabiensis***			***An. melas - An. arabiensis***		
**Genomic region**	**Position**	**Media**	**Median**	**Sd**	**Media**	**Median**	**Sd**	**Media**	**Median**	**Sd**
3La-Inversion +/- 500kb fla	14 – 36.4 Mb	2.42	2.24	0.95	2.11	1.97	0.73	2.51	2.43	0.54
3L outside	0 - 14 and 36.4 - 42	1.32	1.25	0.87	1.11	1.09	0.54	1.88	1.9	0.35

Table S10. MAF regions with one or multiple hits. Number of regions per each chromosome arm, for all the genome and proportion of genome used and discard to form the MAF.

	2L	2R	3L	3R	X	ALL	%	Euchromatic genome %
Total MAF regions with 8 species	18246	22763	15493	18679	5593	80774	100	40
Regions with unique hit (in 5 sp)	11664	13310	10158	11552	4378	51062	63.22	25.29
Region with more than 1 hit AgamP3	6331	5660	5062	6811	1097	24961	30.90	12.36
Recovered	1610	1759	1292	1731	367	6759	8.37	3.35
e-value <= 10-4	1361	1420	1037	1433	224	5475	6.78	2.71
Query cover >40%	249	339	255	298	143	1284	1.59	0.64
Region with more than 1 hit Aara	6324	9124	5055	6810	1109	28422	35.19	14.07
Recovered	1620	2080	1277	1679	352	7008	8.68	3.47
e-value <= 10-4	1348	1647	1016	1386	242	5639	6.98	2.79
Query cover >40%	272	406	261	293	128	1360	1.68	0.67
Region with more than 1 hit Aqua	6313	9080	5030	6765	1103	28291	35.02	14.01
Recovered	1504	1896	1192	1614	348	6554	8.11	3.25
e-value <= 10-4	1244	1483	920	1322	221	5190	6.43	2.57
Query cover >40%	260	413	272	292	127	1364	1.69	0.68
Region with more than 1 hit Amel	6233	8982	5001	6715	1065	27996	34.66	13.86
Recovered	1560	1445	1233	1672	326	6236	7.72	3.09
e-value <= 10-4	1248	1624	969	1361	197	5399	6.68	2.67
Query cover >40%	312	306	264	311	129	1322	1.64	0.65
Region with more than 1 hit Amer	6306	9083	5033	6756	1094	28272	35.00	14.00
Recovered	1602	2111	1285	1711	363	7072	8.76	3.50
e-value <= 10-4	1287	1637	995	1384	211	5514	6.83	2.73
Query cover >40%	315	474	290	327	152	1558	1.93	0.77
All recovered (not repetead among species)	2251	2859	1795	2330	472	9707	12.02	4.81
ALL 1 HIT (analyzed)	13915	16168	11953	13882	4850	60768	75.23	30.09
Others (not analysed)	4331	6595	3540	4797	743	20006	24.77	9.91

Figure S1

Specimen 8

Specimen 23

Specimen 35

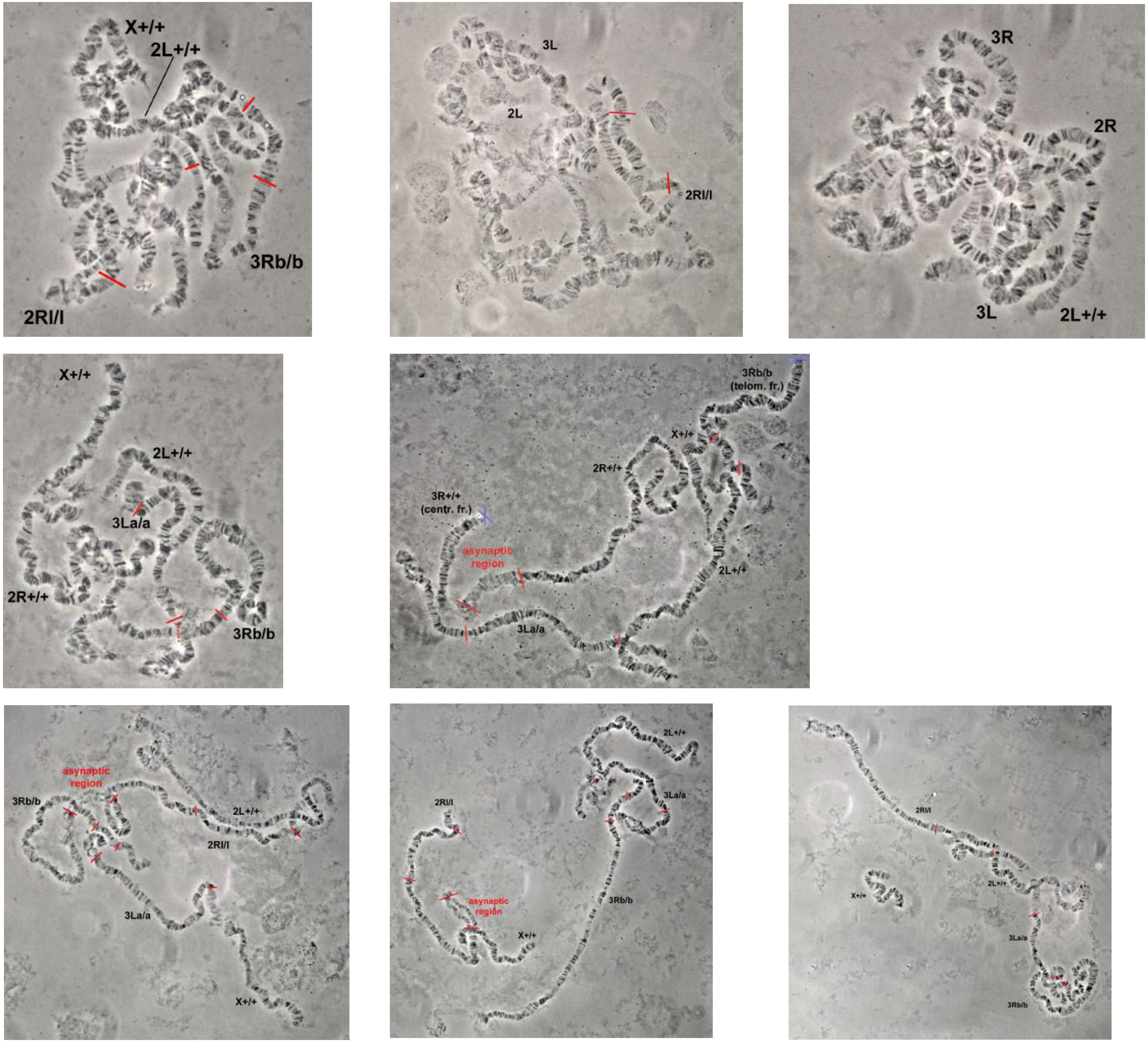

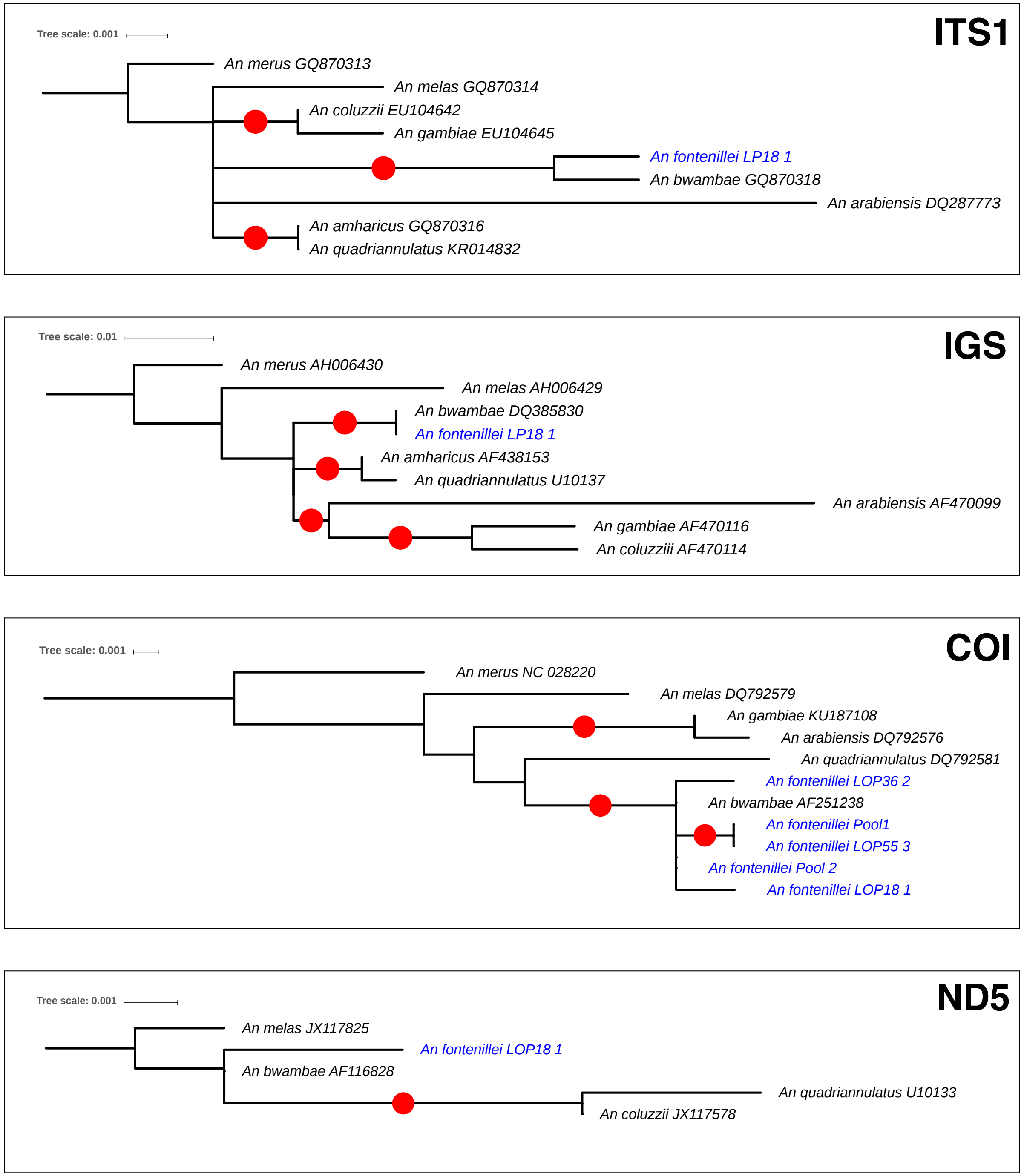

ITS1
Tree scale: 0.001
An merus GQ870313
An melas GQ870314
An coluzzii EU104642
An gambiae EU104645
An fontenillei LP18 1
An bwambae GQ870318
An arabiensis DQ287773
An amharicus GQ870316
An quadriannulatus KR014832
IGS
Tree scale: 0.01
An merus AH006430
An melas AH006429
An bwambae DQ385830
An fontenillei LP18 1
An amharicus AF438153
An quadriannulatus U10137
An arabiensis AF470099
An gambiae AF470116
An coluzziii AF470114
COI
Tree scale: 0.001
An merus NC 028220
An melas DQ792579
An gambiae KU187108
An arabiensis DQ792576
An quadriannulatus DQ792581
An fontenillei LOP36 2
An bwambae AF251238
An fontenillei Pool1
An fontenillei LOP55 3
An fontenillei Pool 2
An fontenillei LOP18 1
ND5
Tree scale: 0.001
An melas JX117825
An fontenillei LOP18 1
An bwambae AF116828
An quadriannulatus U10133
An coluzzii JX117578

Figure S3

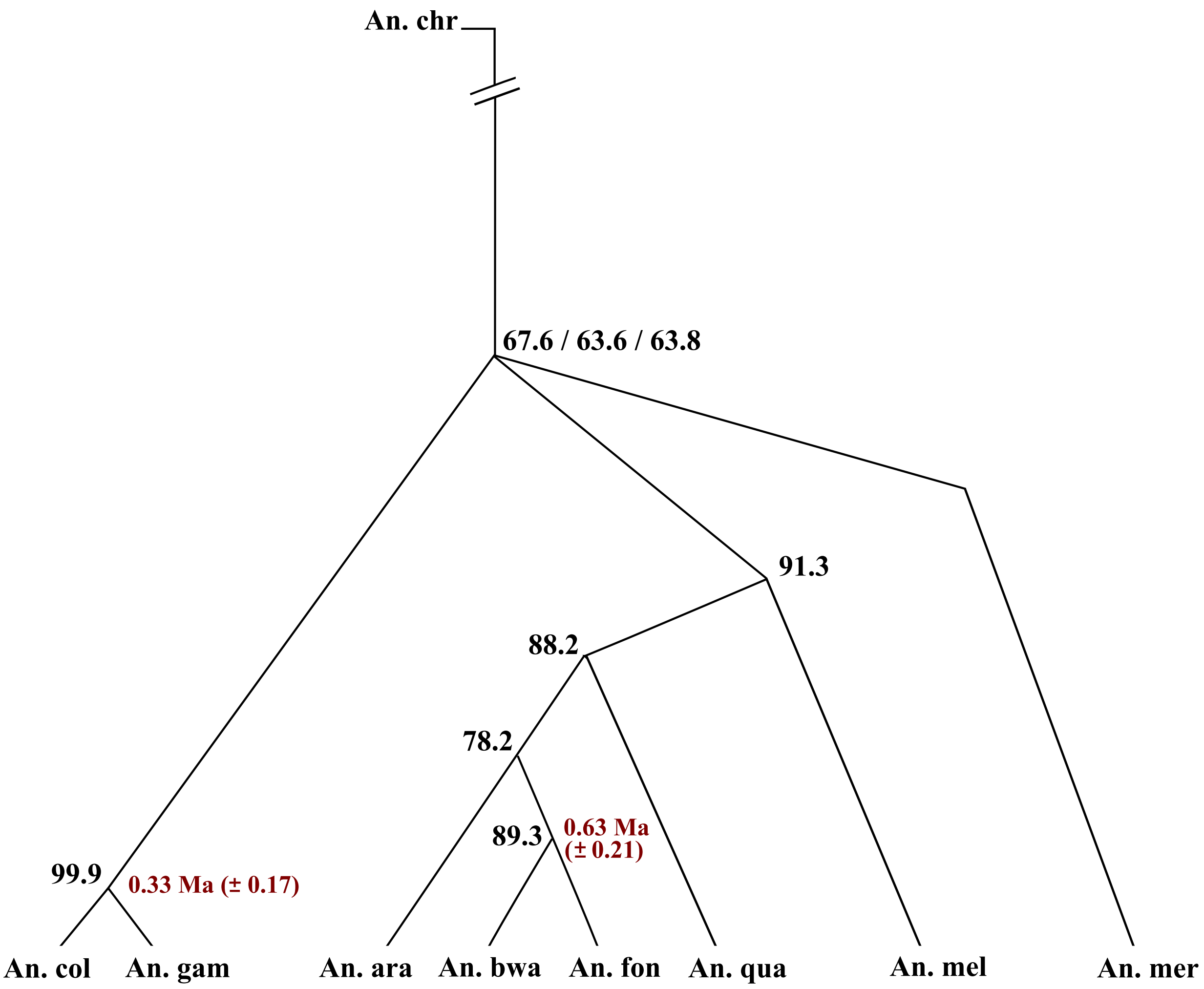

An. chr
67.6 / 63.6 / 63.8
91.3
88.2
78.2
89.3
0.63 Ma
(± 0.21)
99.9
0.33 Ma (± 0.17)
An. col
An. gam
An. ara
An. bwa
An. fon
An. qua
An. mel
An. mer

Figure S4

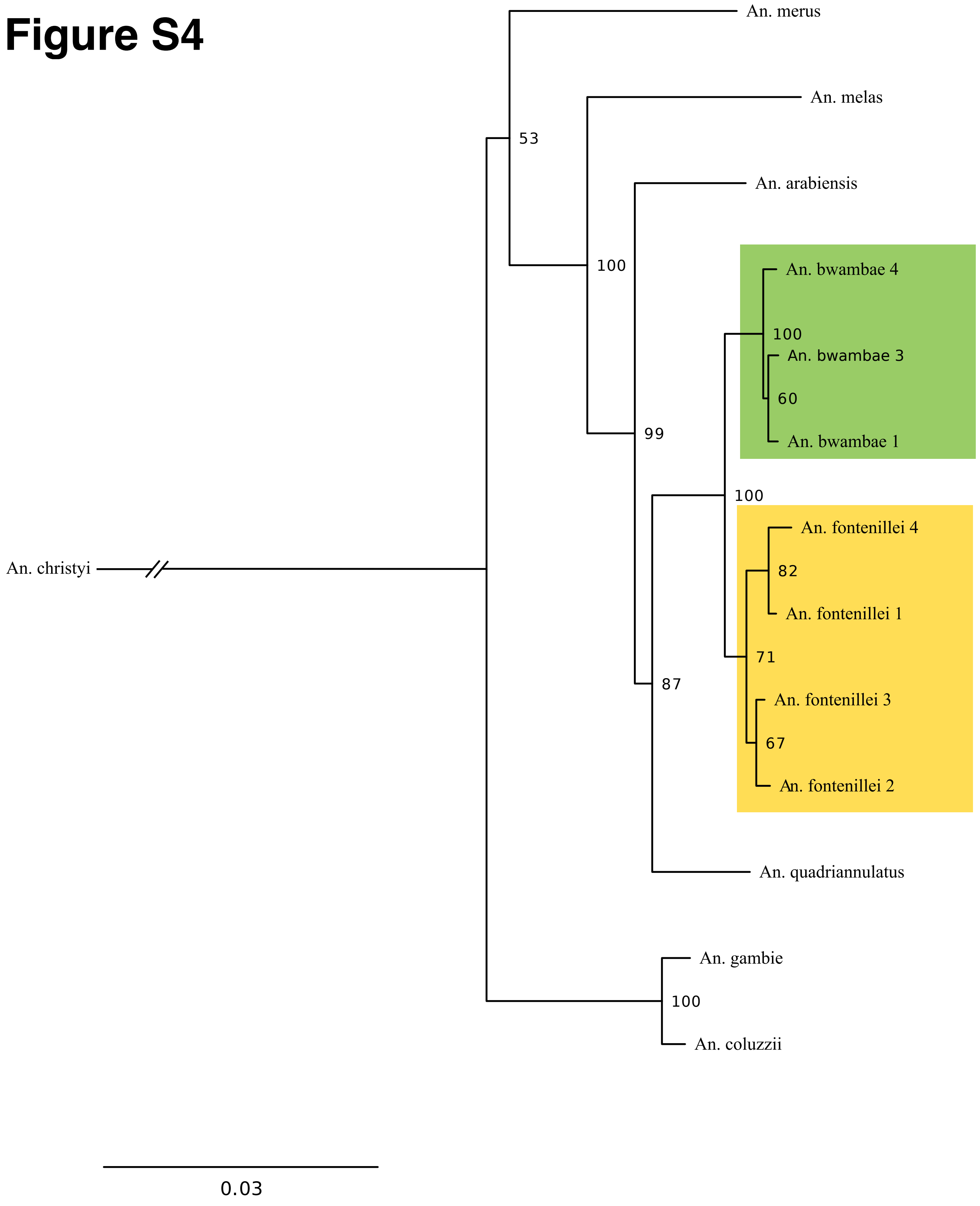

An. merus
An. melas
53
An. arabiensis
100
An. bwambae 4
100
An. bwambae 3
60
An. bwambae 1
99
100
An. fontenillei 4
An. christyi
82
An. fontenillei 1
71
87
An. fontenillei 3
67
An. fontenillei 2
An. quadriannulatus
An. gambie
100
An. coluzzii
0.03

A

B

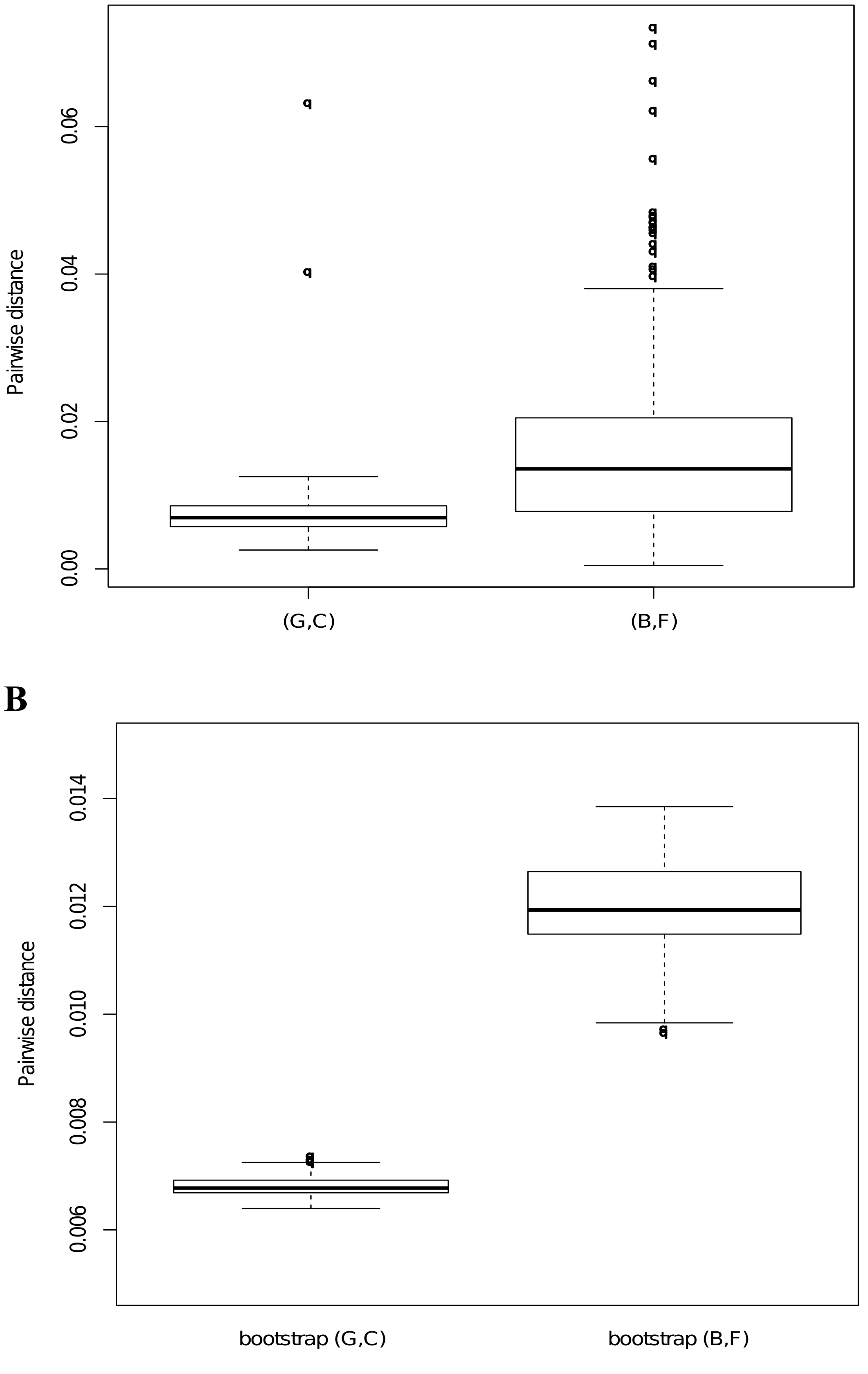
Pairwise distance
0.00
0.02
0.04
0.06
(G,C)
(B,F)
Pairwise distance
0.006
0.008
0.010
0.012
0.014
bootstrap (G,C)
bootstrap (B,F)

Figure S5

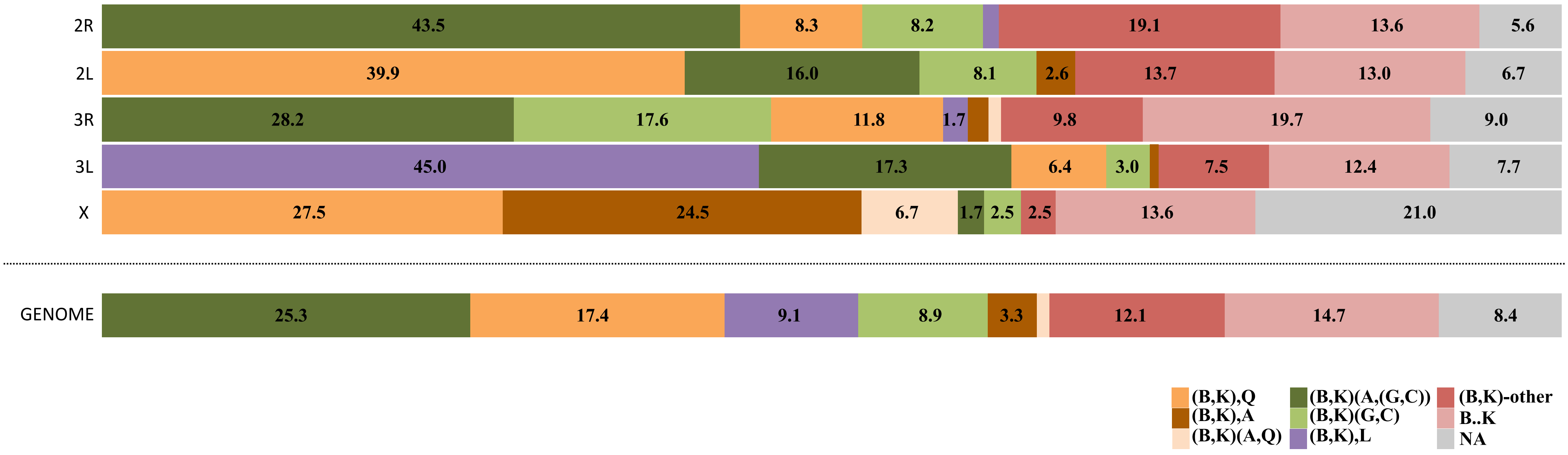
2R
43.5
8.3
8.2
19.1
13.6
5.6
2L
39.9
16.0
8.1
2.6
13.7
13.0
6.7
3R
28.2
17.6
11.8
1.7
9.8
19.7
9.0
3L
45.0
17.3
6.4
3.0
7.5
12.4
7.7
X
27.5
24.5
6.7
1.7
2.5
2.5
13.6
21.0
GENOME
25.3
17.4
9.1
8.9
3.3
12.1
14.7
8.4
(B,K),Q
(B,K),A
(B,K)(A,Q)
(B,K)(A,(G,C))
(B,K)(G,C)
(B,K),L
(B,K)-other
B..K
NA

Figure S6

Figure S7

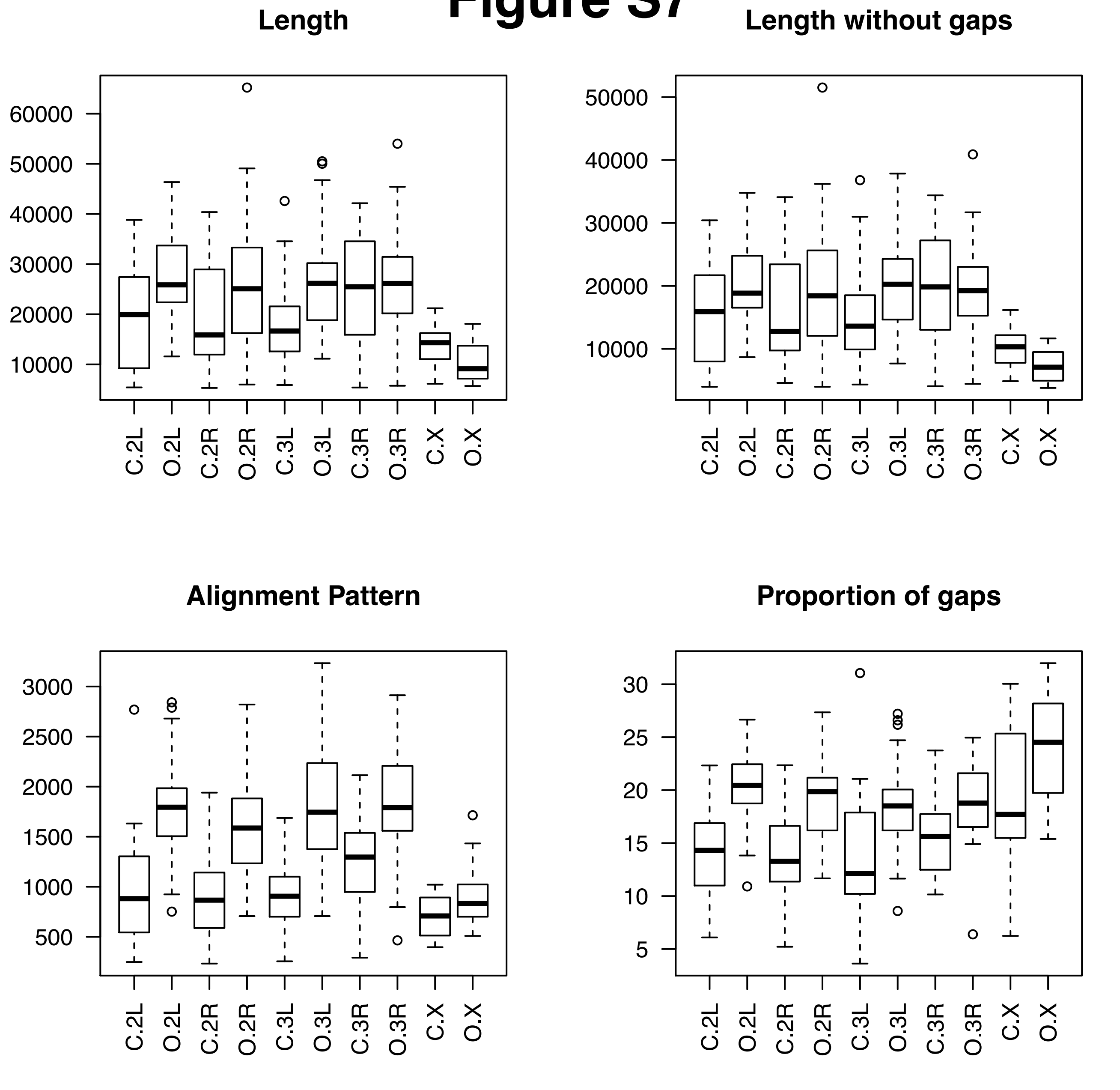

Length
Length without gaps
Alignment Pattern
Proportion of gaps
C.2L
O.2L
C.2R
O.2R
C.3L
O.3L
C.3R
O.3R
C.X
O.X

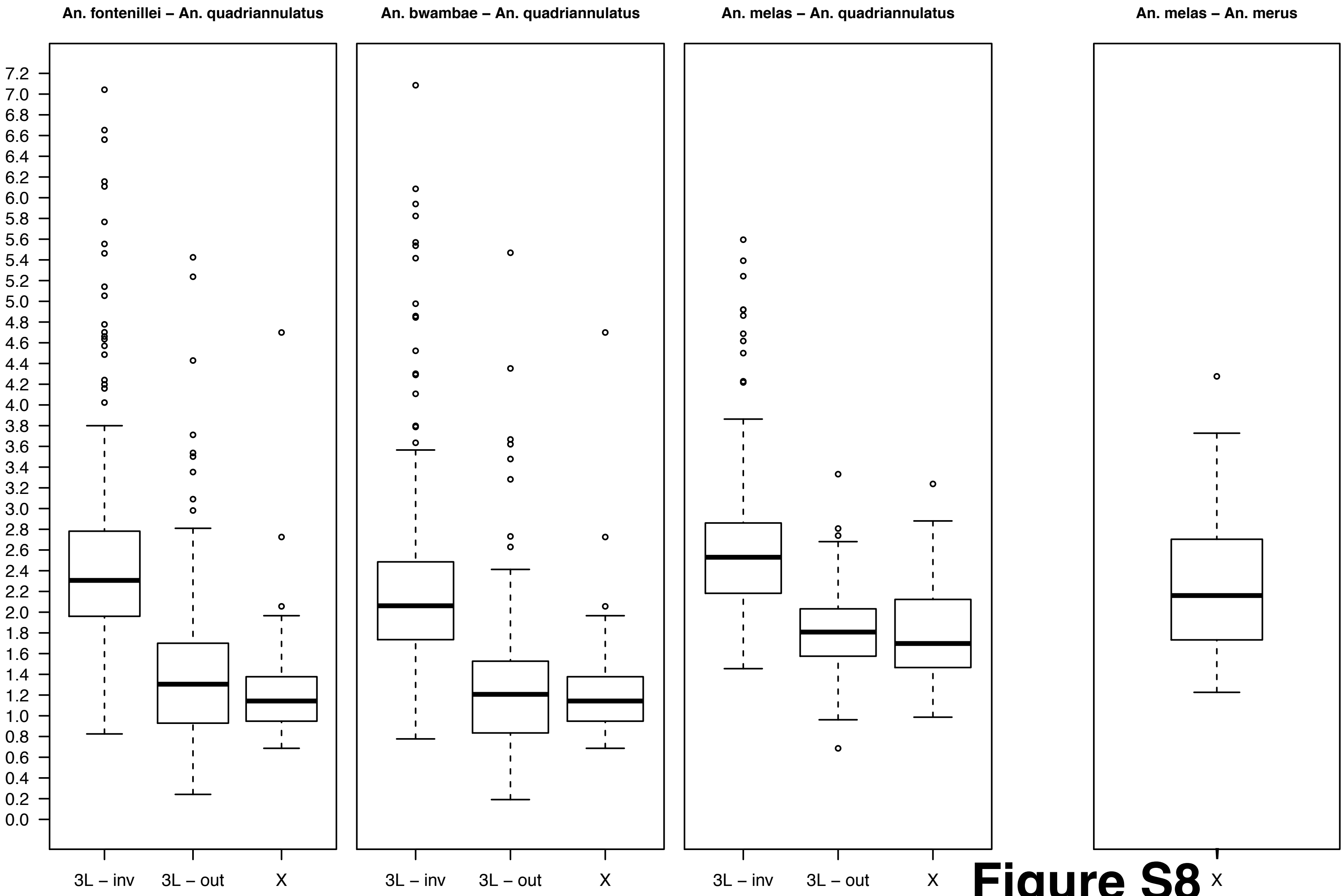
An. fontenillei – An. quadriannulatus
An. bwambae – An. quadriannulatus
An. melas – An. quadriannulatus
An. melas – An. merus
7.2
7.0
6.8
6.6
6.4
6.2
6.0
5.8
5.6
5.4
5.2
5.0
4.8
4.6
4.4
4.2
4.0
3.8
3.6
3.4
3.2
3.0
2.8
2.6
2.4
2.2
2.0
1.8
1.6
1.4
1.2
1.0
0.8
0.6
0.4
0.2
0.0
3L – inv
3L – out
X
3L – inv
3L – out
X
3L – inv
3L – out
X
X

Figure S8

Text S1. *Taxonomic description of the new taxon*

Morphological description of *Anopheles (Cellia) fontenillei* n. sp. Rahola & Paupy & Ayala

Male, pupae: unknown.

Larvae: unknown, collected but not described.

Type locality: La Lopé National Park, Gabon.

Type data and depository: Holotype: *An. fontenillei* n.sp. N°3, female: La Lopé National Park, Bosquet buffle proche station SEGC, Gabon (S0.19773°; E11.60041°, 264m) 12/06/2015, Human landing capture, two slides with the mounting of the wing and the hind leg are associated and recorded as XXX. Deposited in the Institut de Recherche pour le Développement, Montpellier, France.

Other material data and depository: Paratypes: deposited in the same institution with the labels as follow: *An. fontenillei* n.sp. N°473, female, LOP40 (S0.20336°; E11.60197°), 02/02/2016, larval rearing, La Lopé National Park, Gabon; *An. fontenillei* n.sp. N°781, female, LOP51 (S0.20356°; E11.60281°), 09/02/2016, larval rearing, La Lopé National Park, Gabon; *An. fontenillei* n.sp. N°1, female, LOPXXX, 02/02/2016, larval rearing, La Lopé National Park, Gabon and a slide of the wing recorded as XXX; *An. fontenillei* n.sp. N°2, female, LOPXXX, 02/02/2016, larval rearing, La Lopé National Park, Gabon and a slide of the wing recorded as XXX

An. fontenillei belongs to the subgenus *Cellia* on the basis of more than four pale spots on the costa and (its well-developed cibarial armature.)

Head: Interocular space moderately broad with 16 setae and also some long narrow flat falcate white scales. Vertex with numerous erect black and white forked scales, white ones restricted to anterior part forming a sort of white triangle. Some long narrow flat falcate white scales present on extreme anterior part of vertex just before

interocular space. Pedicel with some setae and narrow erected scales, 1st antennal flagellomere with white scales reaching the next segment, 2nd antennal flagellomere shorter than first. Maxillary palpus with 3 white rings, 3rd palpomere white basally and apically and dark in the middle, 5th palpomere entirely white (Fig.X).

Thorax: Scutum with greyish cuticle and numerous erect white falcate scales on median and lateral area. Scutellum greyish with dark brown spot on cuticle in middle and some white falcate scales. Wing, see (Fig.X). Halters with white narrow scales. Legs with pale speckles as species from the Gambiae complex (Fig.X).

Abdomen: Cuticule entirely black with numerous fine setae, few white narrow falcate scales present on 8th tergite.

Wing variations: Wings of four specimens exhibited variations as follow: vein 1A with 3 dark spots on specimen N°473 and N°1; base of subcosta without some black scales in all paratype material. Presence of the white spot on wing fringe between end of 1A and end of M3+4 can move or be absent.

Etymology: we dedicate this species to our dear colleague Didier Fontenille who is contributing greatly to the study of mosquitoes and medical entomology.

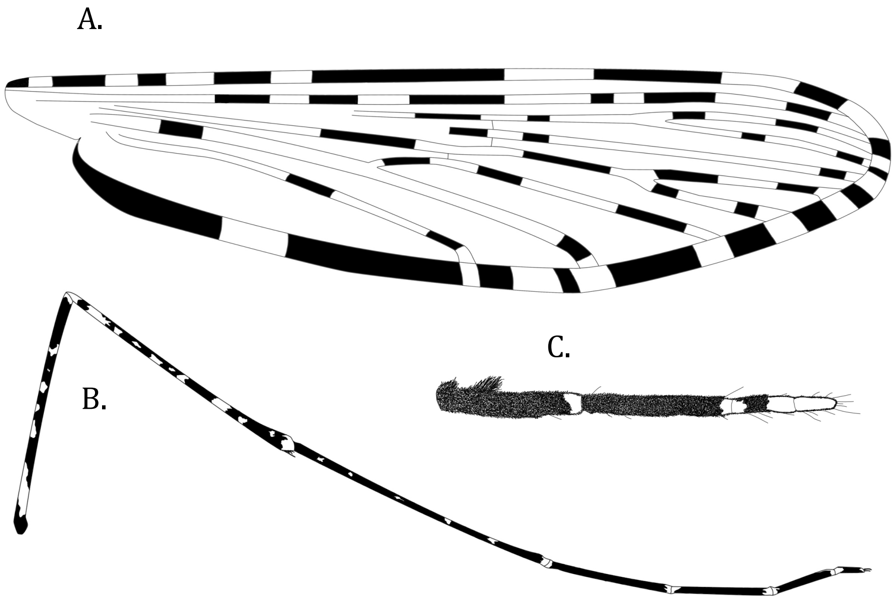

Wing (A.), hind leg (B) and palpus (C) of *An. fontenillei* n.sp.

```
################################################################
############       Anopheles fontenillei proyect    ############
############         Maite Barrón 2017              ############
################################################################

################################################################
##########################  INDEX  #############################
################################################################

1.- Blast assembly to MAF
#1.1- Make a database with the An. fontenillie assembly scaffolds, line 47
#1.2.- Trasnform MAF to fasta, line 51
#1.3.- Make a blastn for each region on the MAF with AgamP3 as query, line 72
#1.4.- Repeat the blastn for each region on the MAF with other species as query, line 79
#1.5.- Select multiple hit fragement and apply the filtering, line 101
#1.6.- Prepare sequences to MAF. Cut the scaffolds according to blast output, line 119
2.- MAFFT to generate an upated new MAF
#2.1.- Run MAFFT to add the sequence to the alignment, line 167
#2.2.-fasta to nexus, line 184
#2.3.- add fasta to MAF format to generate the new MAF, line 213
3.- An. bwamabe sequence donwloading
#3.1.-Donwload sequences, 988
4.- An. bwamabe mapping to AgamP3
#4.1.-Illumina Read Quality Control, line 1003
#4.2.-Trimmed the reads, line 1008
#4.3.-Trimmed Illumina Read Quality Control, line 1026
#4.4.-Mapping with BWA-mem, line 1030
#4.5.-Cleanning the alignment, line 1038
#4.6.-Sorting by reference, line 1044
#4.7.- Mark Duplicates, line 1051
#4.8.- Re-aling around indels, line 1060
5.- An. bwambae from fastq to MAF
#5.1.- mpileup of each MAF region (in fastq format), line 1108
#5.2.- mpileup of each MAF region (in fasta format), line 1120
6.- Window-based phylogeny analysis
#6.1.- Fragmentate MAF in windows, line 1158
#6.2.- Run RAxML by window, line 1424
#6.3.- Phylogenetic tree topology sorting and counting, line 1435
7.- R. Genetic pairwise distances and bootstrapping
#7.1 R code for estimating Genetin pairwise distances, line 2216
#7.2 R code for performing the boostrapping, line 2221

################################################################
############## 1.- Blast assembly to MAF ######################
################################################################

#1.1- Make a database with the An. fontenillie assembly scaffolds
module load BLAST+/2.4.0
makeblastdb -in assembly.fa -parse_seqids -dbtype nucl

#1.2.- Trasnform MAF to fasta
#!/usr/bin/perl
use warnings;
use strict;

opendir(DH, "MAF_regions/"); #Folder were the MAF regions with 8 speceies are, each in a
file.
my @files = readdir(DH);
closedir(DH);

foreach my $file (@files){ # For each MAF region with the 8 species information
    next if($file =~ /^\.$/);
    next if($file =~ /^\.\.$/);
    open (MAF, "MAF_regions/$file") or die "cannot open file";
```

```
    open (OUT, ">MAF_regions/$file.fasta") or die "cannot create file";
    while (<MAF>){
        if ($_ =~ /^s\s(AgamP3.chr$chr)\s+(\d+)\s\d+.*\s([ATGC-]+)/){
                print OUT ">$1_$2\n$3\n";
        }
    }
}

#1.3.- Make a blastn for each region on the MAF with AgamP3 as query
module load BLAST+/2.4.0
while read i <&3
do
blastn -query each_MAF_region_of_AgamP3.fasta -db assembly.fa -out
blast_output_maf_to_assembly -outfmt "7"
done 3<MAF_fragment_positions.txt

#1.4.- Repeat the blastn for each region on the MAF with other species as query
module load BLAST+/2.4.0
while read i <&3
do
blastn -query each_MAF_region_of_Aara.fasta -db assembly.fa -out
blast_output_maf_to_assembly -outfmt "7"
done 3<MAF_fragment_positions.txt

while read i <&3
do
blastn -query each_MAF_region_of_Aqua.fasta -db assembly.fa -out
blast_output_maf_to_assembly -outfmt "7"
done 3<MAF_fragment_positions.txt

while read i <&3
do
blastn -query each_MAF_region_of_Amel.fasta -db assembly.fa -out
blast_output_maf_to_assembly -outfmt "7"
done 3<MAF_fragment_positions.txt

while read i <&3
do
blastn -query each_MAF_region_of_Amer.fasta -db assembly.fa -out
blast_output_maf_to_assembly -outfmt "7"
done 3<MAF_fragment_positions.txt

#1.5.- Select multiple hit fragement and apply the filtering
#!/usr/bin/perl
use warnings;
use strict;

open (BLAST, "multiple_hit_list.txt") or die "cannot open blast file";
open (OUT, ">multiple_hit_list_filtered.txt") or die "cannot create blast file";

while (<BLAST>){
     $_ =~ s/\n//g;
     my $bl = $_;
     my @inf = split ("\t", $bl);
     my $prop_query_cover = sprintf("%.2f",((100*$inf[7])/$inf[3]));
     if ( $inf[14] ne "0.0" && $inf[14] ne "0.001" && $inf[14] ne "0.01" && $inf[14] ne
"0.00" && $prop_query_cover > 40) {   #<1e-04
          print OUT "$bl\t$prop_query_cover\n";
     }
}

#1.6.- Prepare sequences to MAF. Cut the scaffolds according to blast output
#!/usr/bin/perl
use warnings;
```

```
my $chr = $ARGV[0];
my $i = 0;
my $p = 0;
my $seq = '';
my $actual = '';

open (INFO, "blast_hit.txt") or die "cannot open blast file";

while (<INFO>){
        $_ =~ s/\n//g;
        my @inf = split ("\t", $_);
        open (OUT, ">scaffold_regions_blast_with_MAF/$chr/$inf[5]"."_$inf[1].fasta")or
die "cannot create file";;
        open (ASS, "assembly.fa") or die "cannot open file";
        while (<ASS>){
                $_ =~ s/\n//g;
                $actual = $_;
                if ($actual =~ /^>$inf[5]$/){
                        print OUT $actual."\n";
                         $i++;
                }elsif ($i > 0){
                         $seq = $actual;
                         $i = 0;
                         $p++;
                }
                if ($inf[12] < $inf[13] && $p > 0){
                        my $chunk = substr $seq, $inf[12]-1, $inf[7];
                        print OUT $chunk."\n";
                        $p = 0;
                }elsif ($inf[12] > $inf[13] && $p > 0){
                        my $chunk = substr $seq, $inf[13]-1, $inf[7];
                        my $rev = reverse $chunk;
                        $rev =~ tr/ATGC/TACG/;
                        print OUT $rev."\n";
                        $p = 0;
                }
        }
}


###############################################################
############# 2.- MAFFT to generate an updated new MAF ########
###############################################################

#2.1.- Run MAFFT to add the sequence to the alignment .

#!/bin/bash -l
while read i <&3 && read p <&4;
do
qsub -l h_vmem=2G -cwd -b y run_mafft.sh $i $p
done 3<MAF_position.txt 4<scaffold_name.txt

#run_mafft.sh
#!/bin/bash -l
module load MAFFT/7.245

pos=$1
name=$2

mafft --add scaffold_fragments/$name\_$pos.fasta --reorder MAF_regions/tba_HD_AgamC9_
$pos.maf.fasta > output_MAFFT/tba_HD_AgamC9_maf_$coor.fasta
```

```
#2.2.- fasta to nexus
#!/usr/bin/perl
use warnings;
use strict;

opendir(DH, "output_MAFFT/");
my @files = readdir(DH);
closedir(DH);

foreach my $file (@files){
        next if($file =~ /^\.$/);
        next if($file =~ /^\.\.$/);

        $file =~ /^(tba.*).fasta/;
        print "$1\n";
        open (FA, "<output_MAFFT/$file") or die "cannot open file";
        open (OUT, ">output_MAFFT/$1.nex") or die "cannot open file"
            while (<FA>){
                $_ =~ s/\n//g;
                if ($_ =~ /^>/){
                    print OUT "\n$_\t";
                }elsif ($_ =~ /^[^>]/){
                    print OUT "$_";
                }
            }
        close FA;
        close OUT;
}

#2.3.- add fasta to MAF format to generate the new MAF.
#!/usr/bin/perl
use warnings;
use strict;
#use Data::Dumper;


#################
##  CONSTANTS  ##
#################
# Debug flags
my $debug = 0;
my $verbose = 0;

# Input file ".MAF"
my $MAFfilename = "tba.aligned.maf";
my $MAFcolSeparator = "\t";                 # Column separator
my $MAFcolType = 0;                             # Column index for the type of row (s:
sequence; a: analysis)
my $MAFcolSpecies = 1;                      # Column index for the species name
my $MAFcolPos = 2;                          # Column index for the genomic position
my $MAFcolSeq = 6;                          # Column index for the sequence (ACGT-)
my @MAFspecies = qw(AgamP3 AgamS1 AgamM1 AmerM1 AaraD1 AquaS1 AmelC1 AchrA1 AepiE1);
                   # Species present in "$MAFfilename"
my $RefSpecies = $MAFspecies[0];
                                         # Reference species in "$MAFfilename"
my @NewSpecies = qw(scaffold_%d);
my @AllSpecies = @MAFspecies[0..$#MAFspecies-2];
push(@AllSpecies, @NewSpecies, @MAFspecies[$#MAFspecies-1..$#MAFspecies]);
                                    # Species sorted as desired in the output file

# Input file ".NEX"
my $NEXfilename = "tba_HD_AgamC9_maf_%d.nex";
my $NEXcolSeparator = "\t";                 # Column separator
my $NEXcolSpecies = 0;                      # Column index for the species name
```

```
my $[illegible]ColSeq = [illegible];  # Column index for the sequence (ACGT-)
my $TrimCharacter = "-";

# Output file ".MAF"
my $OutFilename = "/homes/users/mbarron/scratch/Anopheles/assembly_to_MAF/output_MAF/
tba_HD_AgamC9_fontenillei.maf";

##################
##  SUBROUTINES  ##
##################
#-----------------------------------------------------#
# import_csv_file($filename, $ColumnSeparator:"\t") #
#-----------------------------------------------------#
sub import_csv_file
{
if ($debug) {printf "import_csv_file\n";}
	# Read input parameters
	my $filename;
	my $ColumnSeparator = "\t";
	if ($#_==0) {$filename = $_[0];}
	elsif ($#_==1) {$filename = $_[0]; $ColumnSeparator = $_[1];}
	else {die "\n!!!!!FATAL ERROR!!!!!\nWrong input arguments in function
\"import_csv_file\"$!";}

	# Read input file
	my $InputFile;
	open ($InputFile, "<", $filename) or die "\n!!!!!FATAL ERROR!!!!!\nCan't open file
\"$filename\"$!";
	my @ResultAux = <$InputFile>;
	close $InputFile;

	# Build 2D list
	my @result;
	for (my $i=0; $i<=$#ResultAux; $i++)
	{
		$ResultAux[$i] =~ s/\n//g;
				# Delete end of line characters
		my @aux = split("$ColumnSeparator", $ResultAux[$i]);
		# Separate columns by "tabs"
		for (my $j=0; $j<=$#aux; $j++)
		{
			$result[$i][$j] = $aux[$j];
		}
	}
	return @result;
}

#----------------------------#
# find_index(\@list, $text) #
#----------------------------#
sub find_index
{
if ($debug && $verbose) {printf "find_index\n";}
	# Read input parameters
	my @list;
	my $text;
	if ($#_==1) {@list = @{$_[0]}; $text = $_[1];}
	else {die "\n!!!!!FATAL ERROR!!!!!\nWrong input arguments in function \"find_index
\"$!";}

	use List::Util qw(first);
	my $result = first {$list[$_] eq $text} 0..$#list;
	if (defined($result))
	{
```

```
		return $result;
	}
	else
	{
		return -1
	}
}

#---------------------------------------#
# delete_empty_lines_in_2D_list(\@list) #
#---------------------------------------#
sub delete_empty_lines_in_2D_list
{
if ($debug) {printf "delete_empty_lines_in_2D_list\n";}
	# Read input parameters
	my @list;
	if ($#_==0) {@list = @{$_[0]};}
	else {die "\n!!!!!FATAL ERROR!!!!!\nWrong input arguments in function 
\"delete_empty_lines_in_2D_list\"$!";}

	# Delete empty lines
	my @result;
	my $line=0;
	for(my $i=0; $i<=$#list; $i++)
	{
		if($list[$i])
		{
			$result[$line] = $list[$i];
			$line++;
		}
	}
	return @result;
}

#--------------------------------------------------#
# delete_column_in_2D_list(\@list, $ColumnIndex) #
#--------------------------------------------------#
sub delete_column_in_2D_list
{
if ($debug) {printf "delete_column_in_2D_list\n";}
	# Read input parameters
	my @list;
	my $ColumnIndex;
	if ($#_==1) {@list = @{$_[0]}; $ColumnIndex = $_[1];}
	else {die "\n!!!!!FATAL ERROR!!!!!\nWrong input arguments in function 
\"delete_column_in_2D_list\"$!";}

	# Delete column
	my @result;
	for(my $i=0; $i<=$#list; $i++)
	{
		my $col=0;
		for(my $j=0; $j<=$#{$list[$i]}; $j++)
		{
			if($j != $ColumnIndex)
			{
				$result[$i][$col] = $list[$i][$j];
				$col++;
			}
		}
	}
	return @result;
}
```

```
# sort_2D_list_by_column(\@list, $ColumnIndex) #
#--------------------------------------------#
sub sort_2D_list_by_column
{
if ($debug) {printf "sort_2D_list_by_column\n";}
     # Read input parameters
     my @list;
     my $ColumnIndex;
     if ($#_==1) {@list = @{$_[0]}; $ColumnIndex = $_[1];}
     else {die "\n!!!!!FATAL ERROR!!!!!\nWrong input arguments in function
\"sort_2D_list_by_column\"$!";}

     # Sort list by column
     return sort {$a->[$ColumnIndex] cmp $b->[$ColumnIndex]} @list;
}

#----------------------------------------------------------------------------#
# sort_2D_list_as_reference_column(\@list, \@ReferenceColumn, $ColumnIndex) #
#----------------------------------------------------------------------------#
sub sort_2D_list_as_reference_column
{
if ($debug && $verbose) {printf "sort_2D_list_as_reference_column\n";}
     # Read input parameters
     my @list;
     my @ReferenceColumn;
     my $ColumnIndex;
     if ($#_==2) {@list = @{$_[0]}; @ReferenceColumn = @{$_[1]}; $ColumnIndex = $_[2];}
     else {die "\n!!!!!FATAL ERROR!!!!!\nWrong input arguments in function
\"sort_2D_list_as_reference_column\"$!";}

     # Sort 2D list as indicated by @ReferenceColumn
     return sort {find_index(\@ReferenceColumn, (split(/\./, $a->[$ColumnIndex]))[0]) <=>
find_index(\@ReferenceColumn, (split(/\./, $b->[$ColumnIndex]))[0])} @list;
}

#-----------------------------------------------------#
# select_lines_in_2D_list(\@list, $ColumnIndex, $text) #
#-----------------------------------------------------#
sub select_lines_in_2D_list
{
if ($debug) {printf "select_lines_in_2D_list\n";}
     # Read input parameters
     my @list;
     my $ColumnIndex;
     my $text;
     if ($#_==2) {@list = @{$_[0]}; $ColumnIndex = $_[1]; $text = $_[2];}
     else {die "\n!!!!!FATAL ERROR!!!!!\nWrong input arguments in function
\"select_lines_in_2D_list\"$!";}

     # Select lines
     my @result;
     my $j=0;
     for (my $i=0; $i<=$#list; $i++)
     {
          if (defined($list[$i][$ColumnIndex]) && ($list[$i][$ColumnIndex] eq $text))
          {
               $result[$j] = $list[$i];
               $j++;
          }
     }
     return @result;
}
```

```
# select_column_in_2D_list(\@list, $ColumnIndex) #
#-----------------------------------------------#
sub select_column_in_2D_list
{
if ($debug) {printf "select_column_in_2D_list\n";}
     # Read input parameters
     my @list;
     my $ColumnIndex;
     if ($#_==1) {@list = @{$_[0]}; $ColumnIndex = $_[1]}
     else {die "\n!!!!!FATAL ERROR!!!!!\nWrong input arguments in function
\"select_column_in_2D_list\"$!";}

     # Select column
     my @result;
     for (my $i=0; $i<=$#list; $i++)
     {
          if (defined($list[$i][$ColumnIndex]))
          {
               $result[$i] = $list[$i][$ColumnIndex];
          }
     }
     return @result;
}

#------------------------------------------------------------#
# delete_character_in_2D_list(\@list, $ColumnIndex, $regex) #
#------------------------------------------------------------#
sub delete_character_in_2D_list
{
if ($debug) {printf "delete_character_in_2D_list\n";}
     # Read input parameters
     my @list;
     my $ColumnIndex;
     my $regex;
     if ($#_==2) {@list = @{$_[0]}; $ColumnIndex = $_[1]; $regex = $_[2];}
     else {die "\n!!!!!FATAL ERROR!!!!!\nWrong input arguments in function
\"delete_character_in_2D_list\"$!";}

     for (my $i=0; $i<=$#list; $i++)

     {
          $list[$i][$ColumnIndex] =~ s/$regex//;
     }
     return @list;
}

#-------------------------------------------#
# print_1D_list(\@list, $ColumnSeparator) #
#-------------------------------------------#
sub print_1D_list
{
if ($debug) {printf "print_1D_list\n";}
     # Read input parameters
     my @list;
     my $ColumnSeparator = "\t";
     if ($#_==0) {@list = @{$_[0]};}
     elsif ($#_==1) {@list = @{$_[0]}; $ColumnSeparator = $_[1];}
     else {die "\n!!!!!FATAL ERROR!!!!!\nWrong input arguments in function
\"print_1D_list\"$!";}

     # Print 1D list
     for (my $i=0; $i<=$#list; $i++)
     {
```

```
		printf "$list[$i]";
		if ($i<$#list)
		{
			printf "$ColumnSeparator";
		}
	}
	printf "\n";
	return
}

#-------------------------------------------#
# print_2D_list(\@list, $ColumnSeparator) #
#-------------------------------------------#
sub print_2D_list
{
if ($debug) {printf "print_2D_list\n";}
	# Read input parameters
	my @list;
	my $ColumnSeparator = "\t";
	if ($#_==0) {@list = @{$_[0]};}
	elsif ($#_==1) {@list = @{$_[0]}; $ColumnSeparator = $_[1];}
	else {die "\n!!!!!FATAL ERROR!!!!!\nWrong input arguments in function 
\"print_2D_list\"$!";}

	# Print 2D list
	for (my $i=0; $i<=$#list; $i++)
	{
		if ($list[$i]) {print_1D_list($list[$i], $ColumnSeparator);}
		else {printf "\n";}
	}
	return
}

#-------------------------------------------------------------#
# print_2D_list_to_file(\@list, $filename, $ColumnSeparator) #
#-------------------------------------------------------------#
sub print_2D_list_to_file
{
if ($debug) {printf "print_2D_list_to_file\n";}
	# Read input parameters
	my @list;
	my $filename;
	my $ColumnSeparator = "\t";
	if ($#_==1) {@list = @{$_[0]}; $filename = $_[1];}
	elsif ($#_==2) {@list = @{$_[0]}; $filename = $_[1]; $ColumnSeparator = $_[2];}
	else {die "\n!!!!!FATAL ERROR!!!!!\nWrong input arguments in function 
\"print_2D_list_to_file\"$!";}

	# Print 2D list
	open (OUT, ">", $filename) or die "\n!!!!!FATAL ERROR!!!!!\nCan't open file 
\"$filename\"$!";
	for (my $i=0; $i<=$#list; $i++)
	{
		if ($list[$i])
		{
			for (my $j=0; $j<=$#{$list[$i]}; $j++)
			{
				printf OUT "$list[$i][$j]";
				if ($j<$#{$list[$i]})
				{
					printf OUT "$ColumnSeparator";
				}
			}
		}
```

```
        printf OUT "\n";
    }
    close OUT;
    return
}

#-------------------------#
# regex_to_string($regex) #
#-------------------------#
sub regex_to_string
{
if ($debug) {printf "regex_to_string\n";}
    # Read input parameters
    my $regex;
    if ($#_==0) {$regex = $_[0];}
    else {die "\n!!!!!FATAL ERROR!!!!!\nWrong input arguments in function
\"regex_to_string\"$!";}

    # Convert regex to string
    my $result = sprintf($regex);
    $result =~ s/($regex)//;
    return $1;
}

#---------------------------------------------------------#
# check_marker_balance($string, $StartRegex, $EndRegex) #
#---------------------------------------------------------#
sub check_marker_balance
{
if ($debug) {printf "check_marker_balance\n";}
    # Read input parameters
    my $string;
    my $StartRegex;
    my $EndRegex;
    if ($#_==2) {$string = $_[0]; $StartRegex = $_[1]; $EndRegex = $_[2];}
    else {die "\n!!!!!FATAL ERROR!!!!!\nWrong input arguments in function
\"check_marker_balance\"$!";}

    # Check marker balance
    my $depth = 0;
    for (my $i=0; $i<length($string); $i++)
    {
        if (substr($string, $i, 1) =~ m/$StartRegex/)
        {
            $depth++;
        }
        elsif (substr($string, $i, 1) =~ m/$EndRegex/)
        {
            $depth--;
        }
        if ((($i == length($string)-1) && $depth > 0) || ($depth < 0))
        # Marker balance not OK
        {
            return 0;
        }
    }
    return 1;          # Marker balance OK
}

#--------------------------------------------------------------------------------------
-----#
# check_filogenetic_tree_format($FilogeneticTree, $StartRegex, $EndRegex,
$SeparatorRegex) #
#--------------------------------------------------------------------------------------
```

```
sub check_filogenetic_tree_format
{
if ($debug) {printf "check_filogenetic_tree_format\n";}
	# Read input parameters
	my $FilogeneticTree;
	my $StartRegex;
	my $EndRegex;
	my $SeparatorRegex;
	if ($#_==3) {$FilogeneticTree = $_[0]; $StartRegex = $_[1]; $EndRegex = $_[2];
$SeparatorRegex = $_[3];}
	else {die "\n!!!!!FATAL ERROR!!!!!\nWrong input arguments in function
\"check_filogenetic_tree_format\"$!";}

	# Check filogenetic tree format
	if(length($FilogeneticTree) && ($FilogeneticTree !~ m/$StartRegex/) &&
($FilogeneticTree !~ m/$EndRegex/) && ($FilogeneticTree !~ m/$SeparatorRegex/))
	{
		return 1;		# The tree has a single element
	}
	elsif(($FilogeneticTree =~ m/^$StartRegex/) && ($FilogeneticTree =~ m/$EndRegex$/)
		# The tree starts and ends with the correct markers
	&& check_marker_balance($FilogeneticTree, $StartRegex, $EndRegex))
					# The tree has a correct marker balance
	{
		$FilogeneticTree =~ s/$StartRegex//g;
		$FilogeneticTree =~ s/$EndRegex//g;
		if(($FilogeneticTree =~ m/$SeparatorRegex/)
							# The tree has separators
		&& ($FilogeneticTree !~ m/^$SeparatorRegex/)
						# The tree has text enclosing all separators
		&& ($FilogeneticTree !~ m/$SeparatorRegex$/)
		&& ($FilogeneticTree !~ m/[$SeparatorRegex]{2,}/))
		{
			return 1;	# The tree has correct markers, it has separators and all
separators are enclosed by text
		}
	}
	return 0;		# The tree has a bad format
}

#-------------------------------------------------------------------------------------------
----------#
# normalize_filogenetic_tree($FilogeneticTree, $outgroup, $StartRegex, $EndRegex,
$SeparatorRegex) #
#-------------------------------------------------------------------------------------------
----------#
sub normalize_filogenetic_tree
{
if ($debug) {printf "normalize_filogenetic_tree\n";}
	# Read input parameters
	my $FilogeneticTree;
	my $outgroup;
	my $StartRegex;
	my $EndRegex;
	my $SeparatorRegex;
	if ($#_==4) {$FilogeneticTree = $_[0]; $outgroup = $_[1]; $StartRegex = $_[2];
$EndRegex = $_[3]; $SeparatorRegex = $_[4];}
	else {die "\n!!!!!FATAL ERROR!!!!!\nWrong input arguments in function
\"normalize_filogenetic_tree\"$!";}

	# Check filogenetic tree format
	if(!check_filogenetic_tree_format($FilogeneticTree, $StartRegex, $EndRegex,
$SeparatorRegex))
```

```
        die "\n!!!!!FATAL ERROR!!!!!\nBad filogenetic tree format$!";
    }
    # Normalize filogenetic tree
    elsif(!($FilogeneticTree =~ m/$SeparatorRegex/))
                                    # There is only one element in the branch
    {
# printf "\$FilogeneticTree: $FilogeneticTree\n"; printf "\n";
        return $FilogeneticTree;
    }
    else
                                # There are several elements in the branch
    {
        $FilogeneticTree =~ s/^$StartRegex//;
        $FilogeneticTree =~ s/$EndRegex$//;
        my @branch;
        my $depth = 0;
        my $BranchNum = 0;
        for (my $i=0; $i<=length($FilogeneticTree); $i++)
                                    # Split tree branches
        {
            if((substr($FilogeneticTree, $i, 1) =~ m/$SeparatorRegex/) && ($depth ==
0))        # New branch found
            {
                $BranchNum++;
            }
            else
                                # Save character in branch
            {
                if(defined($branch[$BranchNum][0]))
                {
                    $branch[$BranchNum][0] .= substr($FilogeneticTree, $i, 1);
        # Concatenate string
                }
                else
                {
                    $branch[$BranchNum][0] = substr($FilogeneticTree, $i, 1);
        # Initialize string
                }
                if(substr($FilogeneticTree, $i, 1) =~ m/$StartRegex/)
                {
                    $depth++;
                        # Calculate depth
                }
                elsif(substr($FilogeneticTree, $i, 1) =~ m/$EndRegex/)
                {
                    $depth--;
                        # Calculate depth
                }
            }
        }
# printf "\$\#branch: $#branch\n"; print_2D_list(\@branch); printf "\n";
        for (my $i=0; $i<=$#branch; $i++)
                                        # Normalize branches
        {
            $branch[$i][0] = normalize_filogenetic_tree($branch[$i][0], $outgroup,
$StartRegex, $EndRegex, $SeparatorRegex);
            $branch[$i][1] = $branch[$i][0];
            $branch[$i][1] =~ s/$StartRegex//g;
            $branch[$i][1] =~ s/$EndRegex//g;
            $branch[$i][1] =~ s/$SeparatorRegex//g;
                                        # Clean copy of the branch (without start/end/
separator)
        }
```

```
# printf "\$\#branch: $#branch\nPrint 1D list\n"; print_1D_list(\@branch); printf "Print 
2D list\n"; print_2D_list(\@branch); printf "\n";
# printf "NOT ORDERED\n\$\#branch: $#branch\nPrint 2D list\n"; print_2D_list(\@branch); 
printf "\n";
          @branch = sort {$a->[1] cmp $b->[1]} @branch;
                                   # Sort branches by name
# printf "HALF ORDERED\n\$\#branch: $#branch\nPrint 2D list\n"; print_2D_list(\@branch); 
printf "\n";
          for(my $i=$#branch; $i>=1; $i--)
                                   # Move the outgroup to the first position
          {
               if($branch[$i][1] =~ m/$outgroup/)
               {
                    my @aux;
                    $aux[0]   = $branch[$i];
                    $branch[$i] = $branch[$i-1];
                    $branch[$i-1] = $aux[0];
               }
          }
# printf "\$\#branch: $#branch\nPrint 1D list\n"; print_1D_list(\@branch); printf "Print 
2D list\n"; print_2D_list(\@branch); printf "\n";
# printf "ORDERED\n\$\#branch: $#branch\nPrint 2D list\n"; print_2D_list(\@branch); 
printf "\n";
          @branch = select_column_in_2D_list(\@branch, 0);
                                   # Select sorted branches
          return regex_to_string($StartRegex).join(regex_to_string($SeparatorRegex),
@branch).regex_to_string($EndRegex);      # Join sorted branches
     }
}


###################
##  SUBPROGRAMS  ##
###################
#---------------------------------------------------------#
# select_complete_blocks(\@list, \@species, $ColSpecies) #
#---------------------------------------------------------#
sub select_complete_blocks
{
if ($debug) {printf "select_complete_blocks\n";}
     # Read input parameters
     my @list;
     my @species;
     my $ColSpecies;
     if ($#_==2) {@list = @{$_[0]}; @species = @{$_[1]}; $ColSpecies = $_[2];}
     else {die "\n!!!!!FATAL ERROR!!!!!\nWrong input arguments in function 
\"select_complete_blocks\"$!";}

     # Select complete blocks
     my $line = 0;
     my $col = 0;
     my @result;
     my $ResultLine = 0;
     while($line <= $#list-$#species)
     {
          # Initialize "@SpeciesUsed"
          my @SpeciesUsed;
          for(my $i=0; $i<=$#species; $i++)
          {
               $SpeciesUsed[$i] = 0;
          }
          # Count number of species present and not repeated
          my $NumberOfSpeciesUsed = 0;
          for (my $i=$line; $i<=$line+$#species; $i++)
```

```
            my $aux = find_index(\@species, (split(/\./, $list[$i][$ColSpecies]))[0]);
            if ($aux >= 0)
            {
                if (!$SpeciesUsed[$aux])         # Species not used yet
                {
                    $SpeciesUsed[$aux] = 1;
                    $NumberOfSpeciesUsed++;
                }
                else {last;}                     # Species already used
            }
            else {last;}                         # Species not present
        }
        # All species are present and not repeated --> Copy block
        if ($NumberOfSpeciesUsed == $#species+1)       # Copy block
        {
            for(my $i=0; $i<=$#species; $i++)
            {
                $result[$ResultLine] = $list[$line];
                $ResultLine++;
                $line++;
            }
        }
        else {$line++;}
        # Jump to the next line
    }
    return @result;
}

#---------------------------------------------------------------------------------#
# add_new_species(\@list, \@OldSpecies, \@NewSpecies, $ColSpecies, $RefSpecies) #
#---------------------------------------------------------------------------------#
sub add_new_species
{
if ($debug) {printf "add_new_species\n";}
    # Read input parameters
    my @list;
    my @OldSpecies;
    my @NewSpecies;
    my $ColSpecies;
    my $RefSpecies;
    if ($#_==4) {@list = @{$_[0]}; @OldSpecies = @{$_[1]}; @NewSpecies = @{$_[2]};
$ColSpecies = $_[3]; $RefSpecies = $_[4];}
    else {die "\n!!!!!FATAL ERROR!!!!!\nWrong input arguments in function
\"add_new_species\"$!";}

    # Add new species
    my $DefaultValue = "\.";
    my @CopyColFromRefSpecies = qw(0 2);
    my @result;
    my $line = 0;
    my $ResultLine = 0;
    while($line<=$#list-$#OldSpecies)
    {
        my $RefSpeciesIndex;
        for(my $i=0; $i<=$#OldSpecies; $i++)
                                    # Copy old species
        {
                $result[$ResultLine] = $list[$line];
                if((split(/\./, $list[$line][$ColSpecies]))[0] eq $RefSpecies)
                {
                    $RefSpeciesIndex = $line;
                                        # Save line index of the reference species in the
current block
```

```
				$ResultLine++;
				$line++;
			}
		for(my $i=0; $i<=$#NewSpecies; $i++)
							# Insert new species
		{
			for(my $j=0; $j<=$#{$list[$RefSpeciesIndex]}; $j++)
			{
				if($j==$ColSpecies)
								# Add specie name
				{
					$result[$ResultLine][$j] = $NewSpecies[$i];
				}
				elsif(find_index(\@CopyColFromRefSpecies, $j) >= 0)
					# Copy column from reference species data
				{
					$result[$ResultLine][$j] = $list[$RefSpeciesIndex][$j];
				}
				else
						# Fill column default value
				{
					$result[$ResultLine][$j] = $DefaultValue;
				}
			}
			$ResultLine++;
		}
	}
	return @result;
}

#-------------------------------------------------#
# sort_blocks(\@list, \@AllSpecies, $ColSpecies) #
#-------------------------------------------------#
sub sort_blocks
{
if ($debug) {printf "sort_blocks\n";}
	# Read input parameters
	my @list;
	my @AllSpecies;
	my $ColSpecies;
	if ($#_==2) {@list = @{$_[0]}; @AllSpecies = @{$_[1]}; $ColSpecies = $_[2];}
	else {die "\n!!!!!FATAL ERROR!!!!!\nWrong input arguments in function \"sort_blocks
\"$!";}

	# Sort blocks
	my $i=0;
	my @result;
	while($i<=$#list)
	{
		my @block = @list[$i..$i+$#AllSpecies];
									# Select block
		@block = sort_2D_list_as_reference_column(\@block, \@AllSpecies, $ColSpecies);
		# Sort block
		for (my $j=0; $j<=$#block; $j++)
								# Copy sorted block
		{
			$result[$i] = $block[$j];
			$i++;
		}
	}
	return @result;
}
```

```
#-----------------------------------------------------------------#
# trim_NEX_sequences(\@list, $ColSpecies, $ColSeq, $RefSpecies) #
#-----------------------------------------------------------------#
sub trim_NEX_sequences
{
if ($debug) {printf "trim_NEX_sequences\n";}
	# Read input parameters
	my @list;
	my $ColSpecies;
	my $ColSeq;
	my $RefSpecies;
	if ($#_==3) {@list = @{$_[0]}; $ColSpecies = $_[1]; $ColSeq = $_[2]; $RefSpecies =
$_[3];}
	else {die "\n!!!!!FATAL ERROR!!!!!\nWrong input arguments in function
\"trim_NEX_sequences\"$!";}

	# Trim sequences
	my @species;
	for (my $i=0; $i<=$#list; $i++)
	{
		$species[$i] = (split(/\./, $list[$i][$ColSpecies]))[0];
				# List of species in @list
	}
	# Trim '-' at the start of the sequence
	while(1)
	{
		my $RefSpeciesIndex = find_index(\@species, $RefSpecies);
		if ($RefSpeciesIndex >= 0)
		{
			if ($list[$RefSpeciesIndex][$ColSeq] =~ m/^-/)
							# If there is a '-' at the start of $RefSpecies
			{
				for (my $i=0; $i<=$#list; $i++)
				{
					if ($i == $RefSpeciesIndex) {$list[$i][$ColSeq] =~ s/^-//;}
		# Remove first '-' of $RefSpecies
					else {$list[$i][$ColSeq] =~ s/^.//;}
							# Remove first character in the rest of species
				}
			}
			else {last;}
							# If there are no more '-' at the start of $RefSpecies
--> End trim
		}
		else {die "\n!!!!!FATAL ERROR!!!!!\nReference species $RefSpecies not found in
list$!";}
	}
	# Trim '-' at the end of the sequence
	while(1)
	{
		my $RefSpeciesIndex = find_index(\@species, $RefSpecies);
		if ($RefSpeciesIndex >= 0)
		{
			if ($list[$RefSpeciesIndex][$ColSeq] =~ m/-$/)
							# If there is a '-' at the end of $RefSpecies
			{
				for (my $i=0; $i<=$#list; $i++)
				{
					if ($i == $RefSpeciesIndex) {$list[$i][$ColSeq] =~ s/-$//;}
		# Remove last '-' of $RefSpecies
					else {$list[$i][$ColSeq] =~ s/.$//;}
							# Remove last character in the rest of species
				}
			}
```

```
                                        # If there are no more '-' at the end of $RefSpecies -->
End trim
          }
          else {die "\n!!!!!FATAL ERROR!!!!!\nReference species $RefSpecies not found in
list$!";}
     }
     return @list;
}


#####################
##  PROGRAM START  ##
#####################
my @aux;
my @MAFfile = import_csv_file($MAFfilename, $MAFcolSeparator);
                                                       # Import "$MAFfilename"
@MAFfile = select_lines_in_2D_list(\@MAFfile, $MAFcolType, "s");
                                                  # Select lines starting with "s" (sequence
lines)
@MAFfile = select_complete_blocks(\@MAFfile, \@MAFspecies, $MAFcolSpecies);
                                        # Select complete blocks as indicated in "@MAFspecies"
@MAFfile = add_new_species(\@MAFfile, \@MAFspecies, \@NewSpecies, $MAFcolSpecies,
$RefSpecies);      # Add "@NewSpecies" to each block
@MAFfile = sort_blocks(\@MAFfile, \@AllSpecies, $MAFcolSpecies);
                                                  # Sort blocks as indicated in "@AllSpecies"

# Copy sequences from NEX file to MAF file
my $line=0;
while($line<=$#MAFfile)
{
if ($debug) {printf "Line ".($line+1)." of ".($#MAFfile+1)."\n";}
     my @MAFblock = @MAFfile[$line..$line+$#AllSpecies];
                                                       # Select MAF block
     my @MAFspecies;
                                             # List of species in MAFblock
     for (my $i=0; $i<=$#MAFblock; $i++)
     {
          $MAFspecies[$i] = (split(/\./, $MAFblock[$i][$MAFcolSpecies]))[0];
     }
     if(find_index(\@MAFspecies, $RefSpecies) >= 0)
     {
          my $filename = sprintf($NEXfilename, $MAFfile[$line+find_index(\@MAFspecies,
$RefSpecies)][$MAFcolPos]);
          printf "Processing file: \"$filename\"\n";
          my @NEXfile = import_csv_file($filename, $NEXcolSeparator);
                                             # Import NEX file
          @NEXfile = delete_empty_lines_in_2D_list(\@NEXfile);
                                                  # Delete empty lines
          @NEXfile = delete_character_in_2D_list(\@NEXfile, $NEXcolSpecies, qr/^>/);
                    # Delete '>' character from species name
          @NEXfile = trim_NEX_sequences(\@NEXfile, $NEXcolSpecies, $NEXcolSeq,
$RefSpecies);      # Trim '-' character at the start/end of the sequences
          my @NEXspecies;
                                             # List of species in NEXfile
          for (my $i=0; $i<=$#NEXfile; $i++)
          {
               $NEXspecies[$i] = (split(/\./, $NEXfile[$i][$NEXcolSpecies]))[0];
          }
          for(my $i=0; $i<=$#MAFblock; $i++)
                                                  # Copy sequences from NEX file to MAF file
          {
               if(find_index(\@NEXspecies, $MAFspecies[$i]) >= 0)
               {
```

```
                    $MAFfile[$line][$MAFcolSeq] = $NEXfile[find_index(\@NEXspecies,
$MAFspecies[$i])][$NEXcolSeq];
                   $line++;
              }
              else {die "\n!!!!!FATAL ERROR!!!!!\nSpecies \"".$MAFspecies[$i]."\" not
found in \"$filename\"$!";}
          }
          printf "File OK\n";
     }
     else { die "\n!!!!!FATAL ERROR!!!!!\nSpecies \"$RefSpecies\" not found in current
block$!";}
}
# Save result to file
print_2D_list_to_file(\@MAFfile, $OutFilename, "\t");

##############################PROGRAM END#####################

###############################################################
############# 3.- An. bwamabe sequence donwloading ############
###############################################################

#3.1.-Donwload sequences
module load SRA-Toolkit/2.8.1-3

fastq-dump --split-files SRR1255390 ## An. bwambae 3
fastq-dump --split-files SRR1255325 ## An. bwambae 4
fastq-dump --split-files SRR1255391 ## An. bwambae 1
fastq-dump --split-files SRR1255392 ## An. bwambae 1
fastq-dump --split-files SRR1255303 ## An. bwambae 1

cat SRR1255303_1.fastq SRR1255391_1.fastq SRR1255392_1.fastq > bwa1_1.fastq
cat SRR1255303_2.fastq SRR1255391_2.fastq SRR1255392_2.fastq > bwa1_2.fastq

###############################################################
############## 4.- An. bwamabe mapping to AgamP3 ##############
###############################################################
#4.1.-Illumina Read Quality Control
mkdir $out
mkdir $out/fastqc
perl fastqc $read1 $read2 -o $out/fastqc

#4.2.-Trimmed the reads
mkdir -p $out/trimmed
module load Python/2.7.9

cutadapt \
-b GATCGGAAGAGCACACGTCTGAACTCCAGTCACNNNNNNATCTCGTATGCCGTCTTCTGCTTG \
-B GATCGGAAGAGCACACGTCTGAACTCCAGTCACNNNNNNATCTCGTATGCCGTCTTCTGCTTG \
-b AATGATACGGCGACCACCGAGATCTACACTCTTTCCCTACACGACGCTCTTCCGATCT \
-B AATGATACGGCGACCACCGAGATCTACACTCTTTCCCTACACGACGCTCTTCCGATCT \
-O 15 \
-n 3 \
-q 18 \
--minimum-length 75 \
-o $out/trimmed/$name-1.fq.gz \
-p $out/trimmed/$name-2.fq.gz \
 $read1 \
 $read2

#4.3.-Trimmed Illumina Read Quality Control
mkdir -p $out/fastqc/trimmed
perl fastqc $out/trimmed/$name-1.fq.gz $out/trimmed/$name-2.fq.gz -o $out/fastqc/trimmed

#4.4.-Mapping with BWA-mem
```

```
module load BWA/0.7.15
mkdir $out/mapping

bwa index $ref/Anopheles-gambiae-PEST_CHROMOSOMES_AgamP3.fa

bwa mem -M $ref/Anopheles-gambiae-PEST_CHROMOSOMES_AgamP3.fa $name/trimmed/$name-1.fq.gz
$name/trimmed/$name-2.fq.gz | samtools view -Sbh -q 20 -F 0x100 - > $out/mapping/
$name.bam

#4.5.-Cleanning the alignment
java -Xmx20G -jar CleanSam.jar \
INPUT=$out/mapping/$name.bam \
OUTPUT=$out/mapping/$name-clean.bam \
VALIDATION_STRINGENCY=LENIENT

#4.6.-Sorting by reference
java -Xmx20G -jar SortSam.jar \
INPUT=$out/mapping/$name-clean.bam \
OUTPUT=$out/mapping/$name-sorted.bam \
SO=coordinate \
VALIDATION_STRINGENCY=SILENT

#4.7.- Mark Duplicates
mkdir $out/mapping/dedup-report
java -Xmx20g -Dsnappy.disable=true -jar MarkDuplicates.jar \
REMOVE_DUPLICATES=true \
I=$out/mapping/$name-sorted.bam \
O=$out/mapping/$name-dedup.bam \
M=$out/mapping/dedup-report/$name.txt \
VALIDATION_STRINGENCY=SILENT

#4.8.- Re-aling around indels
## a) create DICT file
java -jar CreateSequenceDictionary.jar \
REFERENCE=$ref/Anopheles-gambiae-PEST_CHROMOSOMES_AgamP3.fa \
OUTPUT=$ref/Anopheles-gambiae-PEST_CHROMOSOMES_AgamP3.dict

## b) create FASTA index file
samtools faidx $ref/Anopheles-gambiae-PEST_CHROMOSOMES_AgamP3.fa

## c) add read group to BAM files
module load picard/1.109
java -jar -Xmx10g AddOrReplaceReadGroups.jar \
INPUT=$out/mapping/$name-dedup.bam \
OUTPUT=$out/mapping/$name-dedup_rg.bam \
SORT_ORDER=coordinate \
RGID=$name \
RGLB=$name \
RGPL=illumina \
RGSM=sample \
RGPU=name \
CREATE_INDEX=true \
VALIDATION_STRINGENCY=SILENT

## d) remove BAM without ReadGroup
rm $out/mapping/$name-dedup.bam

## e)generate target List of InDel positions
mkdir $out/mapping/realign_list
module load GATK/3.4-46
java -jar GenomeAnalysisTK.jar \
-T RealignerTargetCreator \
-R $ref/Anopheles-gambiae-PEST_CHROMOSOMES_AgamP3.fa \
-I $out/mapping/$name-dedup_rg.bam \
```

```
-o $out/mapping/realign_list/$name.list

## f) re-align around InDels
java -Xmx20g -jar GenomeAnalysisTK.jar \
-T IndelRealigner \
-R $ref/Anopheles-gambiae-PEST_CHROMOSOMES_AgamP3.fa \
-I $out/mapping/$name-dedup_rg.bam \
-targetIntervals $out/mapping/realign_list/$name.list \
-o $out/mapping/$name-dedup_rg_InDel.bam


###############################################################
############## 5.- An. bwambae from fastq to MAF ##############
###############################################################

#5.1.- mpileup of each MAF region (in fastq format)
module load SAMtools/1.3.1
module load BCFtools/1.3.1

mkdir MAF_fragment_fasta

while read i
do
samtools view -b $name/mapping/$name-dedup_rg_InDel.bam "$i" > MAF_fragment_fasta/$name/
region-$i.bam
samtools mpileup -uf $ref/Anopheles-gambiae-PEST_CHROMOSOMES_AgamP3.fa
MAF_fragment_fasta/$name/region-$i.bam | bcftools call -c | vcfutils.pl vcf2fq >
MAF_fragment_fasta/$name/region-$i.fastq
done < MAF_fragment_coordinates.txt

#5.2.- mpileup of each MAF region (in fasta format)
#!/usr/bin/perl
use warnings;
use strict;

my $nucl = '';
my @nucl = '';
my $coor = '';

my $name = $ARGV[0];
my $chr = $ARGV[1];

open (COOR, "<MAF_fragment_coordinates.txt") or die "cannot read file";

while (<COOR>){
        $_ =~ s/\n//g;
        $coor = $_;
        open (FQ, "<MAF_fragment_fasta/$name/region-$coor.fastq") or die "cannot open
file";
        open (OUT, ">MAF_fragment_fasta/$name/region-$coor.fasta") or die "cannot
create";
        while(<FQ>){
                $_ =~ s/\n//g;
                if ($_ =~/^\@(.*)/){
                        print OUT ">$name\n";
                }elsif ($_ =~/^[natgcATCG].*/){
                        @nucl = split ("", $_);
                        foreach $nucl (@nucl){
                                if ($nucl =~/[ATCGRYMKSWHBVDNatgcrymkswhbvd]/){
                                        print OUT "$nucl";
                                }
                        }
                }
        }
```

```
###############################################################
########## 6.- Window-based phylogeny analysis ################
###############################################################

#6.1.- Fragmentate MAF in windows.
#!/usr/bin/perl
use warnings;
use strict;

my $i='';
my $s='';
my $seq_actual='';
my $seq_next='';
my $seq_actual_nf2='';
my $seq_next_nf2='';
my $seq_actual_nf3='';
my $seq_next_nf3='';
my $seq_actual_nf4='';
my $seq_next_nf4='';
my $seq_actual_nf5='';
my $seq_next_nf5='';
my $seq_actual_AgamS1='';
my $seq_next_AgamS1='';
my $seq_actual_AgamM1='';
my $seq_next_AgamM1='';
my $seq_actual_Amer='';
my $seq_next_Amer='';
my $seq_actual_Aara='';
my $seq_next_Aara='';
my $seq_actual_Aqua='';
my $seq_next_Aqua='';
my $seq_actual_Amel='';
my $seq_next_Amel='';
my $seq_actual_Achr='';
my $seq_next_Achr='';
my $seq_next_bwa1 = '';
my $seq_actual_bwa1 = '';
my $seq_next_bwa3 = '';
my $seq_actual_bwa3 = '';
my $seq_next_bwa4 = '';
my $seq_actual_bwa4 = '';
my $window='';
my $first_frag='';
my $last_frag ='';

for ($i=50000; $i<=24400000; $i= $i+50000){
     my $seq='';
     my @col='';
     my @AgamP3='';
     my @nf2='';
     my @nf3='';
     my @nf4 = '';
     my @nf5 = '';
     my @bwa1_new = '';
     my @bwa3_new = '';
     my @bwa4_new = '';
     my @AgamS1='';
     my @AgamM1='';
     my @Amer='';
     my @Aara='';
     my @Aqua='';
     my @Amel='';
```

my @Ach='·';

```
     $s = $i - 50000;
     print "$i\t$s\n";
     if ($window == 3){
          push (@AgamP3, $seq_next);
          push (@nf2, $seq_next_nf2);
          push (@nf3, $seq_next_nf3);
          push (@nf4, $seq_next_nf4);
          push (@nf5, $seq_next_nf5);
          push (@AgamS1, $seq_next_AgamS1);
          push (@AgamM1, $seq_next_AgamM1);
          push (@Amer, $seq_next_Amer);
          push (@Aara, $seq_next_Aara);
          push (@Aqua, $seq_next_Aqua);
          push (@Amel, $seq_next_Amel);
          push (@Achr, $seq_next_Achr);
          push (@bwa1_new, $seq_next_bwa1);
          push (@bwa3_new, $seq_next_bwa3);
          push (@bwa4_new, $seq_next_bwa4);
          $window='';
     }
     $window='';
     open (MAF, "<output_MAF/chrX.tba.aligned_fontenillei_4ind_bwa_3ind.maf") or die
"cannot open file";
     while(<MAF>){
     $_ =~ s/\n//g;
               if($_ =~ /^s.*/){
                    @col = split ("\t", $_);
                    if ($col[1] eq "AgamP3.chrX"){
                         $window=0;
                         $window++;
                         $seq_actual = '';
                         $seq_next = '';
                         my $pos = $col[2];
                         my $long = length $col[6];
                         my $region = $pos + $long;
                         if ($region > $i && $pos < $i){
                              $first_frag = $i - $pos;
                              $last_frag = $long - $first_frag;
                              $seq_actual = substr $col[6], 0, $first_frag;
                              $seq_next = substr $col[6], $first_frag, $last_frag;
                              $window=3;
                              print $window."\n";
                              push (@AgamP3, $seq_actual);
                         }elsif ($region <= $i && $region >= $s){
                              $window=2;
                              push (@AgamP3, $col[6]);
                         }
                    }elsif ($col[1] =~ /X_An_fontenillei_2nf_2nf/ && $window == 2){
                      push (@nf2, $col[6]);
                 }elsif ($col[1] =~ /X_An_fontenillei_2nf_2nf/ && $window == 3){
                      $seq_actual_nf2 = substr $col[6], 0, $first_frag;
                      $seq_next_nf2 = substr $col[6], $first_frag, $last_frag;
                      push (@nf2, $seq_actual_nf2);
                 }elsif ($col[1] =~ /X_An_fontenillei_3nf/ && $window == 2){
                      push (@nf3, $col[6]);
                 }elsif ($col[1] =~ /X_An_fontenillei_3nf/ && $window == 3){
                      $seq_actual_nf3 = substr $col[6], 0, $first_frag;
                      $seq_next_nf3 = substr $col[6], $first_frag, $last_frag;
                      push (@nf3, $seq_actual_nf3);
                 }elsif ($col[1] =~ /X_An_fontenillei_4nf/ && $window == 2){
                      push (@nf4, $col[6]);
                 }elsif ($col[1] =~ /X_An_fontenillei_4nf/ && $window == 3){
```

```
        $seq_actual_nf4 = substr $col[6], 0, $first_frag;
        $seq_next_nf4 = substr $col[6], $first_frag, $last_frag;
        push (@nf4, $seq_actual_nf4);
    }elsif ($col[1] =~ /X_An_fontenillei_5nf/ && $window == 2){
        push (@nf5, $col[6]);
    }elsif ($col[1] =~ /X_An_fontenillei_5nf/ && $window == 3){
        $seq_actual_nf5 = substr $col[6], 0, $first_frag;
        $seq_next_nf5 = substr $col[6], $first_frag, $last_frag;
        push (@nf5, $seq_actual_nf5);
       }elsif ($col[1] =~ /AgamS1.*/ && $window == 2){
           push (@AgamS1, $col[6]);
       }elsif ($col[1] =~ /AgamS1.*/ && $window == 3){
           $seq_actual_AgamS1 = substr $col[6], 0, $first_frag;
           $seq_next_AgamS1 = substr $col[6], $first_frag, $last_frag;
           push (@AgamS1, $seq_actual_AgamS1);
       }elsif ($col[1] =~ /AgamM1.*/ && $window == 2){
           push (@AgamM1, $col[6]);
       }elsif ($col[1] =~ /AgamM1.*/ && $window == 3){
           $seq_actual_AgamM1 = substr $col[6], 0, $first_frag;
           $seq_next_AgamM1 = substr $col[6], $first_frag, $last_frag;
           push (@AgamM1, $seq_actual_AgamM1);
       }elsif ($col[1] =~ /Amer.*/ && $window == 2){
           push (@Amer, $col[6]);
       }elsif ($col[1] =~ /Amer.*/ && $window == 3){
           $seq_actual_Amer = substr $col[6], 0, $first_frag;
           $seq_next_Amer = substr $col[6], $first_frag, $last_frag;
           push (@Amer, $seq_actual_Amer);
       }elsif ($col[1] =~ /Aara.*/ && $window == 2){
           push (@Aara, $col[6]);
       }elsif ($col[1] =~ /Aara.*/ && $window == 3){
           $seq_actual_Aara = substr $col[6], 0, $first_frag;
           $seq_next_Aara = substr $col[6], $first_frag, $last_frag;
           push (@Aara, $seq_actual_Aara);
       }elsif ($col[1] =~ /Aqua.*/ && $window == 2){
           push (@Aqua, $col[6]);
       }elsif ($col[1] =~ /Aqua.*/ && $window == 3){
           $seq_actual_Aqua = substr $col[6], 0, $first_frag;
           $seq_next_Aqua = substr $col[6], $first_frag, $last_frag;
           push (@Aqua, $seq_actual_Aqua);
       }elsif ($col[1] =~ /Amel.*/ && $window == 2){
           push (@Amel, $col[6]);
       }elsif ($col[1] =~ /Amel.*/ && $window == 3){
           $seq_actual_Amel = substr $col[6], 0, $first_frag;
           $seq_next_Amel = substr $col[6], $first_frag, $last_frag;
           push (@Amel, $seq_actual_Amel);
    }elsif ($col[1] =~ /bwa1_new/ && $window == 2){
        push (@bwa1_new, $col[6]);
    }elsif ($col[1] =~ /bwa1_new/ && $window == 3){
        $seq_actual_bwa1 = substr $col[6], 0, $first_frag;
        $seq_next_bwa1 = substr $col[6], $first_frag, $last_frag;
        push (@bwa1_new, $seq_actual_bwa1);
       }elsif ($col[1] =~ /bwa3_new/ && $window == 2){
        push (@bwa3_new, $col[6]);
    }elsif ($col[1] =~ /bwa3_new/ && $window == 3){
        $seq_actual_bwa3 = substr $col[6], 0, $first_frag;
        $seq_next_bwa3 = substr $col[6], $first_frag, $last_frag;
        push (@bwa3_new, $seq_actual_bwa3);
    }elsif ($col[1] =~ /bwa4_new/ && $window == 2){
        push (@bwa4_new, $col[6]);
    }elsif ($col[1] =~ /bwa4_new/ && $window == 3){
        $seq_actual_bwa4 = substr $col[6], 0, $first_frag;
        $seq_next_bwa4 = substr $col[6], $first_frag, $last_frag;
        push (@bwa4_new, $seq_actual_bwa4);
       }elsif ($col[1] =~ /Achr.*/ && $window == 2){
```

```
                                push (@Achr, $col[6]);
                        }elsif ($col[1] =~ /Achr.*/ && $window == 3){
                                $seq_actual_Achr = substr $col[6], 0, $first_frag;
                                $seq_next_Achr = substr $col[6], $first_frag, $last_frag;
                                push (@Achr, $seq_actual_Achr);
                        }
                }
        }
        open (OUT, ">chrX.tba.aligned_fontenillei_4ind_bwa_3ind.maf_1out_$i.fasta") or
die "Error summarys";
        print OUT ">An_fontenillei_2nf\n";
        foreach $seq (@nf2){
            print OUT $seq;
        }
        print OUT "\n";
                print OUT ">An_fontenillei_3nf\n";
                foreach $seq (@nf3){
                        print OUT $seq;
                }
        print OUT "\n";
                print OUT ">An_fontenillei_4nf\n";
                foreach $seq (@nf4){
                        print OUT $seq;
                }
        print OUT "\n";
                print OUT ">An_fontenillei_5nf\n";
                foreach $seq (@nf5){
                        print OUT $seq;
                }
        print OUT "\n";
        print OUT ">AgamS1\n";
        foreach $seq (@AgamS1){
            print OUT $seq;
        }
        print OUT "\n";
        print OUT ">AgamM1\n";
        foreach $seq (@AgamM1){
            print OUT $seq;
        }
        print OUT "\n";
        print OUT ">Amer\n";
        foreach $seq (@Amer){
            print OUT $seq;
        }
        print OUT "\n";
        print OUT ">Aara\n";
        foreach $seq (@Aara){
            print OUT $seq;
        }
        print OUT "\n";
        print OUT ">Aqua\n";
        foreach $seq (@Aqua){
            print OUT $seq;
        }
        print OUT "\n";
        print OUT ">Amel\n";
        foreach $seq (@Amel){
            print OUT $seq;
        }
        print OUT "\n";
        print OUT ">Abwa1\n";
        foreach $seq (@bwa1_new){
            print OUT $seq;
        }
```

```
        print OUT "\n";
        print OUT ">Abwa3\n";
        foreach $seq (@bwa3_new){
            print OUT $seq;
        }
        print OUT "\n";
        print OUT ">Abwa4\n";
        foreach $seq (@bwa4_new){
            print OUT $seq;
        }
        print OUT "\n";
        print OUT ">Achr\n";
        foreach $seq (@Achr){
            print OUT $seq;
        }
        print OUT "\n";
    }

#6.2.- Run RAxML by window
#!/bin/bash -l
module load RAxML/8.2.4

echo -e 'Starting raxml' ${name} 'at' `date`'.\n\n'

while read i <&3
do
raxmlHPC-SSE3 -m GTRGAMMA -p 300 -x 43567 -s
tba_HD_AgamC9_fontenilleiAssembly_bwa1.maf_1out_$i.fasta -n
tba_HD_AgamC9_fontenilleiAssembly_bwa1.maf_1out_1000_$i.out -f a -# 1000 -o Achr
done 3< filtered_windows.txt

#6.3.- Phylogenetic tree topology sorting and counting
#!/usr/bin/perl
use warnings;
use strict;


#################
##  CONSTANTS  ##
#################
# Debug flags
my $debug = 0;
my $verbose = 0;

# Input file
my $InputFilename = "tree_by_window_assembly_1000.newick";
my $ColSeparator = "\t";                  # Column separator
my $ColTree = 0;                          # Column index for the filogenetic tree
my $outgroup = qr/O/;                     # Outgroup name regular expression
my $BranchStart = qr/\(/;                 # Branch start regular expression
my $BranchEnd = qr/\)/;                   # Branch end regular expression
my $BranchSeparator = qr/\,/;             # Branch separator regular expression

# Output file
my $OutputFilename = "tree_by_window_assembly_1000_sorted.newick";

###################
##  SUBROUTINES  ##
###################
#-----------------------------------------------------#
# import_csv_file($filename, $ColumnSeparator:"\t") #
#-----------------------------------------------------#
sub import_csv_file
{
```

```
if ($debug) {printf "import_csv_file\n";}
     # Read input parameters
     my $filename;
     my $ColumnSeparator = "\t";
     if ($#_==0) {$filename = $_[0];}
     elsif ($#_==1) {$filename = $_[0]; $ColumnSeparator = $_[1];}
     else {die "\n!!!!!FATAL ERROR!!!!!\nWrong input arguments in function
\"import_csv_file\"$!";}

     # Read input file
     my $InputFile;
     open ($InputFile, "<", $filename) or die "\n!!!!!FATAL ERROR!!!!!\nCan't open file
\"$filename\"$!";
     my @ResultAux = <$InputFile>;
     close $InputFile;

     # Build 2D list
     my @result;
     for (my $i=0; $i<=$#ResultAux; $i++)
     {
          $ResultAux[$i] =~ s/\n//g;
                    # Delete end of line characters
          my @aux = split("$ColumnSeparator", $ResultAux[$i]);
          # Separate columns by "tabs"
          for (my $j=0; $j<=$#aux; $j++)
          {
               $result[$i][$j] = $aux[$j];
          }
     }
     return @result;
}

#----------------------------#
# find_index(\@list, $text) #
#----------------------------#
sub find_index
{
if ($debug && $verbose) {printf "find_index\n";}
     # Read input parameters
     my @list;
     my $text;
     if ($#_==1) {@list = @{$_[0]}; $text = $_[1];}
     else {die "\n!!!!!FATAL ERROR!!!!!\nWrong input arguments in function \"find_index
\"$!";}

     use List::Util qw(first);
     my $result = first {$list[$_] eq $text} 0..$#list;
     if (defined($result))
     {
          return $result
     }
     else
     {
          return -1
     }
}

#----------------------------------------#
# delete_empty_lines_in_2D_list(\@list) #
#----------------------------------------#
sub delete_empty_lines_in_2D_list
{
if ($debug) {printf "delete_empty_lines_in_2D_list\n";}
     # Read input parameters
```

```
	my @list;
	if ($#_==0) {@list = @{$_[0]};}
	else {die "\n!!!!!FATAL ERROR!!!!!\nWrong input arguments in function 
\"delete_empty_lines_in_2D_list\"$!";}

	# Delete empty lines
	my @result;
	my $line=0;
	for(my $i=0; $i<=$#list; $i++)
	{
		if($list[$i])
		{
			$result[$line] = $list[$i];
			$line++;
		}
	}
	return @result;
}

#-------------------------------------------------#
# delete_column_in_2D_list(\@list, $ColumnIndex) #
#-------------------------------------------------#
sub delete_column_in_2D_list
{
if ($debug) {printf "delete_column_in_2D_list\n";}
	# Read input parameters
	my @list;
	my $ColumnIndex;
	if ($#_==1) {@list = @{$_[0]}; $ColumnIndex = $_[1];}
	else {die "\n!!!!!FATAL ERROR!!!!!\nWrong input arguments in function 
\"delete_column_in_2D_list\"$!";}

	# Delete column
	my @result;
	for(my $i=0; $i<=$#list; $i++)
	{
		my $col=0;
		for(my $j=0; $j<=$#{$list[$i]}; $j++)
		{
			if($j != $ColumnIndex)
			{
				$result[$i][$col] = $list[$i][$j];
				$col++;
			}
		}
	}
	return @result;
}

#-----------------------------------------------------------------#
# sort_2D_list_by_column(\@list, $ColumnIndex, $mode: "text") #
#-----------------------------------------------------------------#
sub sort_2D_list_by_column
{
if ($debug) {printf "sort_2D_list_by_column\n";}
	# Read input parameters
	my @list;
	my $ColumnIndex;
	my $mode = "text";
	if ($#_==1) {@list = @{$_[0]}; $ColumnIndex = $_[1];}
	elsif ($#_==2) {@list = @{$_[0]}; $ColumnIndex = $_[1]; $mode = $_[2];}
	else {die "\n!!!!!FATAL ERROR!!!!!\nWrong input arguments in function 
\"sort_2D_list_by_column\"$!";}
```

```
     # sort 2D list by column
     if ($mode eq "text") {return sort {$a->[$ColumnIndex] cmp $b->[$ColumnIndex]}
@list;}
     elsif ($mode eq "number") {return sort {$a->[$ColumnIndex] <=> $b->[$ColumnIndex]}
@list;}
     else {die "\n!!!!!FATAL ERROR!!!!!\nWrong input mode in function
\"sort_2D_list_by_column\"$!\nSupported modes \"test\" & \"number\"";}
}

#---------------------------------------------------------------------------#
# sort_2D_list_as_reference_column(\@list, \@ReferenceColumn, $ColumnIndex) #
#---------------------------------------------------------------------------#
sub sort_2D_list_as_reference_column
{
if ($debug && $verbose) {printf "sort_2D_list_as_reference_column\n";}
     # Read input parameters
     my @list;
     my @ReferenceColumn;
     my $ColumnIndex;
     if ($#_==2) {@list = @{$_[0]}; @ReferenceColumn = @{$_[1]}; $ColumnIndex = $_[2];}
     else {die "\n!!!!!FATAL ERROR!!!!!\nWrong input arguments in function
\"sort_2D_list_as_reference_column\"$!";}

     # Sort 2D list as indicated by @ReferenceColumn
     return sort {find_index(\@ReferenceColumn, (split(/\./, $a->[$ColumnIndex]))[0]) <=>
find_index(\@ReferenceColumn, (split(/\./, $b->[$ColumnIndex]))[0])} @list;
}

#--------------------------------------------------------#
# select_lines_in_2D_list(\@list, $ColumnIndex, $text) #
#--------------------------------------------------------#
sub select_lines_in_2D_list
{
if ($debug) {printf "select_lines_in_2D_list\n";}
     # Read input parameters
     my @list;
     my $ColumnIndex;
     my $text;
     if ($#_==2) {@list = @{$_[0]}; $ColumnIndex = $_[1]; $text = $_[2];}
     else {die "\n!!!!!FATAL ERROR!!!!!\nWrong input arguments in function
\"select_lines_in_2D_list\"$!";}

     # Select lines
     my @result;
     my $j=0;
     for (my $i=0; $i<=$#list; $i++)
     {
          if (defined($list[$i][$ColumnIndex]) && ($list[$i][$ColumnIndex] eq $text))
          {
               $result[$j] = $list[$i];
               $j++;
          }
     }
     return @result;
}

#--------------------------------------------------#
# select_column_in_2D_list(\@list, $ColumnIndex) #
#--------------------------------------------------#
sub select_column_in_2D_list
{
if ($debug) {printf "select_column_in_2D_list\n";}
     # Read input parameters
     my @list;
```

```
    my $ColumnIndex;
    if ($#_==1) {@list = @{$_[0]}; $ColumnIndex = $_[1]}
    else {die "\n!!!!!FATAL ERROR!!!!!\nWrong input arguments in function
\"select_column_in_2D_list\"$!";}

    # Select column
    my @result;
    for (my $i=0; $i<=$#list; $i++)
    {
        if (defined($list[$i][$ColumnIndex]))
        {
            $result[$i] = $list[$i][$ColumnIndex];
        }
    }
    return @result;
}

#-------------------------------------------------------------#
# delete_character_in_2D_list(\@list, $ColumnIndex, $regex) #
#-------------------------------------------------------------#
sub delete_character_in_2D_list
{
if ($debug) {printf "delete_character_in_2D_list\n";}
    # Read input parameters
    my @list;
    my $ColumnIndex;
    my $regex;
    if ($#_==2) {@list = @{$_[0]}; $ColumnIndex = $_[1]; $regex = $_[2];}
    else {die "\n!!!!!FATAL ERROR!!!!!\nWrong input arguments in function
\"delete_character_in_2D_list\"$!";}

    for (my $i=0; $i<=$#list; $i++)

    {
        $list[$i][$ColumnIndex] =~ s/$regex//;
    }
    return @list;
}

#-------------------------------------------#
# print_1D_list(\@list, $ColumnSeparator) #
#-------------------------------------------#
sub print_1D_list
{
if ($debug) {printf "print_1D_list\n";}
    # Read input parameters
    my @list;
    my $ColumnSeparator = "\t";
    if ($#_==0) {@list = @{$_[0]};}
    elsif ($#_==1) {@list = @{$_[0]}; $ColumnSeparator = $_[1];}
    else {die "\n!!!!!FATAL ERROR!!!!!\nWrong input arguments in function
\"print_1D_list\"$!";}

    # Print 1D list
    for (my $i=0; $i<=$#list; $i++)
    {
        printf "$list[$i]";
        if ($i<$#list)
        {
            printf "$ColumnSeparator";
        }
    }
    printf "\n";
    return
```

```
#----------------------------------------------#
# print_2D_list(\@list, $ColumnSeparator) #
#----------------------------------------------#
sub print_2D_list
{
if ($debug) {printf "print_2D_list\n";}
     # Read input parameters
     my @list;
     my $ColumnSeparator = "\t";
     if ($#_==0) {@list = @{$_[0]};}
     elsif ($#_==1) {@list = @{$_[0]}; $ColumnSeparator = $_[1];}
     else {die "\n!!!!!FATAL ERROR!!!!!\nWrong input arguments in function
\"print_2D_list\"$!";}

     # Print 2D list
     for (my $i=0; $i<=$#list; $i++)
     {
          if ($list[$i]) {print_1D_list($list[$i], $ColumnSeparator);}
          else {printf "\n";}
     }
     return
}

#------------------------------------------------------------#
# print_1D_list_to_file(\@list, $filename, $Separator) #
#------------------------------------------------------------#
sub print_1D_list_to_file
{
if ($debug) {printf "print_1D_list_to_file\n";}
     # Read input parameters
     my @list;
     my $filename;
     my $ColumnSeparator = "\n";
     if ($#_==1) {@list = @{$_[0]}; $filename = $_[1];}
     elsif ($#_==2) {@list = @{$_[0]}; $filename = $_[1]; $ColumnSeparator = $_[2];}
     else {die "\n!!!!!FATAL ERROR!!!!!\nWrong input arguments in function
\"print_1D_list_to_file\"$!";}

     # Print 1D list
     open (OUT, ">", $filename) or die "\n!!!!!FATAL ERROR!!!!!\nCan't open file
\"$filename\"$!";
     for (my $i=0; $i<=$#list; $i++)
     {
          if ($list[$i])
          {
               printf OUT "$list[$i]";
          }
          if ($i<$#list)
          {
               printf OUT "$ColumnSeparator";
          }
     }
     close OUT;
     return
}

#------------------------------------------------------------------#
# print_2D_list_to_file(\@list, $filename, $ColumnSeparator) #
#------------------------------------------------------------------#
sub print_2D_list_to_file
{
if ($debug) {printf "print_2D_list_to_file\n";}
```

```
    # Read input parameters
    my @list;
    my $filename;
    my $ColumnSeparator = "\t";
    if ($#_==1) {@list = @{$_[0]}; $filename = $_[1];}
    elsif ($#_==2) {@list = @{$_[0]}; $filename = $_[1]; $ColumnSeparator = $_[2];}
    else {die "\n!!!!!FATAL ERROR!!!!!\nWrong input arguments in function
\"print_2D_list_to_file\"$!";}

    # Print 2D list
    open (OUT, ">", $filename) or die "\n!!!!!FATAL ERROR!!!!!\nCan't open file
\"$filename\"$!";
    for (my $i=0; $i<=$#list; $i++)
    {
        if ($list[$i])
        {
            for (my $j=0; $j<=$#{$list[$i]}; $j++)
            {
                printf OUT "$list[$i][$j]";
                if ($j<$#{$list[$i]})
                {
                    printf OUT "$ColumnSeparator";
                }
            }
        }
        printf OUT "\n"
    }
    close OUT;
    return
}

#-------------------------#
# regex_to_string($regex) #
#-------------------------#
sub regex_to_string
{
if ($debug) {printf "regex_to_string\n";}
    # Read input parameters
    my $regex;
    if ($#_==0) {$regex = $_[0];}
    else {die "\n!!!!!FATAL ERROR!!!!!\nWrong input arguments in function
\"regex_to_string\"$!";}

    # Convert regex to string
    my $result = sprintf($regex);
    $result =~ s/($regex)//;
    return $1;
}

#-------------------------------------------------------------#
# check_marker_balance($string, $StartRegex, $EndRegex) #
#-------------------------------------------------------------#
sub check_marker_balance
{
if ($debug) {printf "check_marker_balance\n";}
    # Read input parameters
    my $string;
    my $StartRegex;
    my $EndRegex;
    if ($#_==2) {$string = $_[0]; $StartRegex = $_[1]; $EndRegex = $_[2];}
    else {die "\n!!!!!FATAL ERROR!!!!!\nWrong input arguments in function
\"check_marker_balance\"$!";}

    # Check marker balance
```

```
my $depth = 0;
     for (my $i=0; $i<length($string); $i++)
     {
          if (substr($string, $i, 1) =~ m/$StartRegex/)
          {
               $depth++;
          }
          elsif (substr($string, $i, 1) =~ m/$EndRegex/)
          {
               $depth--;
          }
          if ((($i == length($string)-1) && $depth > 0) || ($depth < 0))
          # Marker balance not OK
          {
               return 0;
          }
     }
     return 1;           # Marker balance OK
}

#---------------------------------------------------------------------------------------------
-----#
# check_filogenetic_tree_format($FilogeneticTree, $StartRegex, $EndRegex,
$SeparatorRegex) #
#---------------------------------------------------------------------------------------------
-----#
sub check_filogenetic_tree_format
{
if ($debug) {printf "check_filogenetic_tree_format\n";}
     # Read input parameters
     my $FilogeneticTree;
     my $StartRegex;
     my $EndRegex;
     my $SeparatorRegex;
     if ($#_==3) {$FilogeneticTree = $_[0]; $StartRegex = $_[1]; $EndRegex = $_[2];
$SeparatorRegex = $_[3];}
     else {die "\n!!!!!FATAL ERROR!!!!!\nWrong input arguments in function
\"check_filogenetic_tree_format\"$!";}

     # Check filogenetic tree format
     if(length($FilogeneticTree) && ($FilogeneticTree !~ m/$StartRegex/) &&
($FilogeneticTree !~ m/$EndRegex/) && ($FilogeneticTree !~ m/$SeparatorRegex/))
     {
          return 1;          # The tree has a single element
     }
     elsif(($FilogeneticTree =~ m/^$StartRegex/) && ($FilogeneticTree =~ m/$EndRegex$/)
          # The tree starts and ends with the correct markers
     && check_marker_balance($FilogeneticTree, $StartRegex, $EndRegex))
                              # The tree has a correct marker balance
     {
          $FilogeneticTree =~ s/$StartRegex//g;
          $FilogeneticTree =~ s/$EndRegex//g;
          if(($FilogeneticTree =~ m/$SeparatorRegex/)
                                                      # The tree has separators
          && ($FilogeneticTree !~ m/^$SeparatorRegex/)
                                                # The tree has text enclosing all separators
          && ($FilogeneticTree !~ m/$SeparatorRegex$/)
          && ($FilogeneticTree !~ m/[$SeparatorRegex]{2,}/))
          {
               return 1;     # The tree has correct markers, it has separators and all
separators are enclosed by text
          }
     }
     return 0;               # The tree has a bad format
```

```
#-------------------------------------------------------------------------------------------
----------#
# normalize_filogenetic_tree($FilogeneticTree, $outgroup, $StartRegex, $EndRegex,
$SeparatorRegex) #
#-------------------------------------------------------------------------------------------
----------#
sub normalize_filogenetic_tree
{
if ($debug) {printf "normalize_filogenetic_tree\n";}
     # Read input parameters
     my $FilogeneticTree;
     my $outgroup;
     my $StartRegex;
     my $EndRegex;
     my $SeparatorRegex;
     if ($#_==4) {$FilogeneticTree = $_[0]; $outgroup = $_[1]; $StartRegex = $_[2];
$EndRegex = $_[3]; $SeparatorRegex = $_[4];}
     else {die "\n!!!!!FATAL ERROR!!!!!\nWrong input arguments in function
\"normalize_filogenetic_tree\"$!";}

     # Check filogenetic tree format
     if(!check_filogenetic_tree_format($FilogeneticTree, $StartRegex, $EndRegex,
$SeparatorRegex))
     {
          print "$FilogeneticTree";
          die "\n!!!!!FATAL ERROR!!!!!\nBad filogenetic tree format$!";
     }
     # Normalize filogenetic tree
     elsif(!($FilogeneticTree =~ m/$SeparatorRegex/))
                                   # There is only one element in the branch
     {
# printf "\$FilogeneticTree: $FilogeneticTree\n"; printf "\n";
          return $FilogeneticTree;
     }
     else
                                        # There are several elements in the branch
     {
          $FilogeneticTree =~ s/^$StartRegex//;
          $FilogeneticTree =~ s/$EndRegex$//;
          my @branch;
          my $depth = 0;
          my $BranchNum = 0;
          for (my $i=0; $i<=length($FilogeneticTree); $i++)
                                   # Split tree branches
          {
               if((substr($FilogeneticTree, $i, 1) =~ m/$SeparatorRegex/) && ($depth ==
0))       # New branch found
               {
                    $BranchNum++;
               }
               else
                                        # Save character in branch
               {
                    if(defined($branch[$BranchNum][0]))
                    {
                         $branch[$BranchNum][0] .= substr($FilogeneticTree, $i, 1);
          # Concatenate string
                    }
                    else
                    {
                         $branch[$BranchNum][0] = substr($FilogeneticTree, $i, 1);
          # Initialize string
```

```
                    if(substr($FilogeneticTree, $i, 1) =~ m/$StartRegex/)
                    {
                        $depth++;
                                    # Calculate depth
                    }
                    elsif(substr($FilogeneticTree, $i, 1) =~ m/$EndRegex/)
                    {
                        $depth--;
                                    # Calculate depth
                    }
                }
            }
# printf "\$\#branch: $#branch\n"; print_2D_list(\@branch); printf "\n";
            for (my $i=0; $i<=$#branch; $i++)
                                    # Normalize branches
            {
                $branch[$i][0] = normalize_filogenetic_tree($branch[$i][0], $outgroup,
$StartRegex, $EndRegex, $SeparatorRegex);
                $branch[$i][1] = $branch[$i][0];
                $branch[$i][1] =~ s/$StartRegex//g;
                $branch[$i][1] =~ s/$EndRegex//g;
                $branch[$i][1] =~ s/$SeparatorRegex//g;
                                    # Clean copy of the branch (without start/end/
separator)
            }
# printf "\$\#branch: $#branch\nPrint 1D list\n"; print_1D_list(\@branch); printf "Print
2D list\n"; print_2D_list(\@branch); printf "\n";
# printf "NOT ORDERED\n\$\#branch: $#branch\nPrint 2D list\n"; print_2D_list(\@branch);
printf "\n";
            @branch = sort {$a->[1] cmp $b->[1]} @branch;
                                # Sort branches by name
# printf "HALF ORDERED\n\$\#branch: $#branch\nPrint 2D list\n"; print_2D_list(\@branch);
printf "\n";
            for(my $i=$#branch; $i>=1; $i--)
                                # Move the outgroup to the first position
            {
                if($branch[$i][1] =~ m/$outgroup/)
                {
                    my @aux;
                    $aux[0]   = $branch[$i];
                    $branch[$i] = $branch[$i-1];
                    $branch[$i-1] = $aux[0];
                }
            }
# printf "\$\#branch: $#branch\nPrint 1D list\n"; print_1D_list(\@branch); printf "Print
2D list\n"; print_2D_list(\@branch); printf "\n";
# printf "ORDERED\n\$\#branch: $#branch\nPrint 2D list\n"; print_2D_list(\@branch);
printf "\n";
            @branch = select_column_in_2D_list(\@branch, 0);
                                # Select sorted branches
            return regex_to_string($StartRegex).join(regex_to_string($SeparatorRegex),
@branch).regex_to_string($EndRegex);      # Join sorted branches
        }
}


###################
##  SUBPROGRAMS  ##
###################
#---------------------------------------------------------#
# select_complete_blocks(\@list, \@species, $ColSpecies) #
#---------------------------------------------------------#
sub select_complete_blocks
```

```
if ($debug) {printf "select_complete_blocks\n";}
     # Read input parameters
     my @list;
     my @species;
     my $ColSpecies;
     if ($#_==2) {@list = @{$_[0]}; @species = @{$_[1]}; $ColSpecies = $_[2];}
     else {die "\n!!!!!FATAL ERROR!!!!!\nWrong input arguments in function
\"select_complete_blocks\"$!";}

     # Select complete blocks
     my $line = 0;
     my $col = 0;
     my @result;
     my $ResultLine = 0;
     while($line <= $#list-$#species)
     {
          # Initialize "@SpeciesUsed"
          my @SpeciesUsed;
          for(my $i=0; $i<=$#species; $i++)
          {
               $SpeciesUsed[$i] = 0;
          }
          # Count number of species present and not repeated
          my $NumberOfSpeciesUsed = 0;
          for (my $i=$line; $i<=$line+$#species; $i++)
          {
               my $aux = find_index(\@species, (split(/\./, $list[$i][$ColSpecies]))[0]);
               if ($aux >= 0)
               {
                    if (!$SpeciesUsed[$aux])         # Species not used yet
                    {
                         $SpeciesUsed[$aux] = 1;
                         $NumberOfSpeciesUsed++;
                    }
                    else {last;}                     # Species already used
               }
               else {last;}                          # Species not present
          }
          # All species are present and not repeated --> Copy block
          if ($NumberOfSpeciesUsed == $#species+1)       # Copy block
          {
               for(my $i=0; $i<=$#species; $i++)
               {
                    $result[$ResultLine] = $list[$line];
                    $ResultLine++;
                    $line++;
               }
          }
          else {$line++;}
          # Jump to the next line
     }
     return @result;
}

#---------------------------------------------------------------------------------#
# add_new_species(\@list, \@OldSpecies, \@NewSpecies, $ColSpecies, $RefSpecies) #
#---------------------------------------------------------------------------------#
sub add_new_species
{
if ($debug) {printf "add_new_species\n";}
     # Read input parameters
     my @list;
     my @OldSpecies;
```

```
    my @NewSpecies;
    my $ColSpecies;
    my $RefSpecies;
    if ($#_==4) {@list = @{$_[0]}; @OldSpecies = @{$_[1]}; @NewSpecies = @{$_[2]};
$ColSpecies = $_[3]; $RefSpecies = $_[4];}
    else {die "\n!!!!!FATAL ERROR!!!!!\nWrong input arguments in function
\"add_new_species\"$!";}

    # Add new species
    my $DefaultValue = "\.";
    my @CopyColFromRefSpecies = qw(0 2);
    my @result;
    my $line = 0;
    my $ResultLine = 0;
    while($line<=$#list-$#OldSpecies)
    {
        my $RefSpeciesIndex;
        for(my $i=0; $i<=$#OldSpecies; $i++)
                                # Copy old species
        {
                $result[$ResultLine] = $list[$line];
                if((split(/\./, $list[$line][$ColSpecies]))[0] eq $RefSpecies)
                {
                    $RefSpeciesIndex = $line;
                                    # Save line index of the reference species in the
current block
                }
                $ResultLine++;
                $line++;
        }
        for(my $i=0; $i<=$#NewSpecies; $i++)
                                # Insert new species
        {
            for(my $j=0; $j<=$#{$list[$RefSpeciesIndex]}; $j++)
            {
                if($j==$ColSpecies)
                                        # Add specie name
                {
                    $result[$ResultLine][$j] = $NewSpecies[$i];
                }
                elsif(find_index(\@CopyColFromRefSpecies, $j) >= 0)
                    # Copy column from reference species data
                {
                    $result[$ResultLine][$j] = $list[$RefSpeciesIndex][$j];
                }
                else
                                    # Fill column default value
                {
                    $result[$ResultLine][$j] = $DefaultValue;
                }
            }
            $ResultLine++;
        }
    }
    return @result;
}

#-------------------------------------------------#
# sort_blocks(\@list, \@AllSpecies, $ColSpecies) #
#-------------------------------------------------#
sub sort_blocks
{
if ($debug) {printf "sort_blocks\n";}
    # Read input parameters
```

```
    my @list;
    my @AllSpecies;
    my $ColSpecies;
    if ($#_==2) {@list = @{$_[0]}; @AllSpecies = @{$_[1]}; $ColSpecies = $_[2];}
    else {die "\n!!!!!FATAL ERROR!!!!!\nWrong input arguments in function \"sort_blocks
\"$!";}

    # Sort blocks
    my $i=0;
    my @result;
    while($i<=$#list)
    {
        my @block = @list[$i..$i+$#AllSpecies];
                                                    # Select block
        @block = sort_2D_list_as_reference_column(\@block, \@AllSpecies, $ColSpecies);
        # Sort block
        for (my $j=0; $j<=$#block; $j++)
                                               # Copy sorted block
        {
            $result[$i] = $block[$j];
            $i++;
        }
    }
    return @result;
}

#----------------------------------------------------------------#
# trim_NEX_sequences(\@list, $ColSpecies, $ColSeq, $RefSpecies) #
#----------------------------------------------------------------#
sub trim_NEX_sequences
{
if ($debug) {printf "trim_NEX_sequences\n";}
    # Read input parameters
    my @list;
    my $ColSpecies;
    my $ColSeq;
    my $RefSpecies;
    if ($#_==3) {@list = @{$_[0]}; $ColSpecies = $_[1]; $ColSeq = $_[2]; $RefSpecies =
$_[3];}
    else {die "\n!!!!!FATAL ERROR!!!!!\nWrong input arguments in function
\"trim_NEX_sequences\"$!";}

    # Trim sequences
    my @species;
    for (my $i=0; $i<=$#list; $i++)
    {
        $species[$i] = (split(/\./, $list[$i][$ColSpecies]))[0];
                  # List of species in @list
    }
    # Trim '-' at the start of the sequence
    while(1)
    {
        my $RefSpeciesIndex = find_index(\@species, $RefSpecies);
        if ($list[$RefSpeciesIndex][$ColSeq] =~ m/^-/)
                              # If there is a '-' at the start of $RefSpecies
        {
            for (my $i=0; $i<=$#list; $i++)
            {
                if ($i == $RefSpeciesIndex) {$list[$i][$ColSeq] =~ s/^-//;}
        # Remove first '-' of $RefSpecies
                else {$list[$i][$ColSeq] =~ s/^.//;}
                              # Remove first character in the rest of species
            }
        }
```

```
        else {last;}
                                        # If there are no more '-' at the start of $RefSpecies
--> End trim
    }
    # Trim '-' at the end of the sequence
    while(1)
    {
        my $RefSpeciesIndex = find_index(\@species, $RefSpecies);
        if ($list[$RefSpeciesIndex][$ColSeq] =~ m/-$/)
                                # If there is a '-' at the end of $RefSpecies
        {
            for (my $i=0; $i<=$#list; $i++)
            {
                if ($i == $RefSpeciesIndex) {$list[$i][$ColSeq] =~ s/-$//;}
        # Remove last '-' of $RefSpecies
                else {$list[$i][$ColSeq] =~ s/.$//;}
                                # Remove last character in the rest of species
            }
        }
        else {last;}
                                        # If there are no more '-' at the end of $RefSpecies -->
End trim
    }
    return @list;
}


#####################
##  PROGRAM START  ##
#####################
my @TreeList = import_csv_file($InputFilename, $ColSeparator);
        # Import input file
@TreeList = delete_empty_lines_in_2D_list(\@TreeList);
            # Delete empty lines
@TreeList = select_column_in_2D_list(\@TreeList, $ColTree);
            # Select filogenetic trees
for (my $i=0; $i<=$#TreeList; $i++)
{
    $TreeList[$i] = normalize_filogenetic_tree($TreeList[$i], $outgroup, $BranchStart,
$BranchEnd, $BranchSeparator);
}

print_1D_list_to_file(\@TreeList, "Unsorted_$OutputFilename");
@TreeList = sort(@TreeList);
                # Sort filogenetic tree list
my @result;
my $i = 0;
my $prev_i = 0;
my $j = 0;
my $count = 0;
while($i<=$#TreeList)
                # Count and print filogenetic tree list
{
    if($TreeList[$i] eq $TreeList[$prev_i])
                # If the trees are equal --> Count+1
    {
        $count++;
    }
    else
                # If the trees are different --> Save count
    {
        $result[$j][0] = $TreeList[$prev_i];
        $result[$j][1] = $count;
        $j++;
```

```
          $count = 1;
     }
     if($i == $#TreeList)
                    # If we reached the end of the list --> Save count
     {
          $result[$j][0] = $TreeList[$i];
          $result[$j][1] = $count;
     }
     $prev_i = $i;
     $i++;
}
@result = sort_2D_list_by_column(\@result, 1, "number");
print_2D_list_to_file(\@result, $OutputFilename);
               # Save result to disk


################################################################
#### 7.- R. Genetic pairwise distances and bootstrapping ######
################################################################
#7.1 R code for estimating Genetin pairwise distances
library ("ape")
tree <- read.tree("phylogenetic.tree") # upload tree
cophenetic.phylo(tree)   # all distancies between tips

#7.2 R code for performing the boostrapping
library (boot)
r <- read.delim (file="pairwise_distance_BF_GC",head=TRUE)
bf <- subset (r, type.FB.new<7)

grupo1 = c(bk$pairwise.B.F.)
s = sample(nrow(bf),nrow(bf),replace=T)
sample.median <- function(grupo1, s) median (grupo1[s], na.rm = TRUE)
boot.bf = boot(grupo1, sample.median, R=1000) # R is the number of times repiting the
sampling process with repetition.
quantile(boot.bf$t, c(.025,.975)) # Give you the statistic and the confidence of interval
```
